# Differential Effects of Linkers on the Activity of Amphiphilic Tobramycin Antifungals

**DOI:** 10.3390/molecules23040899

**Published:** 2018-04-13

**Authors:** Marina Y. Fosso, Sanjib K. Shrestha, Nishad Thamban Chandrika, Emily K. Dennis, Keith D. Green, Sylvie Garneau-Tsodikova

**Affiliations:** Department of Pharmaceutical Sciences, College of Pharmacy, University of Kentucky, Lexington, KY 40536-0596, USA; marina.fosso@uky.edu (M.Y.F.); sanjib.shrestha@uky.edu (S.K.S.); nishad.tc@uky.edu (N.T.C.); emily.dennis@uky.edu (E.K.D.); keith.green@uky.edu (K.D.G.)

**Keywords:** aminoglycosides, *Aspergillus*, *Candida*, *Cryptococcus*, cytotoxicity, hemolysis

## Abstract

As the threat associated with fungal infections continues to rise and the availability of antifungal drugs remains a concern, it becomes obvious that the need to bolster the antifungal armamentarium is urgent. Building from our previous findings of tobramycin (TOB) derivatives with antifungal activity, we further investigate the effects of various linkers on the biological activity of these aminoglycosides. Herein, we analyze how thioether, sulfone, triazole, amide, and ether functionalities affect the antifungal activity of alkylated TOB derivatives against 22 *Candida*, *Cryptococcus*, and *Aspergillus* species. We also evaluate their impact on the hemolysis of murine erythrocytes and the cytotoxicity against mammalian cell lines. While the triazole linker appears to confer optimal activity overall, all of the linkers incorporated into the TOB derivatives resulted in compounds that are very effective against the *Cryptococcus neoformans* species, with MIC values ranging from 0.48 to 3.9 μg/mL.

## 1. Introduction

Aminoglycosides (AGs) represent a group of structurally diverse amino-modified sugars that have long been used for their antibacterial efficacy. Their broad-spectrum of activity against a plethora of pathogenic bacteria has, indeed, made them a valuable class of antibiotics [1]. However, as is the case with most antibiotics nowadays, AGs are suffering from the resurgence of bacterial resistance [2]. To circumvent this problem, several research groups have devoted efforts to the development of novel AGs with improved antibacterial activity [3]. Amphiphilic AGs in particular, which are AG-derived cationic amphiphiles, emerged as potent antimicrobial agents with a new mechanism of action [4]. They resulted from the incorporation of various hydrophobic groups to the polycationic and hydrophilic amikacin [5], tobramycin (TOB) [6,7,8,9,10,11], neamine [12,13,14], neomycin B [15,16,17,18,19], paromomycin [20,21], and kanamycins A (KANA) and B (KANB) [22,23].

A growing interest in the identification of new targets of AGs [24] has recently led researchers to the investigation of AGs’ action against fungi [23,25,26,27,28,29,30]. It is worth mentioning that the social and economic burden associated with fungal infections is considerable, both in medicine and agriculture. While the frequency of invasive fungal infections continues to rise, the number of antifungal agents available remains limited, calling for the development of additional effective antifungal drugs. Indeed, only three classes of antifungals are currently in clinical use [31]. These include the polyenes (for example, amphotericin B (AmB)), the azoles (for example, fluconazole, voriconazole (VOR), itraconazole, posaconazole), and the echinocandins (for example, caspofungin (CAS), micafungin, anidulafungin). These drugs exert their antifungal activity by either extracting ergosterol from the fungal plasma membrane [32], blocking the production of ergosterol through the inhibition of lanosterol 14α-demethylase [33], or inhibiting the biosynthesis of the fungal cell membrane component (1,3)-β-d-glucan [34], respectively. Unfortunately, resistance to these antifungal drugs has already been observed [35]. However, new strategies to combat fungal infections are being investigated, notably the development of derivatives of currently approved antifungals with improved activity and biological safety profiles [36,37,38,39,40], the combination therapy of current antifungal drugs working synergistically with other drugs [41], and the development of new classes of antifungal agents [42].

We have recently demonstrated that incorporating a linear alkyl chain (for example, the dodecyl group (C_12_) or tetradecyl group (C_14_)) at the 6″-position of TOB or KANB through a thioether linkage results in AGs with antifungal activity against *C. albicans* [23,43]. Although we found the TOB (C_14_) derivative to display stronger antifungal activity than TOB (C_12_), we elected to generate TOB (C_12_) analogues in this study because, as we previously demonstrated by investigating the hemolytic activity of KANB (C_12_) and (C_14_), KANB (C_12_) displayed lower hemolytic activity than KANB (C_14_). Herein, with the goal of improving on the activity of TOB (C_12_), we investigated the effects of different groups (for example, thioether, sulfone, triazole, amide, and ether) as linkers for the alkyl chain to the AG scaffold on the efficacy of TOB (C_12_) derivatives against 22 fungal strains. We investigated the hemolytic activity and cytotoxicity of the five most promising antifungals. We also performed time-kill studies and membrane permeabilization assays for the most active compound generated.

## 2. Results and Discussion

### 2.1. Chemistry

**Scheme 1:**
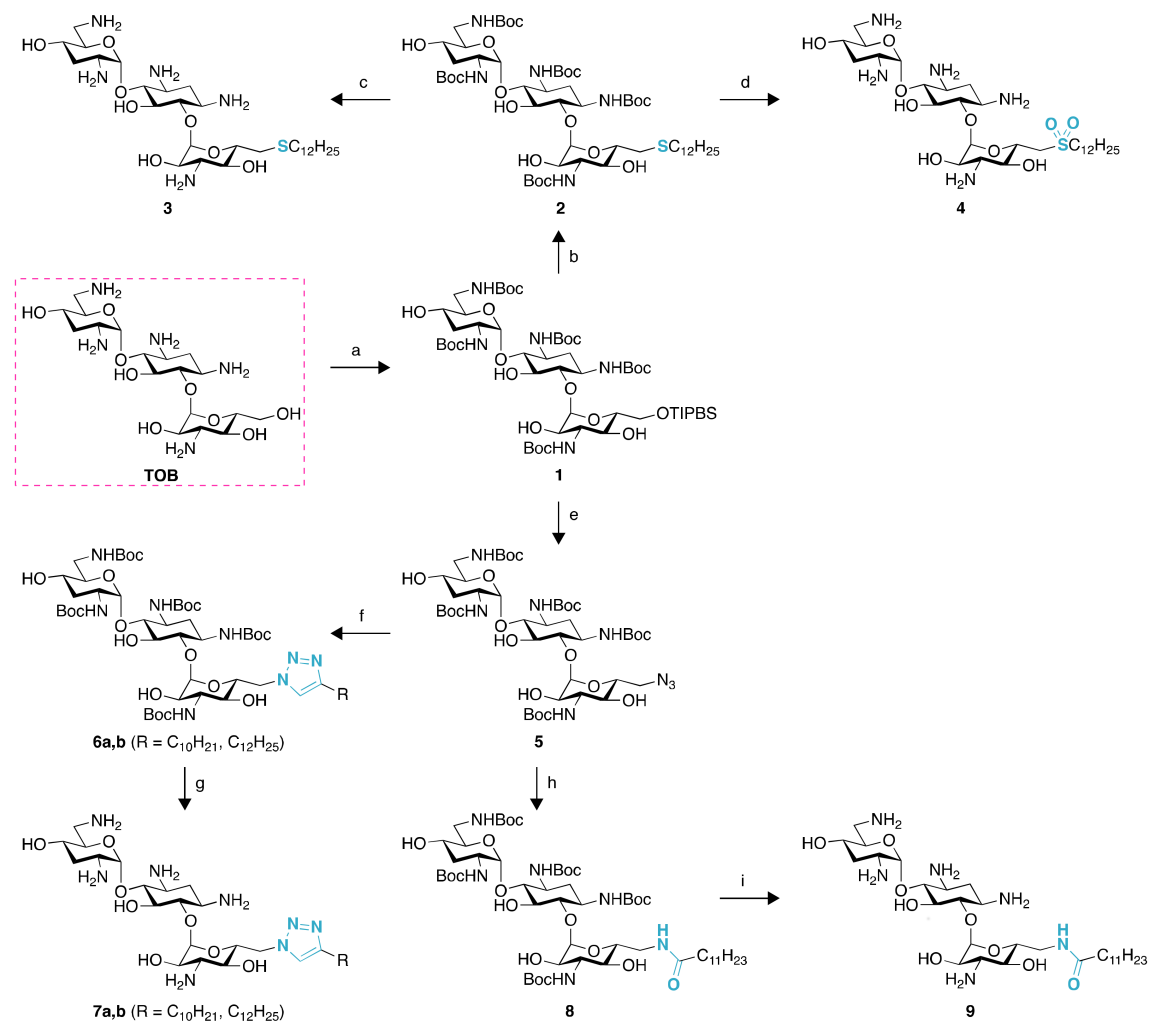
The synthesis of tobramycin (TOB) derivatives **3**, **4**, **7a**, **7b**, and **9**. Reagents: (**a**) TIPBSCl, pyridine, 59%; (**b**) C_12_H_25_SH, Cs_2_CO_3_, *N*,*N*-dimethylformamide (DMF), 79%; (**c**) Trifluoroacetic acid (TFA), 61%; (**d**) *m*-CPBA/CHCl_3_ then TFA, 66%; (**e**) Tetrabutylammonium azide (TBAA), DMF, 75 °C, 65%; (**f**) R-CCH, CuSO_4_·5H_2_O, sodium l-ascorbate, DMF, microwave, 57–63%; (**g**) TFA, 94%-quantitative; (**h**) PMe_3_, THF/H_2_O, 50 °C then C_11_H_23_CO_2_H, EDC·HCl, HOBt, DIPEA, DMF, 55%; (**i**) TFA, 93%.

The synthesis started from the commercially available AG TOB. Modification at the 6″-position of TOB requires the selective conversion of the 6″-hydroxyl group into a good leaving group, which could be accomplished by Boc protection of the amino groups of TOB, followed by a reaction with 2,4,6-triisopropylbenzenesulfonyl chloride (TIPBSCl) in pyridine (Scheme 1). This gave the central intermediate **1** [44], which upon treatment with 1-dodecanethiol and Cs_2_CO_3_, afforded another intermediate compound **2** [8,10]. Treatment of compound **2** with trifluoroacetic acid (TFA) efficiently removed all the Boc protecting groups to give the target compound **3** [10], representing the TOB derivative with the thioether linkage. Furthermore, *S*-oxidation of compound **2** with *m*-CPBA followed by TFA treatment yielded the target sulfone derivative **4** [8].

Compound **1** was also subjected to a S_N_2 nucleophilic displacement reaction in the presence of tetrabutylammonium azide (TBAA) to give the azido intermediate **5** [11]. Using copper sulfate and sodium l-ascorbate in DMF under microwave conditions, a click reaction between compound **5** and commercially available alkynes (1-dodecyne and 1-tetradecyne) afforded the intermediates **6a** and **6b** [11], respectively, whose TFA treatment gave the target triazole derivatives **7a** and **7b** [11], respectively. The design for compound **7a** (C_10_-triazole), which is two-carbon shorter than compound **7b**, stems from the need to investigate whether or not the nitrogen atoms N2 and N3 of the triazole ring contribute to the overall length of the alkyl side chain. Compound **5** was also subjected to Staudinger reduction that converted the 6″-azido group into an amine, followed by amide coupling with lauric acid to yield compound **8**, which was then converted to the target amide derivative **9**.

**Scheme 2:**
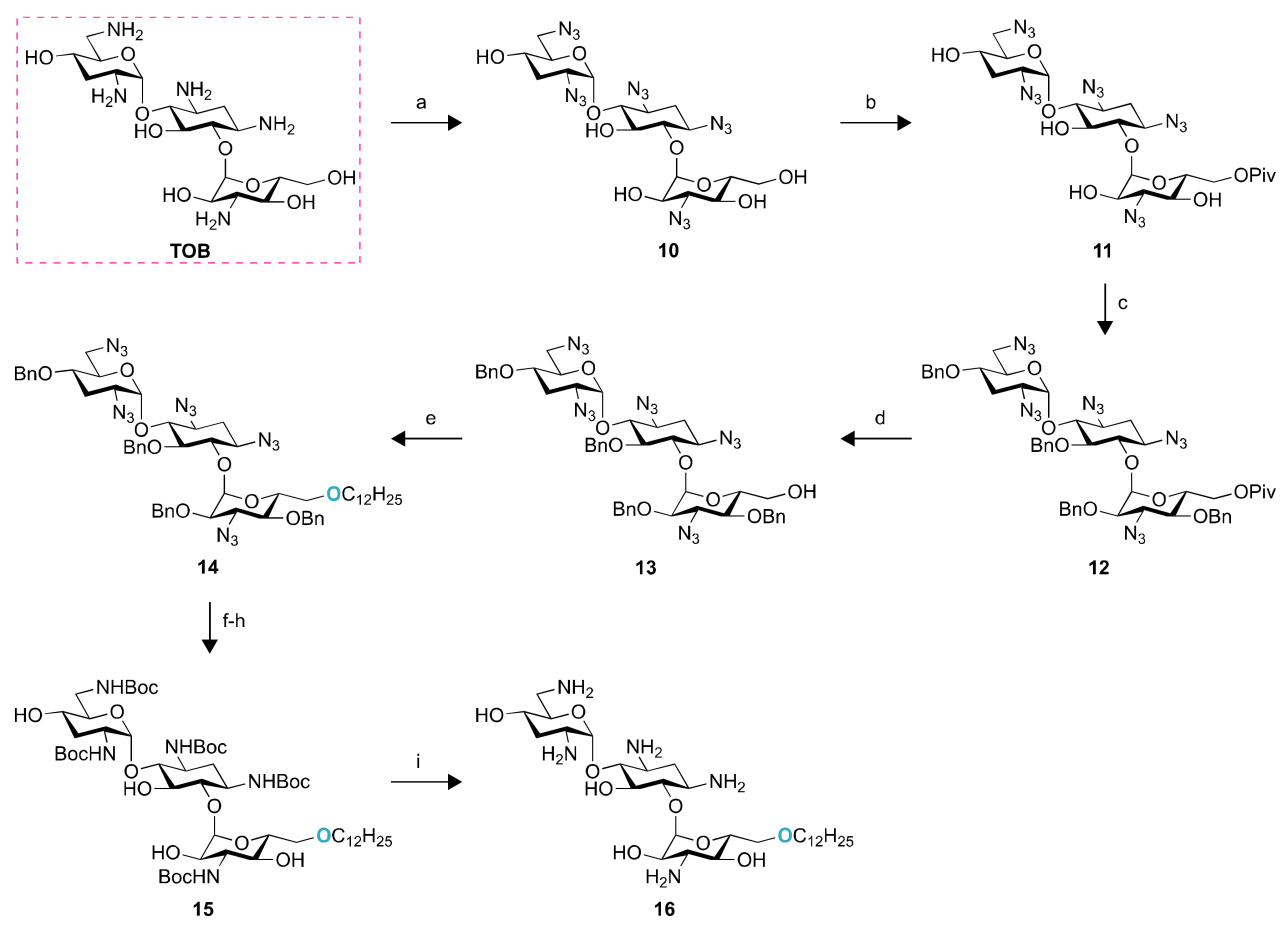
The synthesis of TOB derivative **16**. Reagents: (**a**) NaN_3_, Tf_2_O, H_2_O, CH_2_Cl_2_ then ZnCl_2_, Et_3_N, H_2_O, MeOH, 92%; (**b**) PivCl, pyridine, 0 °C, 63%; (**c**) NaH, BnBr, TBAI, DMF, 76%; (**d**) Na, MeOH, 60 °C, 81%; (**e**) NaH, C_12_H_25_Br, TBAI, DMF, 82%; (**f**) PMe_3_, NaOH, THF; (**g**) Pd(OH)_2_/C, H_2_, AcOH, H_2_O; (**h**) Boc_2_O, Et_3_N, THF, 60 °C, 32% over 3 steps; (**i**) TFA, 96%.

The synthesis of the TOB derivative with the ether linkage started with the conversion of TOB into the known perazide **10** [45] with triflyl azide, which was then reacted with pivaloyl chloride to afford the 6″-*O*-pivalate **11** (Scheme 2). Benzyl protection of the remaining hydroxyl groups afforded compound **12**, which was then subjected to base hydrolysis of the pivaloyl group to give compound **13**, with a free 6″-hydroxyl group. Alkylation of compound **13** in the presence of sodium hydride and 1-bromododecane then afforded compound **14**. Staudinger reduction of azide to amine, hydrogenolysis, and Boc protection of the free amino groups gave compound **15**, which was converted to the target 6″-*O*-dodecyl-TOB derivative **16** through TFA deprotection.

### 2.2. In Vitro Antifungal Activity

The minimum inhibitory concentration (MIC) values of TOB and its derivatives **3**, **4**, **7a**, **7b**, **9**, and **16** were determined (Table 1, Table 2 and Table 3). We focused our study on the *Candida* (*albicans* and non-*albicans*), *Cryptococcus*, and *Aspergillus* species, which are medically significant fungal pathogens [46]. We also included the FDA-approved antifungal drug CAS as a control; it is used clinically in the treatment of fungal infections caused by *Candida* and *Aspergillus* species.

Our initial study targeted the *C. albicans* species since, in addition to offering a point of comparison with our previously published data on compound **3** [43], they are responsible for the largest number of *Candida*-related fungal infections in humans [47]. They account for many invasive fungal infections observed in intensive care units [48]. These included the following seven strains: *C. albicans* ATCC 10231 (strain **A**), *C. albicans* ATCC 64124 (strain **B**), *C. albicans* ATCC MYA-2876 (strain **C**), *C. albicans* ATCC 90819 (strain **D**), *C. albicans* ATCC MYA-2310 (strain **E**), *C. albicans* ATCC MYA-1237 (strain **F**), and *C. albicans* ATCC MYA-1003 (strain **G**). While the derivatives appeared to be less effective than CAS, all but compound **16** were better than the parent TOB against all *C. albicans* strains tested (Table 1). Indeed, TOB was inactive against all strains tested with MIC values >62.5 μg/mL. On the other hand, compound **7b**, with the triazole linkage, was the most active derivative with MIC values ranging from 3.9 to 15.6 μg/mL, which corresponds to a 4- to >16-fold improvement compared to TOB. The thioether derivative **3** and the sulfone **4** were the next most active derivatives. The MIC values for compound **3** corresponded within 2-fold to those previously published [43]. The MIC values of compounds **3** and **4** (7.8–31.3 μg/mL) were also not much different from each other, always within a 2-fold dilution, indicating that metabolic *S*-oxidation might not have a noticeable effect on potency. This is in agreement with our previous observation of KANB with a C_12_ chain in a thioether linkage at the 6″-position and its oxidized counterpart as antifungals [23]. Compound **16** (C_12_-ether) was the least effective (MIC values ≥62.5 μg/mL) followed by the C_10_-triazole **7a** (MIC values ranging from 31.3 to 62.5 μg/mL) and the C_11_-amide **9** (MIC values ranging from 15.6 to 62.5 μg/mL).

These promising results encouraged us to expand our study to nine non-*albicans Candida* strains, including *C. glabrata* ATCC 2001 (strain **H**), *C. krusei* ATCC 6258 (strain **I**), *C. parapsilosis* ATCC 22019 (strain **J**), three *C. glabrata* clinical isolates (**CG1**–**3**), and three *C. parapsilosis* clinical isolates (**CP1**–**3**) (Table 2). As observed with the *C. albicans* yeast strains, the compounds **3**, **4**, **7a**, **7b**, **9**, and **16** were more active than TOB, but less active than CAS. Once again, compound **7b**, with the triazole linkage, displayed the lowest MIC values overall (1.95–7.8 μg/mL), representing an 8- to >32-fold improvement compared to TOB (MIC values >62.5 μg/mL). The thioether **3** and the sulfone **4** also exhibited good activity (MIC values ranging from 3.9 to 15.6 μg/mL), followed by the amide **9** (MIC values ranging from 7.8 to 31.3 μg/mL), the C_10_-triazole **7a** (MIC values ranging from 15.6 to 62.5 μg/mL), and the C_12_-ether **16** (MIC values ranging from 31.3 to 62.5 μg/mL).

We had previously observed that *Cryptococcus neoformans* MYA-895 was exceptionally sensitive to compound **3**, with a low MIC value of 1.95 μg/mL [43]. We thus decided to assess the potency of compounds **3**, **4**, **7a**, **7b**, **9**, and **16** against *C. neoformans* clinical isolates **CN1**–**3** (Table 2). Interestingly, all synthesized TOB derivatives displayed excellent activity, with MIC values ranging from 0.48 to 3.9 μg/mL, while TOB and CAS showed low to no activity. This represents a 16- to >128-fold improvement compared to TOB (MIC values >62.5 μg/mL). *C. neoformans* is responsible for cryptococcosis, which remains a significant cause of mortality and morbidity in immunocompromised individuals [49].

Finally, based on the very promising and encouraging results obtained against the *C. neoformans* clinical isolates, we decided to assess the antifungal activity of the derivatives generated against other types of fungal strains. We opted for the filamentous fungi *Aspergillus flavus* ATCC MYA-3631 (strain **K**), *Aspergillus nidulans* ATCC 38163 (strain **L**), and *Aspergillus terreus* ATCC MYA-3633 (strain **M**) (Table 3). In these cases, all the TOB derivatives, with the exception of compound **16**, were better than both TOB and CAS, with a trend similar to that observed with the *C. albicans* and non-*albicans Candida* strains. Furthermore, while *A. terreus* ATCC MYA-3633 (strain **M**) was not sensitive to the TOB derivatives, compounds **3**, **4**, **7a**, **7b**, and **9** showed moderate activity against *A. flavus* ATCC MYA-3631 (strain **K**) (MIC values ranging from 7.8 to 31.3 μg/mL) and good activity against *A. nidulans* ATCC 38163 (strain **L**) (MIC values ranging from 1.95 to 3.9 μg/mL). Compound **16** only showed good activity against *A. nidulans* ATCC 38163 (strain **L**) with an MIC value of 3.9 μg/mL.

In light of these results, it appears that the linkers play a major role in the activity of alkylated TOB derivatives, with triazole > “better than” thioether/sulfone > amide > ether. This may stem from the distinct ability of these linkers to form interactions with the polar lipid head groups of fungi. Indeed, with its three nitrogen atoms, the triazole ring may form more hydrogen bonds than the sulfone which has two oxygens, the amide with a nitrogen and an oxygen, and the ether with only an oxygen atom. It is worth mentioning that, while compound **7b** (C_12_-triazole) was the most effective at inhibiting the growth of yeasts and filamentous fungi, compound **7a** (C_10_-triazole) was the least effective of all six TOB derivatives synthesized (except compound **16**). This is no surprise as it is in accordance with the chain length-dependent antifungal activity observed with other amphiphilic AGs [23,43]. Indeed, amphiphilic AGs bear a hydrophilic core structure, which is rich in polar hydroxyl and amino groups that are positively charged under physiological conditions, and a lipophilic alkyl chain capable of interacting with the lipid-rich fungal cell membrane. As the length of the alkyl chain increases, the ability of amphiphilic AGs to puncture the lipid bilayers also increases, which may result in an enhanced cell membrane perturbation, a mechanism well-known for this type of molecules [8,27]. This also shows that the additional atoms N2 and N3 of the triazole ring do not contribute to the overall length of the alkyl chain.

### 2.3. Hemolysis

**Figure 1:**
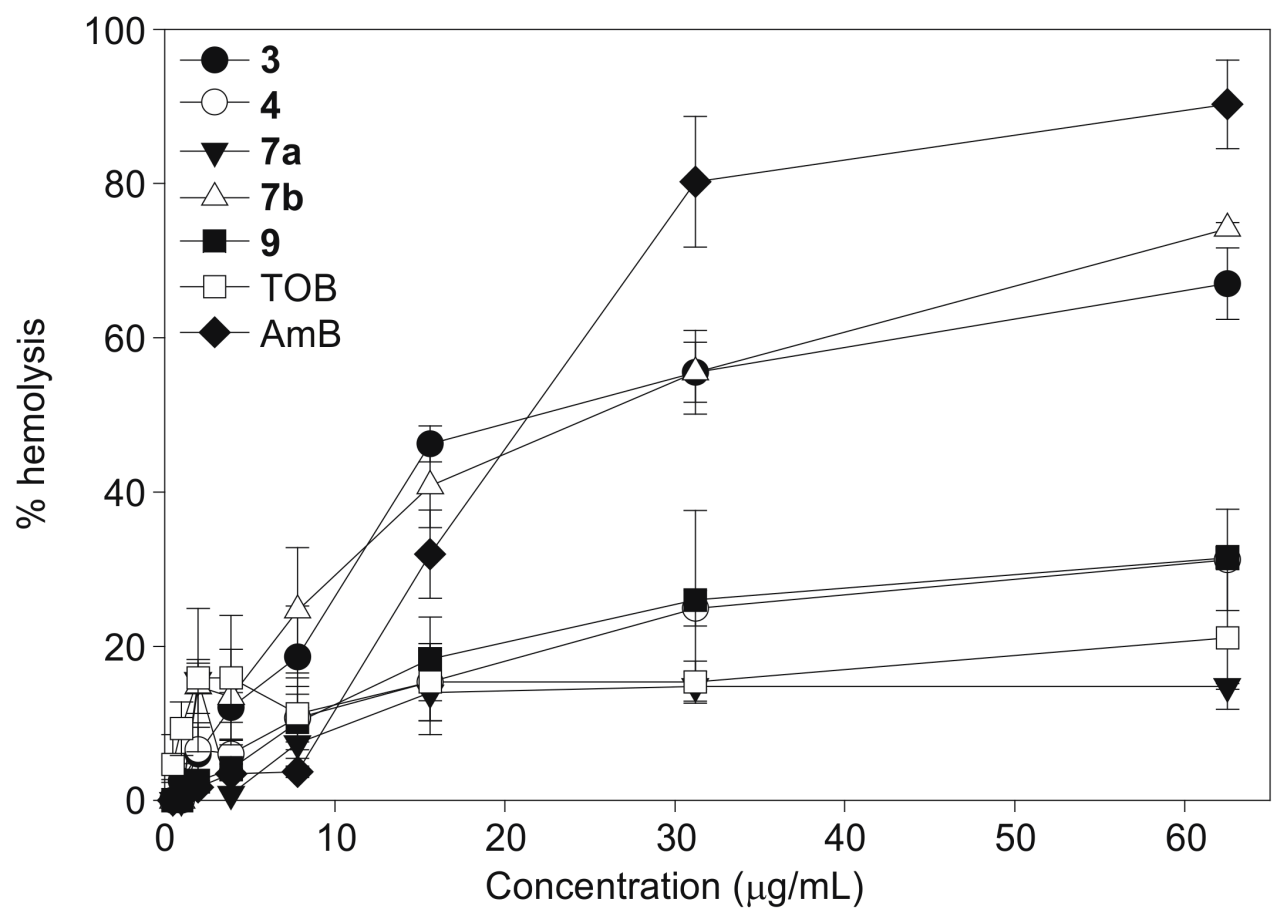
The hemolytic activity of tobramycin (TOB); its derivatives **3**, **4**, **7a**, **7b**, and **9**; as well as AmB on mRBCs. Note: the exact values used to generate this graph are presented in Table S1.

As potential antifungal agents, it is necessary to assess the ability of the synthesized compounds to selectively target fungal membranes. Since compound **16** was in general as inactive as the parent AG TOB, we decided to focus our efforts on the remaining TOB derivatives. We performed a hemolytic assay of the active TOB derivatives **3**, **4**, **7a**, **7b**, and **9** using murine red blood cells (mRBCs) (Figure 1 and Table S1). Although these amphiphilic TOB derivatives appear to affect mRBCs more than the parent TOB, they all displayed relatively lower hemolytic activity compared to AmB, which is an FDA-approved antifungal prescription medicine. Overall, the following trend was observed: **7a** (C_10_-triazole) < “less hemolytic than” **9** (C_11_-amide) < **4** (C_12_-sulfone) < **3** (C_12_-thioether) < **7b** (C_12_-triazole). Once again, we noticed a chain length-dependent effect on hemolysis, as previously observed with KANB-derived cationic amphiphiles [23]. It also appeared that the sulfone and amide linkers may impart less hemolysis than the thioether and triazole ones. Oxidation of the thioether derivative **3** to its corresponding sulfone **4** also seemed to lessen the hemolytic activity, suggesting that metabolic *S*-oxidation may not be detrimental in this case. While the sulfone derivative **4** and the amide **9** lysed ~15–25% and ~18–32% of mRBCs, respectively, at their MIC values against all seven *C. albicans* strains, the thioether derivative **3** and the triazole **7b** lysed ~19–46% and ~13–41% of mRBCs, respectively. At their MIC values against non-*albicans Candida* strains, the sulfone derivative **4** and the amide **9** lysed ~6–11% and ~18–26% of mRBCs, respectively, while the thioether derivative **3** and the triazole **7b** lysed ~19–46% and ~14–25% of mRBCs, respectively. While more hemolysis was observed at their MIC values against the *Aspergillus* strains (~4–32% for compounds **4** and **9**, and ~12–74% for compounds **3** and **7b**), the TOB derivatives **3**, **4**, **7a**, **7b**, and **9** caused little to no hemolysis when tested at their MIC values against *C. neoformans*, only lysing up to 13% of mRBCs at 3.9 μg/mL, which represents 2- to 8-fold their MIC values against the three clinical isolates tested. The generally low hemolytic activity observed strengthens the potential application of the newly synthesized TOB derivatives as antifungal agents against the *C. neoformans* species.

### 2.4. Cytotoxicity

**Figure 2:**
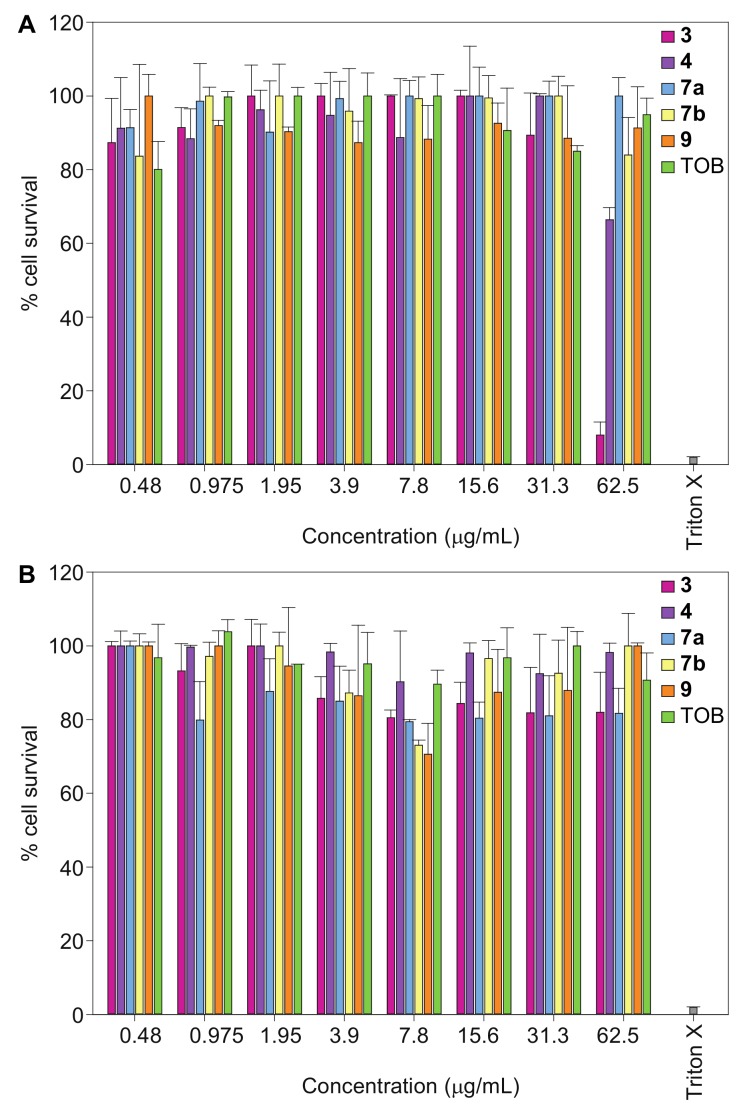
The mammalian cell cytotoxicity of TOB and its derivatives **3**, **4**, **7a**, **7b**, and **9** against (**A**) the BEAS-2B cell line and (**B**) the A549 cell line.

To further evaluate the potential safety of the TOB derivatives **3**, **4**, **7a**, **7b**, and **9**, we assessed the cytotoxicity of these compounds against two mammalian cell lines, BEAS-2B and A549 (Figure 2). Like the parent AG TOB, these derivatives displayed little to no toxicity against both cell lines. Indeed, compounds **4** (sulfone), **7a** (C_10_-triazole), **7b** (C_12_-triazole), and **9** (amide) all had IC_50_ values >62.5 μg/mL, which were 1- to 16-fold higher than their antifungal MIC values against the *C. albicans* species. Only compound **3** (thioether) had an IC_50_ value in the range of 31.3–62.5 μg/mL in BEAS-2B cells. This observed selectivity of the TOB derivatives in targeting fungal cells in the presence of mammalian cells may stem from the difference in lipid composition of the cell membranes in fungi and humans [50].

### 2.5. Time-Kill Studies

**Figure 3:**
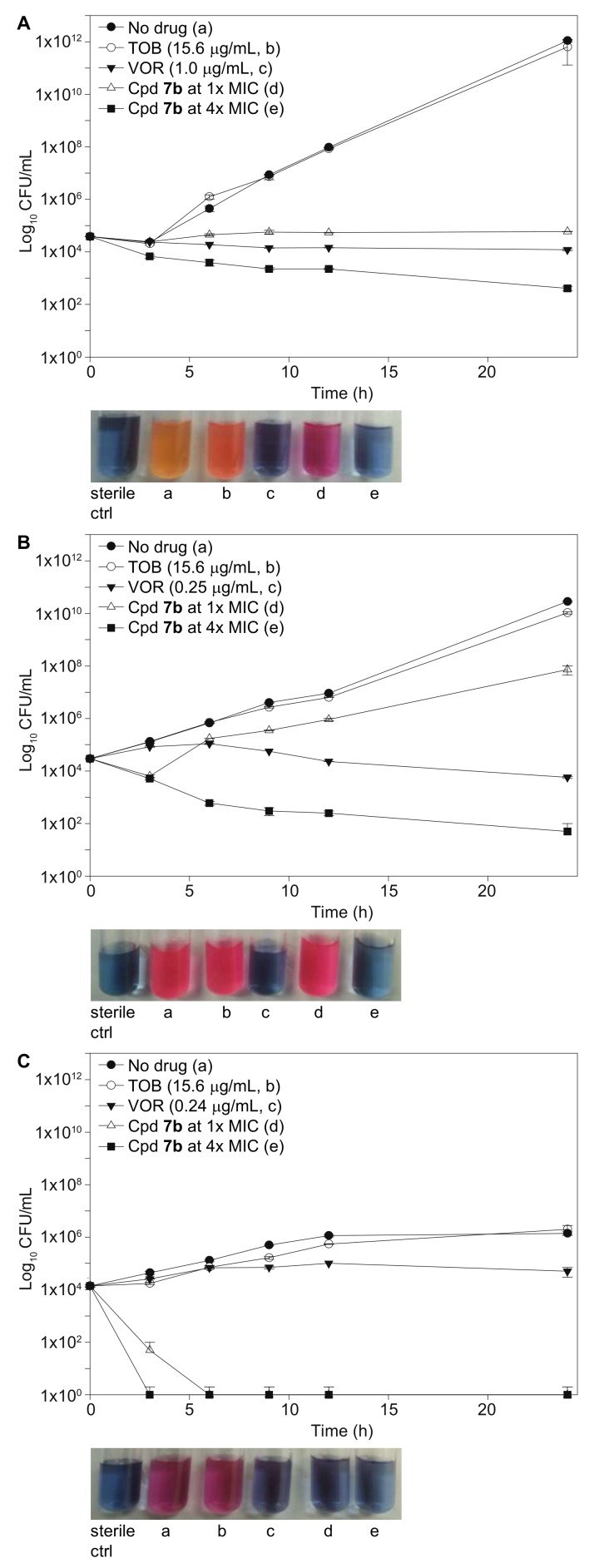
The representative time-kill studies of TOB derivative **7b** against (**A**) *C. albicans* ATCC 10231 (strain **A**), (**B**) *C. parapsilosis* ATCC 22019 (strain **J**), and (**C**) *Cryptococcus neoformans* clinical isolate **CN1**. In all panels, the cultures were exposed to a no drug control (black circle), TOB at 15.6 μg/mL (white circle), VOR at 1.0 or 0.25 μg/mL (black inverted triangle), and **7b** at 1× MIC (white triangle) and 4× MIC (black square). Each test tube represents the corresponding sample treated with resazurin, which was added for the visualization of fungal growth. Note: Blue = no fungal growth; orange-pink = fungal growth.

In light of the results presented so far, we selected the most promising compound **7b** for further investigations. We evaluated the antifungal potency of **7b** (C_12_-triazole) by determining its time-kill course against a representative *C. albicans* strain, ATCC 10231 (strain **A**); a representative non-*albicans Candida* strain, *C. parapsilosis* ATCC 22019 (strain **J**); and a representative non-*Candida* yeast, *C. neoformans* (strain **CN1**) over a 24-h period. We also included the parent AG TOB and the triazole antifungal agent VOR as controls in our study (Figure 3). While TOB did not affect the growth of any of the tested fungal strains, its triazole derivative **7b** was able to reduce the *C. albicans* ATCC 10231 (strain **A**) CFU by ≥2 log_10_ after 9 h of treatment at 3.9 μg/mL (1× MIC) and after 6 h of treatment at 15.6 μg/mL (4× MIC) (Figure 3a). This suggests a dose-dependent effect as previously observed with other antifungal amphiphilic AGs [23,43]. Furthermore, compound **7b** showed a similar growth inhibitory effect to that of VOR, since the latter also reduced the CFU of *C. albicans* ATCC 10231 (strain **A**) by ≥2 log_10_ after 9 h of treatment at its 1× MIC value of 1.0 μg/mL and maintained a fungistatic activity up to the 24-h period. Against *C. parapsilosis* ATCC 22019 (strain **J**), compound **7b** displayed a fungistatic effect at 1.95 μg/mL (1× MIC) only for the first 3 h before the fungal cells followed a trend similar to the growth control (Figure 3b). A decrease in CFU by ≥2 log_10_ was only observed after 24 h of treatment. Meanwhile, at 7.8 μg/mL (4× MIC), compound **7b** completely killed *C. parapsilosis* ATCC 22019 (strain **J**) after 24 h. At 0.49 μg/mL (1× MIC), compound **7b** rapidly reduced the CFU of *C. neoformans* (strain **CN1**) by ≥2 log_10_ after 3 h of treatment (Figure 3c). In addition, complete fungal cell death was observed as early as 6 h after the treatment at 0.49 μg/mL (1× MIC) and 3 h after the treatment at 1.95 μg/mL (4× MIC), while TOB and VOR showed little to no growth inhibitory effect against *C. neoformans* (strain **CN1**). These results confirm that the TOB derivative **7b** (C_12_-triazole) exhibits better fungal growth inhibition than the parent AG TOB. Furthermore, compound **7b** displayed a similar fungistatic effect to that of the FDA-approved triazole antifungal agent VOR against *C. albicans* ATCC 10231 (strain **A**) and *C. parapsilosis* ATCC 22019 (strain **J**), and an enhanced fungicidal effect against *C. neoformans* (strain **CN1**). The promise of **7b** as a fungicidal agent against *Cryptococcus* sp. is highly encouraging.

### 2.6. Antifungal Mechanism of Action

**Figure 4:**
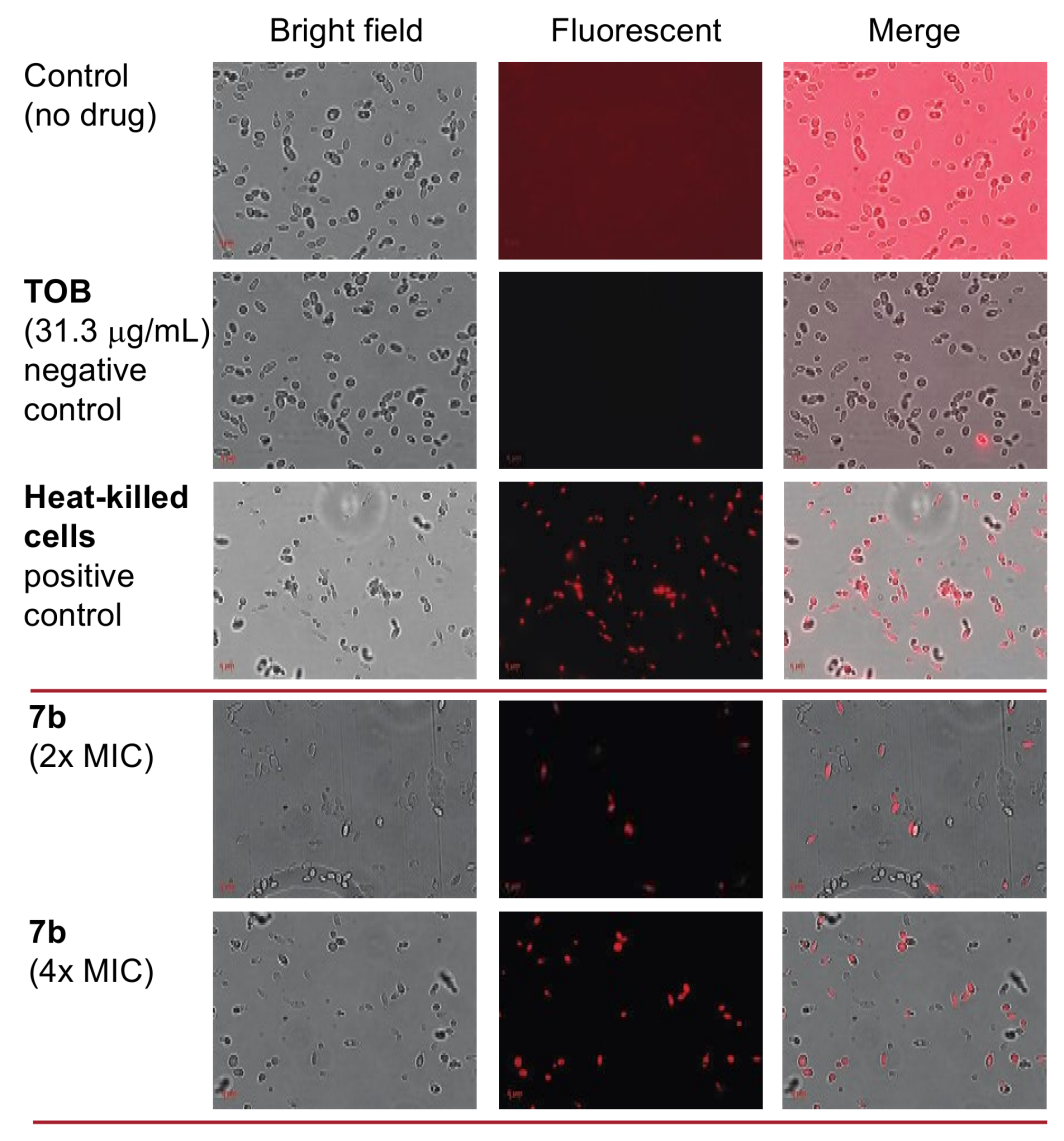
The representative dose-dependent membrane permeabilization effects of TOB and its derivative **7b** on *C. albicans* ATCC 10231 (strain **A**). From top to bottom: propidium iodine (PI) dye uptake by yeast cells without drug, with TOB (31.3 μg/mL), killed by heat, with **7b** (2× MIC), and with **7b** (4× MIC).

We have previously shown that amphiphilic TOB derivatives, notably compound **3**, exert their antifungal activity by perturbing the fungal cell membrane [43]. We then decided to evaluate the ability of the most potent compound **7b** to affect the fungal membrane integrity. Using propidium iodide (PI), a dye that fluoresces upon binding nucleic acids in membrane-compromised cells [51], we observed that *C. albicans* ATCC 10231 (strain **A**) cells that were treated with compound **7b** at concentrations equivalent to 2× MIC or 4× MIC allowed a larger influx of PI dye compared to the untreated cells or those treated with the parent TOB, which only showed negligible staining (Figure 4). Yeast killed by heat were used as the positive control. These results show that compound **7b** also affects the fungal membrane permeability and is more effective than TOB in causing PI permeation into the azole-resistant *C. albicans* ATCC 10231 (strain **A**) cells.

## 3. Conclusions

In summary, we have synthesized six TOB derivatives with various linkers, including thioether, sulfone, triazole, amide, and ether. While the C_12_-triazole derivative **7b** was the most potent, the C_12_-ether derivative **16** was the least effective at inhibiting the growth of several fungal strains. We also noticed a chain length-dependent effect on the antifungal activity. Furthermore, the active TOB derivatives displayed low hemolytic activity and low mammalian cell toxicity. Finally, the compounds generated were very active against *C. neoformans* clinical isolates, leaving the door open for a future (outside of the scope of this study) investigative avenue for the development of novel therapies for cryptococcosis.

## 4. Materials and Methods

### 4.1. General Information

Tobramycin (TOB) was purchased from AK Scientific (Union City, CA, USA). All other chemicals were purchased from Sigma-Aldrich (St. Louis, MO, USA) and used without further purification. Compounds **1** [44], **5** [11], and **10** [45] were prepared as previously described. All microwave irradiation experiments were performed in a sealed glass microwave reaction vial using a Biotage^®^ Initiator™ microwave synthesizer. Experiments were performed in temperature-control mode where the temperature was controlled using the built-in calibrated IR sensor. Chemical reactions were monitored by TLC (Merck, Kenilworth, NJ, USA, Silica gel 60 F_254_). Visualization was achieved using one of the following methods: H_2_SO_4_ stain (5% in MeOH), ceric molybdate stain (5 g (NH_4_)_2_Ce(NO_3_)_6_, 120 g (NH_4_)_6_Mo_7_O_24_·4H_2_O, 80 mL H_2_SO_4_, and 720 mL H_2_O), or *p*-anisaldehyde stain (54 mL EtOH, 3 mL H_2_SO_4_, and 3 mL *p*-anisaldehyde). Compounds were purified by SiO_2_ flash chromatography (Dynamic Adsorbents Inc., Norcross, GA, USA, Flash silica gel 32–62u). ^1^H and ^13^C-NMR spectra were recorded on a Varian 400 MHz spectrometer. High-resolution mass sprectra were recorded on an AB SCIEX Triple TOF 5600 System. All compounds tested for activity are ≥95% pure according to NMR spectra. TOB derivatives **3**, **4**, **7a**, **7b**, **9**, and **16** were dissolved in sterile MQ-H_2_O at a final concentration of 10 mg/mL and stored at −20 °C.

The antifungal agent caspofungin (CAS) was purchased from Sigma-Aldrich (St. Louis, MO, USA), dissolved in DMSO at a final concentration of 5 mg/mL, and stored at −20 °C. *Candida albicans* ATCC 10231 (strain **A**), *C. albicans* ATCC 64124 (strain **B**), and *C. albicans* ATCC MYA-2876 (strain **C**) were kindly provided by Dr. Jon Y. Takemoto (Utah State University, Logan, UT, USA). *C. albicans* ATCC MYA-90819 (strain **D**), *C. albicans* ATCC MYA-2310 (strain **E**), *C. albicans* ATCC MYA-1237 (strain **F**), *C. albicans* ATCC MYA-1003 (strain **G**), *Candida glabrata* ATCC 2001 (strain **H**), *Candida krusei* ATCC 6258 (strain **I**), *Candida parapsilosis* ATCC 22019 (strain **J**), *Aspergillus flavus* ATCC MYA-3631 (strain **K**), and *Aspergillus terreus* ATCC MYA-3633 (strain **M**) were obtained from the American Type Culture Collection (ATCC; Manassas, VA, USA). *Aspergillus nidulans* ATCC 38163 (strain **N**) was received from Dr. Jon S. Thorson (University of Kentucky, Lexington, KY, USA). All clinical fungal isolates, *C*. *glabrata* (strain **CG1**–**3**), *C*. *parapsilosis* (strain **CP1**–**3**), and *Cryptococcus neoformans* (strain **CN1**–**3**) were obtained from Dr. Nathan P. Wiederhold (University of Texas Health Science Center, San Antonio, TX, USA). Filamentous fungi and yeasts were cultivated at 35 °C in RPMI 1640 medium (with l-glutamine, without sodium biocarbonate, Sigma-Aldrich, St. Louis, MO, USA) buffered to a pH of 7.0 with 0.165 M morpholinepropanesulfonic acid (MOPS) buffer (Sigma-Aldrich).

The human bronchus normal cell line BEAS-2B (ATCC CRL-9609) and the human lung carcinoma cell line A549 (ATCC CRL-185) were kind gifts from the laboratories of Dr. David K. Orren (University of Kentucky, Lexington, KY, USA) and Dr. Markos Leggas (University of Kentucky, Lexington, KY, USA). The mammalian cells were grown in Dulbecco’s Modified Eagle’s Medium (DMEM) (from ATCC) with 10% fetal bovine serum (FBS) (from ATCC) and 1% Pen/Strep (from ATCC). The cell lines were incubated at 37 °C and 5% CO_2_ and passaged by trypsinization with 0.05% trypsin and 0.53 mM EDTA (from ATCC). The cell confluency was determined by using a Nikon Eclipse TS100 microscope (Minato, Tokyo, Japan).

### 4.2. Synthesis of Compounds **2**–**16**

*O*-3-Deoxy-3-[[(1,1-dimethylethoxy)carbonyl]amino]-6-*S*-dodecyl-6-thio-α-d-glucopyranosyl-(1→6)-*O*-[2,3,6-trideoxy-2,6-bis[[(1,1-dimethylethoxy)carbonyl]amino]-α-d-*ribo*-hexopyranosyl-(1→4)]-2-deoxy-*N*^1^,*N*^3^-bis[(1,1-dimethylethoxy)carbonyl]-d-streptamine (**2**). A solution of compound **1** (0.30 g, 0.24 mmol), Cs_2_CO_3_ (0.12 g, 0.36 mmol), and 1-dodecanethiol (0.29 mL, 1.22 mmol) in anhydrous DMF (3 mL) was stirred at room temperature overnight. Progress of the reaction was monitored by TLC (Hexanes:EtOAc/2:3, R*_f_* 0.56). Purification by flash column chromatography (SiO_2_, pure Hexanes to Hexanes:EtOAc/2:3) gave the known compound **2** [8] (0.18 g, 64%) as a white solid: ^1^H-NMR (400 MHz, CD_3_OD, which matches the Reference [8], Figure S1) δ 5.07 (br s, 1H, H-1′), 5.01 (br d, *J* = 2.8 Hz, 1H, H-1″), 4.05 (ddd, *J*_1_ = 9.2 Hz, *J*_2_ = 7.6 Hz, *J*_3_ = 2.8 Hz, 1H, H-5″), 3.70–3.30 (m, 13H, H-1, H-3, H-4, H-5, H-6, H-2′, H-4′, H-5′, H-6′ (2H), H-2″, H-3″, H-4″), 2.97 (dd, *J*_1_ = 14.4 Hz, *J*_2_ = 2.8 Hz, 1H, H-6″), 2.61 (m, 1H, H-6″), 2.57 (t, *J* = 7.2 Hz, 2H, SCH_2_(CH_2_)_10_CH_3_), 2.14 (m, 1H, H-2eq), 1.99 (m, 1H, H-3′eq), 1.62 (app. q, *J*_1_ = *J*_2_ = *J*_3_ = 12.0 Hz, 1H, H-3′ax), 1.59–1.22 (m, 66H, H-2ax, 5× CO_2_(CH_3_)_3,_ SCH_2_(CH_2_)_10_CH_3_), 0.88 (t, *J* = 7.2 Hz, 3H, SCH_2_(CH_2_)_10_CH_3_).

*O*-3-Amino-3-deoxy-6-*S*-dodecyl-6-thio-α-d-glucopyranosyl-(1→6)-*O*-[2,6-diamino-2,3,6-trideoxy-α-d-*ribo*-hexopyranosyl-(1→4)]-2-deoxy-d-streptamine (**3**). Compound **2** (26 mg, 0.023 mmol) was treated at room temperature with neat TFA (1 mL) for 3 min. The TFA was removed under reduced pressure, the residue was dissolved in a minimal volume of H_2_O and freeze-dried to afford the known compound **3** [8] (17 mg, 61%) as a white powder: ^1^H-NMR (400 MHz, D_2_O, which matches Reference [8], Figure S2) δ 5.60 (d, *J* = 3.6 Hz, 1H, H-1′), 4.95 (d, *J* = 4.0 Hz, 1H, H-1″), 3.90–3.77 (m, 4H, H-4, H-5′, H-2″, H-5″), 3.75 (app. t, *J*_1_ = *J*_2_ = 9.2 Hz, 1H, H-5), 3.65 (app. t, *J*_1_ = *J*_2_ = 9.6 Hz, 1H, H-6), 3.61–3.38 (m, 5H, H-1, H-3, H-2′, H-4′, H-4″), 3.32 (app. t, *J*_1_ = *J*_2_ = 10.8 Hz, 1H, H-3″), 3.29 (dd, *J*_1_ = 13.6 Hz, *J*_2_ = 3.6 Hz, 1H, H-6′), 3.13 (dd, *J*_1_ = 14.0 Hz, *J*_2_ = 7.2 Hz, 1H, H-6′), 2.91 (dd, *J*_1_ = 14.0 Hz, *J*_2_ = 2.0 Hz, 1H, H-6″), 2.63 (dd, *J*_1_ = 14.0 Hz, *J*_2_ = 8.0 Hz, 1H, H-6″), 2.48 (t, *J* = 7.6 Hz, 2H, SCH_2_(CH_2_)_10_CH_3_), 2.42 (app. dt, *J*_1_ = 12.4 Hz, *J*_2_ = *J*_3_ = 4.4 Hz, 1H, H-2eq), 2.17 (app. dt, *J*_1_ = 12.4 Hz, *J*_2_ = *J*_3_ = 4.4 Hz, 1H, H-3′eq), 1.88 (app. q, *J*_1_ = *J*_2_ = *J*_3_ = 12.0 Hz, 1H, H-3′ax), 1.80 (app. q, *J*_1_ = *J*_2_ = *J*_3_ = 12.4 Hz, 1H, H-2ax), 1.45 (p, *J* = 7.2 Hz, 2H, SCH_2_CH_2_(CH_2_)_9_CH_3_), 1.22–1.06 (m, 18H, SCH_2_CH_2_(CH_2_)_9_CH_3_), 0.72 (t, *J* = 7.2 Hz, 3H, SCH_2_(CH_2_)_10_CH_3_).

*O*-3-Amino-3,6-dideoxy-6-(dodecylsulfonyl)-α-d-glucopyranosyl-(1→6)-*O*-[2,6-diamino-2,3,6-trideoxy-α-d-*ribo*-hexopyranosyl-(1→4)]-2-deoxy-d-streptamine (**4**). *m*-Chloroperbenzoic acid (*m*-CPBA, 30 mg, 0.17 mmol) was added to a solution of compound **2** (56 mg, 0.049 mmol) in CHCl_3_ (3 mL) and the resulting mixture was stirred at room temperature for 16 h. The reaction mixture was then diluted with CHCl_3_ (10 mL) and washed with 1M KOH (3 × 2 mL). The organic layers were dried over anhydrous MgSO_4_, filtered, and concentrated under reduced pressure. The crude product obtained was treated at room temperature with neat TFA (0.5 mL) for 3 min. The TFA was removed under reduced pressure, the residue was dissolved in a minimal volume of H_2_O and freeze-dried to afford the known compound **4** [8] (40 mg, 66%) as a white foam: ^1^H-NMR (400 MHz, D_2_O, which matches the Reference [8], Figure S3) δ 5.54 (d, *J* = 3.6 Hz, 1H, H-1′), 5.00 (d, *J* = 3.6 Hz, 1H, H-1″), 4.30 (app. t, *J*_1_ = *J*_2_ = 9.6 Hz, 1H, H-5″), 3.87–3.76 (m, 4H, H-4, H-5, H-5′, H-2″), 3.66 (app. t, *J*_1_ = *J*_2_ = 8.8 Hz, 1H, H-6), 3.61–3.37 (m, 8H, H-1, H-3, H-2′, H-4′, H-3″, H-4″, H-6″ (2H)), 3.28 (dd, *J*_1_ = 13.6 Hz, *J*_2_ = 3.6 Hz, 1H, H-6′), 3.19 (t, *J* = 7.6 Hz, 2H, SO_2_CH_2_CH_2_CH_2_(CH_2_)_8_CH_3_), 3.11 (dd, *J*_1_ = 13.6, *J*_2_ = 7.2 Hz, 1H, H-6′), 2.42 (dt, *J*_1_ = 12.4 Hz, *J*_2_ = *J*_3_ = 4.4 Hz, 1H, H-2eq), 2.16 (dt, *J*_1_ = 12.4 Hz, *J*_2_ = *J*_3_ = 4.4 Hz, 1H, H-3′eq), 1.86 (app. q, *J*_1_ = *J*_2_ = *J*_3_ = 12.8 Hz, 1H, H-3′ax), 1.78 (app. q, *J*_1_ = *J*_2_ = *J*_3_ = 12.8 Hz, 1H, H-2ax), 1.66 (p, *J* = 7.6 Hz, 2H, SO_2_CH_2_CH_2_CH_2_(CH_2_)_8_CH_3_), 1.30 (p, *J* = 7.6 Hz, 2H, SO_2_CH_2_CH_2_CH_2_(CH_2_)_8_CH_3_), 1.13 (m, 16H, SO_2_CH_2_CH_2_CH_2_(CH_2_)_8_CH_3_), 0.71 (t, *J* = 6.8 Hz, 3H, SO_2_CH_2_CH_2_CH_2_(CH_2_)_8_CH_3_).

*O*-3,6-Dideoxy-3-[[(1,1-dimethylethoxy)carbonyl]amino]-6-(4-decyl-1*H*-1,2,3-triazol-1-yl)-α-d-glucopyranosyl-(1→6)-*O*-[2,3,6-trideoxy-2,6-bis[[(1,1-dimethylethoxy)carbonyl]amino]-α-d-*ribo*-hexopyranosyl-(1→4)]-2-deoxy-*N*^1^,*N*^3^-bis[(1,1-dimethylethoxy)carbonyl]-d-streptamine (**6a**). To a microwave reaction vessel, compound **5** (60 mg, 0.060 mmol), sodium l-ascorbate (6 mg, 0.030 mmol), CuSO_4_·5H_2_O (6 mg, 0.024 mmol), 1-dodecyne (52 μL, 0.24 mmol), and DMF (1 mL) were added. The reaction vessel was then sealed and irradiated by microwaves for 4 min at 80 °C, twice. Progress of the reaction was monitored by TLC (Hexanes:EtOAc/3:7, R*_f_* 0.49). Upon completion, the reaction mixture was filtered through celite^®^, and eluted with EtOAc. The filtrate was washed with H_2_O (3×) and brine (3×), dried over anhydrous MgSO_4_, filtered, and concentrated to give a crude product. Purification by column chromatography (SiO_2_, pure Hexanes to Hexanes:EtOAc/2:3) gave compound **6a** (40 mg, 57%) as a white solid: ^1^H-NMR (400 MHz, CD_3_OD, Figure S4) δ 7.73 (s, 1H, triazole ring), 5.09 (d, *J* = 2.8 Hz, 1H, H-1″), 5.04 (br s, 1H, H-1′), 4.67 (br d, *J* = 11.2 Hz, 1H, H-6″), 4.55 (t, *J* = 12.0 Hz, 1H, H-6″), 4.39 (m, 1H, H-5″), 3.72 (t, *J* = 10.0 Hz, 1H, H-3″), 3.61–3.28 (m, 11H, H-1, H-3, H-4, H-5, H-6, H-2′, H-4′, H-5′, H-6′ (2H), H-2″), 2.96 (t, *J* = 10.0 Hz, 1H, H-4″), 2.66 (t, *J* = 7.6 Hz, 2H, CHN_3_CCH_2_CH_2_(CH_2_)_7_CH_3_), 2.01 (m, 2H, H-2eq, H-3′eq), 1.69–1.57 (m, 3H, H-3′ax, CHN_3_CCH_2_CH_2_(CH_2_)_7_CH_3_), 1.44–1.27 (m, 60H, H-2ax, 5× CO_2_(CH_3_)_3_, CHN_3_CCH_2_CH_2_(CH_2_)_7_CH_3_), 0.88 (t, *J* = 7.2 Hz, 3H, CHN_3_CCH_2_CH_2_(CH_2_)_7_CH_3_); ^13^C-NMR (100 MHz, CD_3_OD, Figure S5) δ 158.0, 157.8, 156.4, 156.3 (2 carbons), 147.6, 123.2, 98.4, 97.3, 81.6, 80.3, 79.3, 79.0 (2 carbons), 78.9, 78.7, 75.1, 72.1, 70.4, 70.2, 69.0, 65.0, 55.6, 50.3, 50.2, 49.8, 40.6, 34.6 (2 carbons), 32.9, 31.6, 29.32, 29.30, 29.2, 29.1, 29.0, 28.9, 27.4 (15 carbons), 24.9, 22.3, 13.0; HR-MS (Figure S6) calcd for C_55_H_99_N_8_O_18_ [M + H]^+^ 1159.7072; found 1159.7096.

*O*-3,6-Dideoxy-3-[[(1,1-dimethylethoxy)carbonyl]amino]-6-(4-dodecyl-1*H*-1,2,3-triazol-1-yl)-α-d-glucopyranosyl-(1→6)-*O*-[2,3,6-trideoxy-2,6-bis[[(1,1-dimethylethoxy)carbonyl]amino]-α-d-*ribo*-hexopyranosyl-(1→4)]-2-deoxy-*N*^1^,*N*^3^-bis[(1,1-dimethylethoxy)carbonyl]-d-streptamine (**6b**). To a microwave reaction vessel, compound **5** (60 mg, 0.060 mmol), sodium l-ascorbate (6 mg, 0.030 mmol), CuSO_4_·5H_2_O (6 mg, 0.024 mmol), 1-tetradecyne (66 μL, 0.24 mmol), and DMF (1 mL) were added. The reaction vessel was then sealed and irradiated by microwaves for 4 min at 80 °C, twice. Progress of the reaction was monitored by TLC (Hexanes:EtOAc/3:7, R*_f_* 0.58). Upon completion, the reaction mixture was filtered through celite^®^, and eluted with EtOAc. The filtrate was washed with H_2_O (3×) and brine (3×), dried over anhydrous MgSO_4_, filtered, and concentrated to give a crude product. Purification by column chromatography (SiO_2_, pure Hexanes to Hexanes:EtOAc/2:3) gave the known compound **6b** [11] (45 mg, 63%) as a white solid: ^1^H-NMR (400 MHz, CD_3_OD, which matches the Reference [11], Figure S7) δ 7.73 (s, 1H, triazole ring), 5.09 (br s, 1H, H-1″), 5.03 (br s, 1H, H-1′), 4.66 (br d, *J* = 10.4 Hz, 1H, H-6″), 4.55 (t, *J* = 14.4 Hz, 1H, H-6″), 4.40 (m, 1H, H-5″), 3.72 (t, *J* = 10.8 Hz, 1H, H-3″), 3.61–3.24 (m, 11H, H-1, H-3, H-4, H-5, H-6, H-2′, H-4′, H-5′, H-6′ (2H), H-2″), 2.96 (t, *J* = 10.8 Hz, 1H, H-4″), 2.66 (t, *J* = 7.2 Hz, 2H, CHN_3_CCH_2_CH_2_(CH_2_)_9_CH_3_), 2.01 (m, 2H, H-2eq, H-3′eq), 1.69–1.57 (m, 3H, H-3′ax, CHN_3_CCH_2_CH_2_(CH_2_)_9_CH_3_), 1.44–1.27 (m, 64H, H-2ax, 5× CO_2_(CH_3_)_3_, CHN_3_CCH_2_CH_2_(CH_2_)_9_CH_3_), 0.88 (t, *J* = 7.6 Hz, 3H, CHN_3_CCH_2_CH_2_(CH_2_)_9_CH_3_).

*O*-3-Amino-3,6-dideoxy-6-(4-decyl-1*H*-1,2,3-triazol-1-yl)-α-d-glucopyranosyl-(1→6)-*O*-[2,6-diamino-2,3,6-trideoxy-α-d-*ribo*-hexopyranosyl-(1→4)]-2-deoxy-d-streptamine (**7a**). Compound **6a** (30 mg, 0.026 mmol) was treated at room temperature with neat TFA (1 mL) for 3 min. The TFA was removed under reduced pressure, the residue was dissolved in a minimal volume of H_2_O, extracted with CH_2_Cl_2_ (3×), and freeze-dried to afford compound **7a** (30 mg, 94%) as a white powder: ^1^H-NMR (400 MHz, D_2_O, Figure S8) δ 7.62 (s, 1H, triazole ring), 5.64 (d, *J* = 3.6 Hz, 1H, H-1′), 4.88 (d, *J* = 3.6 Hz, 1H, H-1″), 4.58 (m, 1H, H-6″), 4.44 (dd, *J*_1_ = 14.8 Hz, *J*_2_ = 7.2 Hz, 1H, H-6″), 4.18 (m, 1H, H-5″), 3.81–3.68 (m, 3H, H-4, H-5′, H-2″), 3.64–3.50 (m, 4H, H-5, H-6, H-2′, H-4′), 3.44–3.30 (m, 4H, H-1, H-3, H-3″, H-4″), 3.27 (dd, *J*_1_ = 13.6 Hz, *J*_2_ = 3.6 Hz, 1H, H-6′), 3.07 (dd, *J*_1_ = 14.0 Hz, *J*_2_ = 7.6 Hz, 1H, H-6′), 2.51 (t, *J* = 7.2 Hz, 2H, CHN_3_CCH_2_CH_2_(CH_2_)_7_CH_3_), 2.36 (dt, *J*_1_ = 12.8 Hz, *J*_2_ = *J*_3_ = 3.6 Hz, 1H, H-2eq), 2.16 (dt, *J*_1_ = 12.4 Hz, *J*_2_ = *J*_3_ = 4.4 Hz, 1H, H-3′eq), 1.87 (app. q, *J*_1_ = *J*_2_ = *J*_3_ = 12.0 Hz, 1H, H-3′ax), 1.74 (app. q, *J*_1_ = *J*_2_ = *J*_3_ = 12.8 Hz, 1H, H-2ax), 1.45 (p, *J* = 6.4 Hz, 2H, CHN_3_CCH_2_CH_2_(CH_2_)_7_CH_3_) 1.20–1.00 (m, 14H, CHN_3_CCH_2_CH_2_(CH_2_)_7_CH_3_), 0.67 (t, *J* = 6.8 Hz, 3H, CHN_3_CCH_2_CH_2_(CH_2_)_7_CH_3_); ^13^C-NMR (100 MHz, D_2_O, Figure S9) δ 148.6, 125.0, 100.8, 93.8, 83.9, 76.6, 74.2, 70.8, 69.9, 67.7, 66.5, 64.4, 54.6, 50.2, 49.5, 48.2, 47.7, 39.8, 31.1, 29.3, 28.54, 28.47, 28.4, 28.3, 28.2, 27.9, 27.6, 24.3, 21.9, 13.3; HR-MS (Figure S10) calcd for C_30_H_59_N_8_O_8_ [M + H]^+^ 659.4450; found 659.4457.

*O*-3-Amino-3,6-dideoxy-6-(4-dodecyl-1*H*-1,2,3-triazol-1-yl)-α-d-glucopyranosyl-(1→6)-*O*-[2,6-diamino-2,3,6-trideoxy-α-d-*ribo*-hexopyranosyl-(1→4)]-2-deoxy-d-streptamine (**7b**). Compound **6b** (35 mg, 0.029 mmol) was treated at room temperature with neat TFA (1 mL) for 3 min. The TFA was removed under reduced pressure, the residue was dissolved in a minimal volume of H_2_O, extracted with CH_2_Cl_2_ (3×), and freeze-dried to afford the known compound **7b** [11] (38 mg, quant.) as a white powder: ^1^H-NMR (400 MHz, D_2_O, which matches Reference [11], Figure S11) δ 7.62 (s, 1H, triazole ring), 5.64 (d, *J* = 3.6 Hz, 1H, H-1′), 4.88 (d, *J* = 3.6 Hz, 1H, H-1″), 4.63 (m, 1H, H-6″), 4.44 (dd, *J*_1_ = 14.8 Hz, *J*_2_ = 7.2 Hz, 1H, H-6″), 4.18 (m, 1H, H-5″), 3.80–3.69 (m, 3H, H-4, H-5′, H-2″), 3.64–3.50 (m, 4H, H-5, H-6, H-2′, H-4′), 3.42–3.30 (m, 4H, H-1, H-3, H-3″, H-4″), 3.27 (dd, *J*_1_ = 13.6 Hz, *J*_2_ = 2.8 Hz, 1H, H-6′), 3.07 (dd, *J*_1_ = 13.6 Hz, *J*_2_ = 7.6 Hz, 1H, H-6′), 2.51 (t, *J* = 7.2 Hz, 2H, CHN_3_CCH_2_CH_2_(CH_2_)_9_CH_3_), 2.36 (dt, *J*_1_ = 12.8 Hz, *J*_2_ = *J*_3_ = 4.4 Hz, 1H, H-2eq), 2.16 (dt, *J*_1_ = 12.4 Hz, *J*_2_ = *J*_3_ = 4.4 Hz, 1H, H-3′eq), 1.87 (app. q, *J*_1_ = *J*_2_ = *J*_3_ = 12.0 Hz, 1H, H-3′ax), 1.74 (app. q, *J*_1_ = *J*_2_ = *J*_3_ = 12.4 Hz, 1H, H-2ax), 1.45 (p, *J* = 6.4 Hz, 2H, CHN_3_CCH_2_CH_2_(CH_2_)_9_CH_3_) 1.20–1.00 (m, 18H, CHN_3_CCH_2_CH_2_(CH_2_)_9_CH_3_), 0.67 (t, *J* = 6.8 Hz, 3H, CHN_3_CCH_2_CH_2_(CH_2_)_9_CH_3_).

*O*-3,6-Dideoxy-3-[[(1,1-dimethylethoxy)carbonyl]amino]-6-[(1-oxododecyl)amino]-α-d-glucopyranosyl-(1→6)-*O*-[2,3,6-trideoxy-2,6-bis[[(1,1-dimethylethoxy)carbonyl]amino]-α-d-*ribo*-hexopyranosyl-(1→4)]-2-deoxy-*N*^1^,*N*^3^-bis[(1,1-dimethylethoxy)carbonyl]-d-streptamine (**8**). A solution of compound **7** (80 mg, 0.081 mmol) in THF (2 mL) and H_2_O (0.1 mL) was treated with 1 M PMe_3_ in THF (0.11 mL, 0.11 mmol) and the resulting mixture was stirred at 50 °C for 1 h. Progress of the reaction was monitored by TLC (CH_2_Cl_2_:MeOH/9:1 + NH_4_OH (0.7%), R*_f_* 0.25). Upon completion, the solvents were removed and the crude material obtained was filtered through silica gel, eluting with Hexanes:EtOAc/1:3 and CH_2_Cl_2_:MeOH/9:1 + NH_4_OH (0.7%). The filtrate collected from the latter eluting solvent was concentrated, redissolved in anhydrous DMF, and added to a pre-stirred, ice-cooled solution of lauric acid (32 mg, 0.16 mmol), EDC·HCl (39 mg, 0.20 mmol), HOBt (31 mg, 0.20 mmol), DIPEA (0.11 mL, 0.64 mmol), and anhydrous DMF (2 mL). The resulting mixture was stirred overnight till room temperature. Progress of the reaction was monitored by TLC (Hexanes:EtOAc/2:3, R*_f_* 0.24). Upon completion, the reaction mixture was diluted with H_2_O and extracted with EtOAc (3×). The combined organic layers were washed with H_2_O (3×) and brine (3×), dried over anhydrous MgSO_4_, filtered, and concentrated to give a crude product. Purification by flash column chromatography (SiO_2_, pure Hexanes to Hexanes:EtOAc/2:3) gave compound **8** (51 mg, 55%) as a white solid: ^1^H-NMR (400 MHz, CD_3_OD, Figure S12) δ 5.11 (br s, 1H, H-1′), 5.01 (d, *J* = 3.2 Hz, 1H, H-1″), 4.01 (m, 1H, H-5″), 3.66–3.28 (m, 14H, H-1, H-3, H-4, H-5, H-6, H-2′, H-4′, H-5′, H-6′ (2H), H-2″, H-3″, H-6″ (2H)), 3.14 (t, *J* = 9.6 Hz, 1H, H-4″), 2.19 (t, *J* = 7.2 Hz, 2H, NHCOCH_2_(CH_2_)_9_CH_3_), 2.10–1.90 (m, 2H, H-2eq, H-3′eq), 1.70–1.20 (m, 65H, H-2ax, H-3′ax, 5× CO_2_(CH_3_)_3_, NHCOCH_2_(CH_2_)_9_CH_3_), 0.88 (t, *J* = 7.2 Hz, 3H, NHCOCH_2_(CH_2_)_9_CH_3_); ^13^C-NMR (100 MHz, CD_3_OD, Figure S13) δ 175.6, 157.9, 157.8, 156.6, 156.33, 156.25, 98.4, 98.0, 81.8, 81.1, 79.3, 79.1, 78.9 (2 carbons), 78.7, 75.5, 72.0, 71.2, 70.7, 69.3, 65.1, 55.6, 50.3, 49.7, 49.5, 40.6, 40.1, 35.7, 34.4, 32.9, 31.7, 29.4, 29.34, 29.26, 29.1, 29.0, 28.4, 27.4 (15 carbons), 25.6, 22.3, 13.0; HR-MS (Figure S14) calcd for C_55_H_101_N_6_O_19_ [M + H]^+^ 1149.7116; found 1149.7128.

*O*-3-Amino-3,6-dideoxy-6-[(1-oxododecyl)amino]-α-d-glucopyranosyl-(1→6)-*O*-[2,6-diamino-2,3,6-trideoxy-α-d-*ribo*-hexopyranosyl-(1→4)]-2-deoxy-d-streptamine (**9**). Compound **8** (41 mg, 0.036 mmol) was treated at room temperature with neat TFA (1 mL) for 3 min. The TFA was removed under reduced pressure, the residue was dissolved in a minimal volume of H_2_O, extracted with CH_2_Cl_2_ (3×), and freeze-dried to afford compound **9** (40 mg, 93%) as a white powder: ^1^H-NMR (400 MHz, D_2_O, Figure S15) δ 5.63 (d, *J* = 3.6 Hz, 1H, H-1′), 4.88 (d, *J* = 4.0 Hz, 1H, H-1″), 3.84–3.71 (m, 4H, H-4, H-5′, H-2″, H-5″), 3.66 (t, *J* = 9.6 Hz, 1H, H-5), 3.61–3.23 (m, 10H, H-1, H-3, H-6, H-2′, H-4′, H-6′, H-3″, H-4″, H-6″ (2H)), 3.09 (dd, *J*_1_ = 13.6 Hz, *J*_2_ = 6.8 Hz, 1H, H-6′), 2.37 (dt, *J*_1_ = 12.4 Hz, *J*_2_ = *J*_3_ = 4.4 Hz, 1H, H-2eq), 2.13 (m, 1H, H-3′eq), 2.09 (t, *J* = 8.0 Hz, 2H, NHCOCH_2_CH_2_(CH_2_)_8_CH_3_), 1.85 (app. q, *J*_1_ = *J*_2_ = *J*_3_ = 12.0 Hz, 1H, H-3′ax), 1.76 (app. q, *J*_1_ = *J*_2_ = *J*_3_ = 12.8 Hz, 1H, H-2ax), 1.40 (p, *J* = 6.0 Hz, 2H, NHCOCH_2_CH_2_(CH_2_)_8_CH_3_), 1.09 (m, 16H, NHCOCH_2_CH_2_(CH_2_)_8_CH_3_), 0.67 (t, *J* = 6.8 Hz, 3H, NHCOCH_2_CH_2_(CH_2_)_8_CH_3_); ^13^C-NMR (100 MHz, D_2_O, Figure S16) δ 178.1, 100.8, 94.0, 83.5, 77.2, 74.1, 70.9, 70.2, 68.0, 66.4, 64.3, 54.5, 49.6, 48.2, 47.6, 39.7, 38.8, 35.7, 31.1, 29.2, 28.6 (2 carbons), 28.5, 28.4, 28.24, 28.16, 27.7, 25.3, 21.9, 13.3; HR-MS (Figure S17) calcd for C_30_H_60_N_6_O_9_ [M + H]^+^ 649.4495; found 649.4494.

*O*-3-Azido-3-deoxy-α-d-glucopyranosyl-(1→6)-*O*-[2,6-diazido-2,3,6-trideoxy-α-d-*ribo*-hexopyranosyl-(1→4)]-1,3-diazido-1,2,3-trideoxy-d-*myo*-inositol (**10**). The synthesis started with the preparation of triflic azide (TfN_3_). Note: extreme caution should be exercised while handling azides! A solution of NaN_3_ (8.34 g, 128.3 mmol) in H_2_O (25 mL) was cooled down to 0 °C in an ice-H_2_O bath and vigorously stirred. CH_2_Cl_2_ (25 mL) was added, followed by Tf_2_O (10.8 mL, 64.2 mmol), and the resulting mixture was stirred for 2 h at 0 °C. 30 min before completion of the TfN_3_ reaction, tobramycin (2.0 g, 4.28 mmol) and ZnCl_2_ (29 mg, 0.21 mmol) were dissolved in H_2_O (65 mL). Et_3_N (8.9 mL, 64.2 mmol) was added, followed by dropwise addition of MeOH (215 mL). The resulting mixture was stirred at room temperature. Meanwhile, saturated aqueous NaHCO_3_ was carefully added to the stirred solution of TfN_3_ until the reaction mixture stopped fizzing. The TfN_3_ mixture was then extracted with CH_2_Cl_2_ (2 × 20 mL), and the combined CH_2_Cl_2_ solutions of TfN_3_ were washed again with saturated NaHCO_3_, and filtered directly into the tobramycin solution. The resulting mixture was vigorously stirred for 3 h. The reaction was quenched by addition of solid NaHCO_3_, filtered through a bed of celite^®^, and the filtrate was dried in vacuo. Purification by flash column chromatography (SiO_2_, pure CH_2_Cl_2_ to CH_2_Cl_2_:MeOH/19:1, R*_f_* 0.29 in CH_2_Cl_2_:MeOH/19:1) gave the known compound **10** [45] (2.4 g, 92%) as a white solid: ^1^H-NMR (400 MHz, CD_3_OD, which matches Reference [45], Figure S18) δ 5.58 (d, *J* = 3.2 Hz, 1H, H-1′), 5.23 (d, *J* = 3.6 Hz, 1H, H-1″), 4.08 (ddd, *J*_1_ = 9.2 Hz, *J*_2_ = 5.6 Hz, *J*_3_ = 2.4 Hz, 1H), 4.02 (ddd, *J*_1_ = 9.6 Hz, *J*_2_ = 4.4 Hz, *J*_3_ = 2.4 Hz, 1H), 3.76 (dd, *J*_1_ = 12.0 Hz, *J*_2_ = 2.4 Hz, 1H), 3.68–3.29 (m, 10H), 3.21–3.15 (m, 3H), 2.37 (dt, *J*_1_ = 12.4 Hz, *J*_2_ = *J*_3_ = 4.4 Hz, 1H, H-2eq), 2.13 (dt, *J*_1_ = 11.2 Hz, *J*_2_ = *J*_3_ = 4.4 Hz, 1H, H-3′eq), 1.99 (app. q, *J*_1_ = *J*_2_ = *J*_3_ = 11.2 Hz, 1H, H-3′ax), 1.55 (app. q, *J*_1_ = *J*_2_ = *J*_3_ = 12.0 Hz, 1H, H-2ax).

*O*-3-Azido-3-deoxy-6-*O*-pivaloyl-α-d-glucopyranosyl-(1→6)-*O*-[2,6-diazido-2,3,6-trideoxy-α-d-*ribo*-hexopyranosyl-(1→4)]-1,3-diazido-1,2,3-trideoxy-d-*myo*-inositol (**11**). A solution of compound **10** (500 mg, 0.84 mmol) in pyridine (3 mL) at 0 °C was treated with trimethylacetyl chloride (0.12 mL, 1.01 mmol) and the resulting mixture was stirred at 0 °C for 4 h. Progress of the reaction was monitored by TLC (Hexanes:EtOAc/2:3, R*_f_* 0.38). Upon completion, the reaction was quenched with H_2_O and extracted with EtOAc (2×). The combined organic layers were washed with H_2_O and brine, dried over anhydrous MgSO_4_, filtered, and concentrated to give a crude product. Purification by flash column chromatography (SiO_2_, Hexanes:EtOAc/3:2) gave compound **11** (360 mg, 63%) as a colorless liquid: ^1^H-NMR (400 MHz, CD_3_OD, Figure S19) δ 5.60 (d, *J* = 3.6 Hz, 1H, H-1′), 5.26 (d, *J* = 4.0 Hz, 1H, H-1″), 4.35 (dd, *J*_1_ = 11.6 Hz, *J*_2_ = 2.0 Hz, 1H), 4.29 (ddd, *J*_1_ = 10.0 Hz, *J*_2_ = 4.4 Hz, *J*_3_ = 2.0 Hz, 1H), 4.12 (dd, *J*_1_ = 12.0 Hz, *J*_2_ = 4.8 Hz, 1H), 4.08 (ddd, *J*_1_ = 10.0 Hz, *J*_2_ = 5.6 Hz, *J*_3_ = 2.0 Hz, 1H), 3.69–3.62 (m, 3H), 3.61 (dd, *J*_1_ = 11.2 Hz, *J*_2_ = 4.0 Hz, 1H), 3.55 (ddd, *J*_1_ = 11.2 Hz, *J*_2_ = 4.8 Hz, *J*_3_ = 2.0 Hz, 1H), 3.51 (dd, *J*_1_ = 8.0 Hz, *J*_2_ = 2.0 Hz, 1H), 3.50 (m, 1H), 3.48 (dd, *J*_1_ = 13.6 Hz, *J*_2_ = 2.8 Hz, 1H), 3.40 (dd, *J*_1_ = 10.4 Hz, *J*_2_ = 3.6 Hz, 1H), 3.39–3.31 (m, 2H), 3.20 (dt, *J*_1_ = 12.8 Hz, *J*_2_ = *J*_3_ = 4.0 Hz, 1H), 2.38 (dt, *J*_1_ = 12.8 Hz, *J*_2_ = *J*_3_ = 4.0 Hz, 1H, H-2eq), 2.14 (dt, *J*_1_ = 11.2 Hz, *J*_2_ = *J*_3_ = 4.8 Hz, 1H, H-3′eq), 1.99 (app. q, *J*_1_ = *J*_2_ = *J*_3_ = 12.4 Hz, 1H, H-3′ax), 1.54 (app. q, *J*_1_ = *J*_2_ = *J*_3_ = 12.4 Hz, 1H, H-2ax), 1.18 (s, 9H); ^13^C-NMR (100 MHz, CD_3_OD, Figure S20) δ 178.6, 97.9, 97.0, 80.2, 79.2, 74.9, 72.7, 70.8, 70.0, 68.7, 66.7, 65.1, 62.9, 60.5, 59.3, 56.4, 51.1, 38.6, 32.0, 30.8, 26.2 (3 carbons); HR-MS (Figure S21) calcd for C_23_H_35_N_15_O_10_Na [M + Na]^+^ 704.2589; found 704.2589.

*O*-3-Azido-3-deoxy-2,4-bis-*O*-(phenylmethyl)-6-*O*-pivaloyl-α-d-glucopyranosyl-(1→6)-*O*-[2,6-diazido-2,3,6-trideoxy-4-*O*-(phenylmethyl)-α-d-*ribo*-hexopyranosyl-(1→4)]-1,3-diazido-1,2,3-trideoxy-5-*O*-(phenylmethyl)-d-*myo*-inositol (**12**). A solution of compound **11** (355 mg, 0.52 mmol) in DMF (3 mL) at 0 °C was treated with 60% sodium hydride (125 mg, 3.13 mmol). The reaction mixture was stirred at 0 °C for 15 min followed by the addition of benzyl bromide (0.37 mL, 3.13 mmol) and TBAI (96 mg, 0.26 mmol). The reaction mixture was allowed to warm to room temperature and stirred overnight. Progress of the reaction was monitored by TLC (Hexanes:EtOAc/5:1, R*_f_* 0.44). The reaction mixture was quenched with H_2_O and extracted with EtOAc (2×). The combined organic layers were washed with H_2_O and brine, dried over anhydrous MgSO_4_, filtered, and concentrated to give a crude product. Purification by flash column chromatography (SiO_2_, pure Hexanes to Hexanes:EtOAc/4:1) gave compound **12** (410 mg, 76%) as a white foam: ^1^H-NMR (400 MHz, CDCl_3_, Figure S22) δ 7.43–7.25 (m, 13H, aromatic), 7.20 (dd, *J*_1_ = 7.6 Hz, *J*_2_ = 4.0 Hz, 2H, aromatic), 7.10–7.06 (m, 3H, aromatic), 7.04 (dd, *J*_1_ = 7.6 Hz, *J*_2_ = 3.6 Hz, 2H, aromatic), 5.64 (d, *J* = 3.6 Hz, 1H, H-1′), 5.43 (d, *J* = 4.0 Hz, 1H, H-1″), 4.96 (d, *J* = 12.0 Hz, 1H), 4.92 (d, *J* = 12.0 Hz, 1H), 4.81 (d, *J* = 11.6 Hz, 1H), 4.73 (d, *J* = 11.6 Hz, 1H), 4.65 (d, *J* = 10.8 Hz, 1H), 4.63 (d, *J* = 11.2 Hz, 1H), 4.44 (d, *J* = 11.2 Hz, 1H), 4.28 (d, *J* = 11.2 Hz, 1H), 4.19 (ddd, *J*_1_ = 9.6 Hz, *J*_2_ = 4.4 Hz, *J*_3_ = 2.0 Hz, 1H), 4.03 (dd, *J*_1_ = 12.0 Hz, *J*_2_ = 2.4 Hz, 1H), 3.92 (dd, *J*_1_ = 10.0 Hz, *J*_2_ = 2.4 Hz, 1H), 3.80 (app. t, *J*_1_ = *J*_2_ = 10.0 Hz, 1H), 3.72–3.50 (m, 6H), 3.48–3.38 (m, 3H), 3.31 (dd, *J*_1_ = 10.8 Hz, *J*_2_ = 4.0 Hz, 1H), 3.10 (app. t, *J*_1_ = *J*_2_ = 9.6 Hz, 1H), 2.99 (dt, *J*_1_ = 13.2 Hz, *J*_2_ = *J*_3_ = 4.0 Hz, 1H), 2.39 (dt, *J*_1_ = 13.6 Hz, *J*_2_ = *J*_3_ = 4.4 Hz, 1H, H-2eq), 2.31 (dt, *J*_1_ = 11.6 Hz, *J*_2_ = *J*_3_ = 4.4 Hz, 1H, H-3′eq), 1.99 (app. q, *J*_1_ = *J*_2_ = *J*_3_ = 12.4 Hz, 1H, H-3′ax), 1.62 (app. q, *J*_1_ = *J*_2_ = *J*_3_ = 12.8 Hz, 1H, H-2ax), 1.16 (s, 9H); ^13^C-NMR (100 MHz, CDCl_3_, Figure S23) δ 177.9, 137.44, 137.40, 137.2, 137.1, 128.54 (2 carbons), 128.52 (2 carbons), 128.3 (4 carbons), 128.12, 128.09 (2 carbons), 128.0, 127.9 (2 carbons), 127.8 (2 carbons), 127.7, 127.3, 125.9 (2 carbons), 96.3, 95.1, 83.3, 77.8, 77.2, 77.1, 76.4, 75.0, 74.7, 73.1, 71.8, 71.0, 70.8, 68.7, 65.1, 62.3, 60.2, 59.4, 56.2, 51.2, 38.8, 31.9, 27.8, 27.2 (3 carbons); HR-MS (Figure S24) calcd for C_51_H_59_N_15_O_10_Na [M + Na]^+^ 1064.4467; found 1064.4483.

*O*-3-Azido-3-deoxy-2,4-bis-*O*-(phenylmethyl)-α-d-glucopyranosyl-(1→6)-*O*-[2,6-diazido-2,3,6-trideoxy-4-*O*-(phenylmethyl)-α-d-*ribo*-hexopyranosyl-(1→4)]-1,3-diazido-1,2,3-trideoxy-5-*O*-(phenylmethyl)-d-*myo*-inositol (**13**). A solution of compound **12** (395 mg, 0.38 mmol) was dissolved in MeOH (5 mL), and sodium metal (3.3 mg, 0.14 mmol) was added. The resulting mixture was stirred at 60 °C for 6 h and progress of the reaction was monitored by TLC (Hexanes:EtOAc/4:1, R*_f_* 0.20). The reaction was quenched with H_2_O and MeOH was removed. The resulting mixture was extracted with EtOAc (2×). The combined organic layers were washed with H_2_O and brine, dried over anhydrous MgSO_4_, filtered, and concentrated to give a crude product. Purification by flash column chromatography (SiO_2_, Hexanes:EtOAc/4:1) gave compound **13** (294 mg, 81%) as a white solid: ^1^H-NMR (400 MHz, CDCl_3_, Figure S25) δ 7.43–7.26 (m, 13H, aromatic), 7.20 (dd, *J*_1_ = 6.8 Hz, *J*_2_ = 2.8 Hz, 2H, aromatic), 7.13–7.08 (m, 5H, aromatic), 5.58 (d, *J* = 4.0 Hz, 1H, H-1′), 5.42 (d, *J* = 3.6 Hz, 1H, H-1″), 4.94 (d, *J* = 11.6 Hz, 1H), 4.90 (d, *J* = 11.6 Hz, 1H), 4.80 (d, *J* = 12.0 Hz, 1H), 4.74 (d, *J* = 11.6 Hz, 1H), 4.68 (d, *J* = 11.6 Hz, 1H), 4.63 (d, *J* = 11.2 Hz, 1H), 4.44 (d, *J* = 11.2 Hz, 1H), 4.41 (d, *J* = 11.2 Hz, 1H), 4.19 (ddd, *J*_1_ = 9.6 Hz, *J*_2_ = 4.8 Hz, *J*_3_ = 2.4 Hz, 1H), 3.81 (app. t, *J*_1_ = *J*_2_ = 10.0 Hz, 1H), 3.74–3.66 (m, 2H), 3.62 (app. t, *J*_1_ = *J*_2_ = 9.2 Hz, 1H), 3.58–3.50 (m, 3H), 3.48–3.37 (m, 4H), 3.31 (dd, *J*_1_ = 10.8 Hz, *J*_2_ = 3.6 Hz, 1H), 3.22 (app. t, *J*_1_ = *J*_2_ = 10.0 Hz, 1H), 3.17 (m, 1H), 2.99 (dt, *J*_1_ = 12.8 Hz, *J*_2_ = *J*_3_ = 4.0 Hz, 1H), 2.38 (dt, *J*_1_ = 12.8 Hz, *J*_2_ = *J*_3_ = 4.8 Hz, 1H, H-2eq), 2.32 (dt, *J*_1_ = 11.2 Hz, *J*_2_ = *J*_3_ = 4.4 Hz, 1H, H-3′eq), 1.99 (app. q, *J*_1_ = *J*_2_ = *J*_3_ = 12.4 Hz, 1H, H-3′ax), 1.63 (app. q, *J*_1_ = *J*_2_ = *J*_3_ = 12.8 Hz, 1H, H-2ax); ^13^C-NMR (100 MHz, CD_3_OD, Figure S26) δ 138.1, 138.0, 137.8, 137.6, 128.1 (2 carbons), 128.0 (2 carbons), 127.9 (2 carbons), 127.82 (2 carbons), 127.78 (2 carbons), 127.62, 127.55 (2 carbons), 127.5 (2 carbons), 127.4, 127.1, 126.7, 125.9 (2 carbons), 95.9, 95.0, 83.1, 78.0, 77.3, 77.0, 75.7, 74.3, 74.1, 72.6, 72.2, 71.2, 70.7, 70.4, 65.3, 60.2, 59.8, 59.7, 56.1, 51.0, 31.4, 27.3; HR-MS (Figure S27) calcd for C_46_H_51_N_15_O_9_Na [M + Na]^+^ 980.3892; found 980.3896.

*O*-3-Azido-3-deoxy-6-*O*-dodecyl-2,4-bis-*O*-(phenylmethyl)-α-d-glucopyranosyl-(1→6)-*O*-[2,6-diazido-2,3,6-trideoxy-4-*O*-(phenylmethyl)-α-d-*ribo*-hexopyranosyl-(1→4)]-1,3-diazido-1,2,3-trideoxy-5-*O*-(phenylmethyl)-d-*myo*-inositol (**14**). A solution of compound **13** (80 mg, 0.08 mmol) in THF (1.5 mL) at 0 °C was treated with 60% sodium hydride (20 mg, 0.84 mmol). The reaction mixture was stirred at 0 °C for 15 min followed by the addition of 1-bromododecane (0.20 mL, 0.84 mmol), and a catalytic amount of TBAI (3 mg). The reaction mixture was allowed to warm to room temperature and stirred for 12 h. Progress of the reaction was monitored by TLC (Hexanes:EtOAc/4:1, R*_f_* 0.65). The reaction was quenched with H_2_O and extracted with EtOAc (2×). The combined organic layers were washed with H_2_O and brine, dried over anhydrous MgSO_4_, filtered, and concentrated to give a crude product. Purification by flash column chromatography (SiO_2_, Hexanes:EtOAc/4:1) gave compound **14** (77 mg, 82%) as a white solid: ^1^H-NMR (400 MHz, CD_3_OD, Figure S28) δ 7.43 (d, *J* = 7.6 Hz, 2H, aromatic), 7.35 (t, *J* = 7.6 Hz, 2H, aromatic), 7.31–7.23 (m, 11H), 7.12–7.09 (m, 5H, aromatic), 5.68 (d, *J* = 3.6 Hz, 1H, H-1′), 5.44 (d, *J* = 3.6 Hz, 1H, H-1″), 4.97 (d, *J* = 11.6 Hz, 1H), 4.92 (d, *J* = 11.6 Hz, 1H), 4.81 (d, *J* = 11.6 Hz, 1H), 4.71 (d, *J* = 11.6 Hz, 1H), 4.64 (d, *J* = 12.0 Hz, 1H), 4.63 (d, *J* = 11.2 Hz, 1H), 4.46 (d, *J* = 11.6 Hz, 1H), 4.40 (d, *J* = 10.8 Hz, 1H), 4.19 (m, 1H), 3.85 (m, 1H), 3.79–3.57 (m, 7H), 3.52–3.44 (m, 2H), 3.39 (dd, *J*_1_ = 10.4 Hz, *J*_2_ = 3.2 Hz, 1H), 3.36 (dd, *J*_1_ = 13.6 Hz, *J*_2_ = 5.6 Hz, 1H), 3.24 (t, *J* = 10.0 Hz, 2H, OCH_2_CH_2_CH_2_(CH_2_)_8_CH_3_), 3.18–3.05 (m, 3H), 2.38 (dt, *J*_1_ = 12.0 Hz, *J*_2_ = *J*_3_ = 4.4 Hz, 2H, H-2eq, H-3′eq), 1.95 (app. q, *J*_1_ = *J*_2_ = *J*_3_ = 11.6 Hz, 1H, H-3′ax), 1.85 (p, *J* = 3.2 Hz, 2H, OCH_2_CH_2_CH_2_(CH_2_)_8_CH_3_), 1.57 (app. q, *J*_1_ = *J*_2_ = *J*_3_ = 12.4 Hz, 1H, H-2ax), 1.43 (p, *J* = 6.8 Hz, 2H, OCH_2_CH_2_CH_2_(CH_2_)_8_CH_3_), 1.25 (m, 16H, OCH_2_CH_2_CH_2_(CH_2_)_8_CH_3_), 0.87 (t, *J* = 6.0 Hz, 3H, OCH_2_CH_2_CH_2_(CH_2_)_8_CH_3_); ^13^C-NMR (100 MHz, CDCl_3_, Figure S29) δ 138.0, 137.4, 137.3 (2 carbons), 128.50 (2 carbons), 128.48 (2 carbons), 128.18 (2 carbons), 128.16 (2 carbons), 128.15 (2 carbons), 128.05, 128.02, 127.8 (2 carbons), 127.7 (2 carbons), 127.5, 127.1, 126.2 (2 carbons), 96.3, 95.7, 83.2, 77.6, 77.1, 75.9, 74.9, 74.5, 73.0, 71.8, 71.6, 70.9, 70.8, 70.1, 68.3, 68.0, 65.3, 60.2, 59.4, 56.1, 51.2, 31.9, 29.69, 29.65, 29.62 (2 carbons), 29.57, 29.4, 29.3, 27.8, 26.1, 25.6, 22.7, 14.1; HR-MS (Figure S30) calcd for C_58_H_75_N_15_O_19_Na [M + Na]^+^ 1148.5770; found 1148.5786.

*O*-3-Deoxy-3-[[(1,1-dimethylethoxy)carbonyl]amino]-6-*O*-dodecyl-α-d-glucopyranosyl-(1→6)-*O*-[2,3,6-trideoxy-2,6-bis[[(1,1-dimethylethoxy)carbonyl]amino]-α-d-*ribo*-hexopyranosyl-(1→4)]-2-deoxy-*N*^1^,*N*^3^-bis[(1,1-dimethylethoxy)carbonyl]-d-streptamine (**15**). A solution of compound **14** (95 mg, 0.084 mmol) in THF (2 mL) and 0.1 M NaOH (0.5 mL) was treated with 1 M PMe_3_ in THF (0.59 mL, 0.59 mmol) and the resulting mixture was stirred at 50 °C for 2 h. Upon completion, the solvents were removed and the crude material obtained was dissolved in 5 mL of degassed AcOH:H_2_O/1:3. A catalytic amount of Pd(OH)_2_/C was added and the resulting mixture was stirred at room temperature overnight under H_2_ atmosphere. The following day, the mixture was filtered through celite^®^, and the residue was washed with H_2_O. The filtrate was freeze-dried overnight. The residue obtained was dissolved in THF (5 mL) and treated with Boc_2_O (110 mg, 0.51 mmol) and Et_3_N (0.14 mL, 1.01 mmol). The resulting mixture was stirred at 60 °C for 6 h. Progress of the reaction was monitored by TLC (Hexanes:EtOAc/2:3, R*_f_* 0.34). The solvents were then removed and the crude material obtained was purified by column chromatography (SiO_2_, pure Hexanes to Hexanes:EtOAc/3:7) to give compound **15** (31 mg, 32% over 3 steps) as a white solid: ^1^H-NMR (400 MHz, CD_3_OD, Figure S31) δ 5.10 (br s, 1H, H-1′), 5.02 (br d, *J* = 3.2 Hz, 1H, H-1″), 4.01 (m, 1H, H-5″), 3.70–3.30 (m, 17H, H-1, H-3, H-4, H-5, H-6, H-2′, H-4′, H-5′, H-6′ (2H), H-2″, H-3″, H-4″, H-6″ (2H), OCH_2_CH_2_(CH_2_)_9_CH_3_), 2.09 (m, 1H, H-2eq), 1.99 (m, 1H, H-3′eq), 1.62 (app. q, *J*_1_ = *J*_2_ = *J*_3_ = 12.0 Hz, 1H, H-3′ax), 1.55 (p, *J* = 6.8 Hz, 2H, OCH_2_CH_2_(CH_2_)_9_CH_3_), 1.60–1.22 (m, 64H, H-2ax, 5× CO_2_(CH_3_)_3,_ OCH_2_CH_2_(CH_2_)_9_CH_3_), 0.88 (t, *J* = 7.2 Hz, 3H, OCH_2_CH_2_(CH_2_)_9_CH_3_); ^13^C-NMR (100 MHz, CD_3_OD, Figure S32) δ 158.1, 157.9, 156.5, 156.3 (2 carbons), 98.2, 97.8, 82.6, 80.9, 79.3, 78.9 (2 carbons), 78.7, 75.5, 72.3, 72.1, 71.6, 70.6, 69.6, 68.6, 65.1, 55.9, 50.2, 49.7, 49.6, 40.6, 34.2, 32.9, 31.7, 29.4 (2 carbons), 29.35, 29.25 (2 carbons), 29.23, 29.1 (2 carbons), 27.4 (15 carbons), 25.8, 22.3, 13.0; HR-MS (Figure S33) calcd for C_55_H_101_N_5_O_19_Na [M + Na]^+^ 1158.6988; found 1158.7010.

*O*-3-Amino-3-deoxy-6-*O*-dodecyl-α-d-glucopyranosyl-(1→6)-*O*-[2,6-diamino-2,3,6-trideoxy-α-d-*ribo*-hexopyranosyl-(1→4)]-2-deoxy-d-streptamine (**16**). Compound **15** (22 mg, 0.019 mmol) was treated at room temperature with neat TFA (1 mL) for 3 min. The TFA was removed under reduced pressure, the residue was dissolved in a minimal volume of H_2_O, extracted with CH_2_Cl_2_ (3×), and freeze-dried to afford compound **16** (22 mg, 96%) as a white foam: ^1^H-NMR (400 MHz, D_2_O, Figure S34) δ 5.61 (d, *J* = 3.2 Hz, 1H, H-1′), 4.90 (d, *J* = 4.0 Hz, 1H, H-1″), 3.85–3.70 (m, 4H, H-4, H-5′, H-2″, H-5″), 3.67 (app. t, *J*_1_ = *J*_2_ = 9.2 Hz, 1H, H-5), 3.62–3.28 (m, 11H, H-1, H-3, H-6, H-2′, H-4′, H-6′ (2H), H-3″, H-4″, OCH_2_(CH_2_)_10_CH_3_), 3.25 (dd, *J*_1_ = 14.0 Hz, *J*_2_ = 2.8 Hz, 1H, H-6″), 3.08 (dd, *J*_1_ = 13.2 Hz, *J*_2_ = 6.8 Hz, 1H, H-6″), 2.37 (app. dt, *J*_1_ = 12.4 Hz, *J*_2_ = *J*_3_ = 3.6 Hz, 1H, H-2eq), 2.12 (app. dt, *J*_1_ = 12.4 Hz, *J*_2_ = *J*_3_ = 4.0 Hz, 1H, H-3′eq), 1.85 (app. q, *J*_1_ = *J*_2_ = *J*_3_ = 12.0 Hz, 1H, H-3′ax), 1.76 (app. q, *J*_1_ = *J*_2_ = *J*_3_ = 12.8 Hz, 1H, H-2ax), 1.39 (p, *J* = 6.8 Hz, 2H, OCH_2_CH_2_(CH_2_)_9_CH_3_), 1.20–1.00 (m, 18H, OCH_2_CH_2_(CH_2_)_9_CH_3_), 0.67 (t, *J* = 7.2 Hz, 3H, OCH_2_(CH_2_)_10_CH_3_); ^13^C-NMR (100 MHz, D_2_O, Figure S35) δ 100.8, 93.9, 83.7, 77.0, 74.0, 71.89, 71.86, 70.3, 67.92, 67.86, 65.2, 64.3, 54.7, 49.5, 48.2, 47.6, 39.7, 31.1, 29.2, 28.7 (2 carbons), 28.62, 28.57, 28.5, 28.4, 28.3, 27.7, 25.0, 21.9, 13.3; HR-MS (Figure S36) calcd for C_30_H_62_N_5_O_9_ [M + H]^+^ 636.4542; found 636.4546.

### 4.3. Antifungal Susceptibility Testing

The MIC values of compounds **3**, **4**, **7a**, **7b**, **9**, and **16**, as well as the parent compound TOB and the antifungal CAS against yeasts (strains **A**–**G** (Table 1), and strains **H**–**J**, **CG1**–**3**, **CP1**–**3**, and **CN1**–**3** (Table 2)) were evaluated in 96-well microtiter plates as described in the CLSI document M27-A3 [52] with minor modifications. The final concentrations of compounds **3**, **4**, **7a**, **7b**, **9**, and **16**, as well as that of TOB tested in this study ranged from 0.24–62.5 μg/mL. CAS was used as a positive control and the final concentrations tested for CAS ranged from 0.03–31.3 μg/mL. Briefly, overnight yeast cultures were grown in yeast peptone dextrose (YPD) broth and the cell density was adjusted to an OD_600_ of 0.12 (~1 × 10^6^ CFU/mL) by using a spectrophotometer. Yeast cell suspensions were further diluted to achieve 1–5 × 10^3^ CFU/mL in RPMI 1640 medium, and 100 μL of these yeast cells was added to 96-well microtiter plates containing RPMI 1640 medium and titrated compounds. Each test was performed in triplicate. The plates were incubated at 35 °C for 48 h. The MIC values for compounds **3**, **4**, **7a**, **7b**, **9**, and **16**, CAS, and TOB were defined as the lowest drug concentration that prevented visible growth (also known as MIC-0) when compared to the growth control. These data are presented in Table 1 (for strains **A**–**G**) and Table 2 (for strains **H**–**J**, **CG1**–**3**, **CP1**–**3**, and **CN1**–**3**).

Similarly, the MIC values of compounds **3**, **4**, **7a**, **7b**, **9**, and **16**, as well as that of all control drugs against filamentous fungi (strains **K**–**M** (Table 3)) were determined as previously described in CLSI document M38-A2 [53]. The spores were harvested from sporulating cultures growing on potato dextrose agar (PDA) by filtration through sterile glass wool and enumerated by using a hemocytometer (Hausser Scientific, Horsham, PA, USA) to obtain the desired inoculum size. Two-fold serial dilutions of compounds **3**, **4**, **7a**, **7b**, **9**, and **16**, as well as CAS and TOB were made in sterile 96-well microtiter plates to obtain the final concentration range of 0.24–62.5 μg/mL for TOB and TOB derivatives as well as 0.03–31.3 μg/mL for CAS in the RPMI 1640 medium. The spore suspensions were added to the wells to afford a final concentration of 5 × 10^5^ spores/mL. The plates were incubated at 35 °C for 72 h. The MIC values of all compounds, including compounds **3**, **4**, **7a**, **7b**, **9**, and **16** as well as CAS and TOB against filamentous fungi were based on the complete inhibition of growth (optically clear well) when compared to the growth control (MIC-0). Each test was performed in triplicate. These data are also presented in Table 3 (strains **K**–**M**).

### 4.4. Hemolytic Activity Assays

The hemolytic activity of compounds **3**, **4**, **7a**, **7b**, and **9** as well as the parent compound TOB was determined by using previously described methods with minor modifications [23]. Murine whole blood (1 mL) was suspended in 4 mL of PBS and centrifuged at 1000 rpm for 10 min at room temperature to obtain murine red blood cells (mRBCs). The mRBCs were washed four times in PBS and resuspended in the same buffer to a final concentration of 10^7^ erythrocytes/mL. Two-fold serial dilutions of compounds **3**, **4**, **7a**, **7b**, and **9** were prepared using 100 μL of PBS buffer in Eppendorf tubes followed by the addition of 100 μL of mRBC suspension that made the final concentration of compounds and mRBCs to be 0.48–62.5 μg/mL and 5 × 10^6^ erythrocytes/mL, respectively. The tubes were incubated at 37 °C for 1 h. The tubes with PBS buffer (200 μL) and Triton X-100^®^ (1% *v*/*v*, 2 μL) served as the negative (blank) and positive controls, respectively. The percentage of hemolysis was calculated using the following equation: % hemolysis = [(absorbance of sample) − (absorbance of blank)] × 100/(absorbance of positive control). These data are presented in Figure 1 and Table S1.

### 4.5. In Vitro Cytotoxicity Assays

Mammalian cytotoxicity assays were performed as previously described with minor modifications [29]. The normal human bronchial epithelial cells BEAS-2B and the human lung carcinoma epithelial cells A549 were grown in DMEM containing 10% fetal bovine serum (FBS) and 1% antibiotics. The confluent cells were then trypsinized with 0.05% trypsin and 0.53 mM EDTA, and resuspended in fresh DMEM medium. The cells were transferred into 96-well microtiter plates at a density of 3000 cells/well and were grown for 24 h. The following day, the medium was replaced by a fresh culture medium containing serially diluted compounds **3**, **4**, **7a**, **7b**, and **9**, as well as the parent drug TOB at final concentrations of 0.48–62.5 μg/mL or sterile ddH_2_O (negative control). The cells were incubated for an additional 24 h at 37 °C with 5% CO_2_ in a humidified incubator. To evaluate cell survival, each well was treated with 10 μL (25 μg/mL) of resazurin sodium salt (Sigma-Aldrich) for 4–6 h. Metabolically active cells can convert resazurin (blue) to the highly fluorescent dye resorufin (pink), which was detected at λ_560_ excitation and λ_590_ emission wavelengths by using a SpectraMax M5 plate reader. Triton X-100^®^ (1% *v*/*v*) gave the complete loss of cell viability and was used as the positive control. The percentage survival rates were calculated by using the following formula: % cell survival = [(fluorescence of sample) − (fluorescence of blank)] × 100/[(fluorescence of negative control) − (fluorescence of blank)]. These data are presented in Figure 2.

### 4.6. Time-Kill Assays

Time-kill assays were used to assess the inhibitory efficiency of **7b** against three yeast strains, *C. albicans* ATCC 10231 (strain **A**), *C. parapsilosis* ATCC 22019 (strain **J**), and *C. neoformans* clinical isolate **CN1**. The protocol for time-kill assays followed methods previously described [29,54] with minor modifications. Yeast cultures were grown overnight in YPD broth at 35 °C with shaking (200 rpm). A working stock of fungal cells was made by diluting cultures in RPMI 1640 medium to an OD_600_ of 0.125, approximately 1 × 10^6^ CFU/mL. From the working stock, 100 μL of cells was added to 4.9 mL of RPMI 1640 medium in sterile culture tubes, making the starting fungal cell concentration approximately 10^5^ CFU/mL. Compounds were then added to the fungal cells. The treatment conditions included sterile control, growth control, TOB (parent; negative control), voriconazole (VOR) at 1× MIC (positive control), **7b** at 1× and 4× MIC. The treated fungal cultures were incubated at 35 °C with shaking (200 rpm) for 24 h. The samples were taken from the different treatments at regular time points (0, 3, 6, 9, 12, and 24 h.) and plated in duplicates. For each time point, cultures were vortexed, 100 μL of culture was aspirated, and 10-fold serial dilutions were made in sterile ddH_2_O. From the appropriate dilutions, 100 μL of fungal suspension was spread on PDA plates and incubated at 35 °C for 48 h before the colony counts were determined. At 24 h, 50 μL of 1 mM resazurin was added to the treatments and incubated at 35 °C with rotation for 2 h in the dark for visual inspection. Experiments were performed in duplicate. These data are presented in Figure 3.

### 4.7. Membrane Permeabilization Assay Using Propidium Iodide Staining

A fresh culture of *C. albicans* ATCC 10231 (strain **A**) was prepared in 5 mL of YPD broth in a Falcon tube and was grown overnight at 35 °C at 200 rpm. An overnight culture (40 μL) was transferred to RPMI 1640 medium (1 mL) containing no drug (negative control) or compound **7b** at concentrations of 3.9 μg/mL (2× MIC) and 7.8 μg/mL (4× MIC). TOB (31.3 μg/mL) was used as a negative control. The cell suspensions were then treated for 2 h at 35 °C with continuous agitation (200 rpm). To prepare a positive control sample, *C. albicans* ATCC 10231 (strain **A**) cells were killed by heat shock at 95 °C for 5 min as described by Ocampo and Barrientos [55]. The cells were then centrifuged and resuspended in 500 μL of PBS buffer (pH 7.2 adjusted at room temperature). Subsequently, the cells were treated with propidium iodide (9 μM, final concentration) and incubated for 20 min at room temperature in the dark. Glass slides prepared with 10 μL of each mixture were observed in bright field and fluorescence modes (using Texas red filter set with λ_535_ excitation and λ_617_ emission wavelengths) using a Zeiss Axiovert 200 M fluorescence microscope. The data were obtained from at least two independent experiments. The images were also post-processed, utilizing automatic contrast and brightness setting in Microsoft PowerPoint 2013 to eliminate background noise. These images are presented in Figure 4.

## Figures and Tables

**Table 1:** The minimum inhibitory concentration (MIC) values ^a^ (in μg/mL) determined for tobramycin (TOB); its derivatives **3**, **4**, **7a**, **7b**, **9**, and **16**; as well as CAS, against a panel of seven *C*. *albicans* strains.

	Yeast Strains						
Cpd #	A	B	C	D	E	F	G
**TOB**	>62.5	>62.5	>62.5	>62.5	>62.5	>62.5	>62.5
**3**	15.6	15.6	15.6	15.6	15.6	7.8	15.6
**4**	15.6	31.3	15.6	15.6	15.6	15.6	15.6
**7a**	31.3	31.3	62.5	62.6	31.3	62.5	62.5
**7b**	3.9	3.9	15.6	15.6	7.8	7.8	7.8
**9**	31.3	15.6	31.3	62.5	31.3	31.3	31.3
**16**	62.5	62.5	>62.5	62.5	62.5	62.5	62.5
**CAS**	0.975	0.24	0.06	0.12	0.12	0.24	0.48

^a^ MIC-0 values are reported for all compounds tested. **Yeast strains**: **A** = *Candida albicans* ATCC 10231, **B** = *C. albicans* ATCC 64124, **C** = *C. albicans* ATCC MYA-2876(S), **D** = *C. albicans* ATCC 90819(R), **E** = *C. albicans* ATCC MYA-2310(S), **F** = *C. albicans* ATCC MYA-1237(R), **G** = *C. albicans* ATCC MYA-1003(R). NOTE: Here, the (S) and (R) indicate that ATCC reports these strains to be susceptible (S) or resistant (R) to itraconazole and fluconazole.

**Table 2:** The MIC values ^a^ (in μg/mL) determined for TOB; its derivatives **3**, **4**, **7a**, **7b**, **9**, and **16**; as well as CAS, against a panel of nine non-*albicans Candida* and three strains of *Cryptococcus neoformans*.

	Yeast Strains											
	Non-*albicans Candida*									*Cryptococcus neoformans*		
Cpd #	CG1	CG2	CG3	H	I	J	CP1	CP2	CP3	CN1	CN2	CN3
**TOB**	>62.5	>62.5	>62.5	>62.5	>62.5	>62.5	>62.5	>62.5	>62.5	>62.5	>62.5	>62.5
**3**	15.6	7.8	15.6	7.8	3.9	7.8	15.6	7.8	15.6	0.975	0.48	0.48
**4**	7.8	3.9	3.9	15.6	7.8	7.8	3.9	3.9	7.8	1.95	0.48	0.975
**7a**	31.3	15.6	15.6	62.5	15.6	15.6	15.6	15.6	31.3	0.975	0.975	0.48
**7b**	3.9	3.9	3.9	7.8	3.9	1.95	3.9	1.95	7.8	0.48	0.48	0.975
**9**	15.6	15.6	15.6	31.3	15.6	7.8	31.3	15.6	31.3	0.975	0.975	0.975
**16**	>62.5	62.5	62.5	62.5	31.3	31.3	62.5	62.5	62.5	1.95	0.975	3.9
**CAS**	1.95	0.24	0.975	0.06	0.48	1.95	0.48	0.48	0.48	15.6	31.3	15.6

^a^ MIC-0 values are reported for all compounds tested. **Yeast strains**: **CG1**–**3** = *Candida glabrata* clinical isolates, **CP1**–**3** = *Candida parapsilosis* clinical isolates, **CN1**–**3** = *Cryptococcus neoformans* clinical isolates, **H** = *Candida glabrata* ATCC 2001, **I** = *Candida krusei* ATCC 6258, **J** = *Candida parapsilosis* ATCC 22019.

**Table 3:** The MIC values ^a^ (in μg/mL) determined for TOB; its derivatives **3**, **4**, **7a**, **7b**, **9**, and **16**; as well as CAS, against a panel of three *Aspergillus* strains.

	Filamentous Strains		
Cpd #	K	L	M
**TOB**	>62.5	>62.5	>62.5
**3**	15.6	3.9	>62.5
**4**	31.3	3.9	62.5
**7a**	31.3	3.9	>62.5
**7b**	7.8	1.95	62.5
**9**	31.3	3.9	>62.5
**16**	>62.5	3.9	>62.5
**CAS**	>31.3	>31.3	>31.3

^a^ MIC-0 values are reported for all compounds tested. **Filamentous strains**: **K** = *Aspergillus flavus* ATCC MYA-3631, **L** = *Aspergillus nidulans* ATCC 38163, **M** = *Aspergillus terreus* ATCC MYA-3633.

## Supplementary Materials

The supplementary materials include ^1^H and ^13^C-NMR as well as mass spectra (Figures S1–S36) for the molecules synthesized. All compounds tested for activity are ≥95% pure according to NMR spectra. Table S1 with the % hemolysis ± SDEV displayed in Figure 1 is also provided. These materials are available free of charge via the Internet.

## Abbreviations

AcOH	acetic acid
AG	aminoglycoside
AmB	amphotericin B
Boc_2_O	di-*tert*-butyl dicarbonate
CAS	caspofungin
CFU	colony forming unit
DIPEA	*N*,*N*-diisopropylamine
DMF	*N*,*N*-dimethylformamide
EDC·HCl	*N*-(3-dimethylaminopropyl)-*N*′-ethylcarbodiimide hydrochloride
FDA	US food and drug administration
HOBt	hydroxybenzotriazole
KANA	kanamycin A
KANB	kanamycin B
*m*-CPBA	*meta*-chloroperbenzoic acid
mRBC	murine red blood cell
MIC	minimum inhibitory concentration
PivCl	pivaloyl chloride
TBAA	tetrabutylammonium azide
TFA	trifluoroacetic acid
Tf_2_O	trifluoromethane sulfonic anhydride
THF	tetrahydrofuran
TIPBSCl	2,4,6-triisopropylbenzenesulfonyl chloride
TOB	tobramycin
VOR	voriconazole
