# Supplementary material for: Differential Effects of Linkers on the Activity of Amphiphilic Tobramycin Antifungals
Source: Molecules. 2018 Apr 13;23(4):899. doi: 10.3390/molecules23040899 (PMC5971061; doi:10.3390/molecules23040899)
Supplement: Supplementary file 1 [file molecules-23-00899-s001.pdf]

Supplementary Materials

Differential effects of linkers on the activity of amphiphilic tobramycin antifungals

Marina Y. Fosso,[a] Sanjib K. Shrestha,[a] Nishad Thamban Chandrika,[a] Emily K. Dennis,[a] Keith D. Green,[a] Sylvie Garneau-Tsodikova[a,*]

[a] University of Kentucky, College of Pharmacy, Department of Pharmaceutical Sciences, Lexington, KY, USA, 40536-0596.

*sylviegtsodikova@uky.edu

Table of Content **Page #**

Table S1 S1

Figures S1-S36 S2-S26

Table S1: The % hemolysis caused by TOB derivatives **3**, **4**, **7a**, **7b**, and **9** as well as AmB against mRBCs with the error bars (± SDEV).[a]

	Compound #						
Concentration (μg/mL)	**3**	**4**	**7a**	**7b**	**9**	TOB	AmB
0.48	0	0	0	0	0	4.7 ±3.9	0
0.975	2.5 ± 2.3	0	0	0	0	9.3 ± 3.5	0
1.95	6.0 ± 3.5	6.6 ± 3.5	15.6 ± 9.3	14.8 ± 3.5	2.5 ± 1.5	15.9 ± 1.9	1.7 ± 0.1
3.9	12.0 ± 1.9	6.0 ± 1.9	0	13.4 ± 6.2	4.1 ± 1.5	15.9 ± 8.1	3.4 ± 0.6
7.8	18.6 ± 6.6	10.7 ± 3.1	7.4 ± 3.1	24.6 ± 8.1	10.1 ± 4.6	11.2 ± 4.6	3.7 ± 0.7
15.6	46.2 ± 2.3	15.3 ± 5.0	14.0 ± 5.4	40.8 ± 5.4	18.3 ± 5.4	15.3 ± 5.0	31.9 ± 5.7
31.3	55.5 ± 3.9	24.9 ± 2.3	14.8 ± 1.9	55.5 ± 5.4	26.0 ± 11.6	15.3 ± 2.7	80.2 ± 8.5
62.5	67.0 ± 4.6	31.2 ± 6.6	14.8 ± 0.4	74.1 ± 0.8	31.5 ± 1.5	21.1 ± 9.3	90.3 ± 5.7

[a] These are the values that were used to generate Fig. 1.

Figures S1-S36:

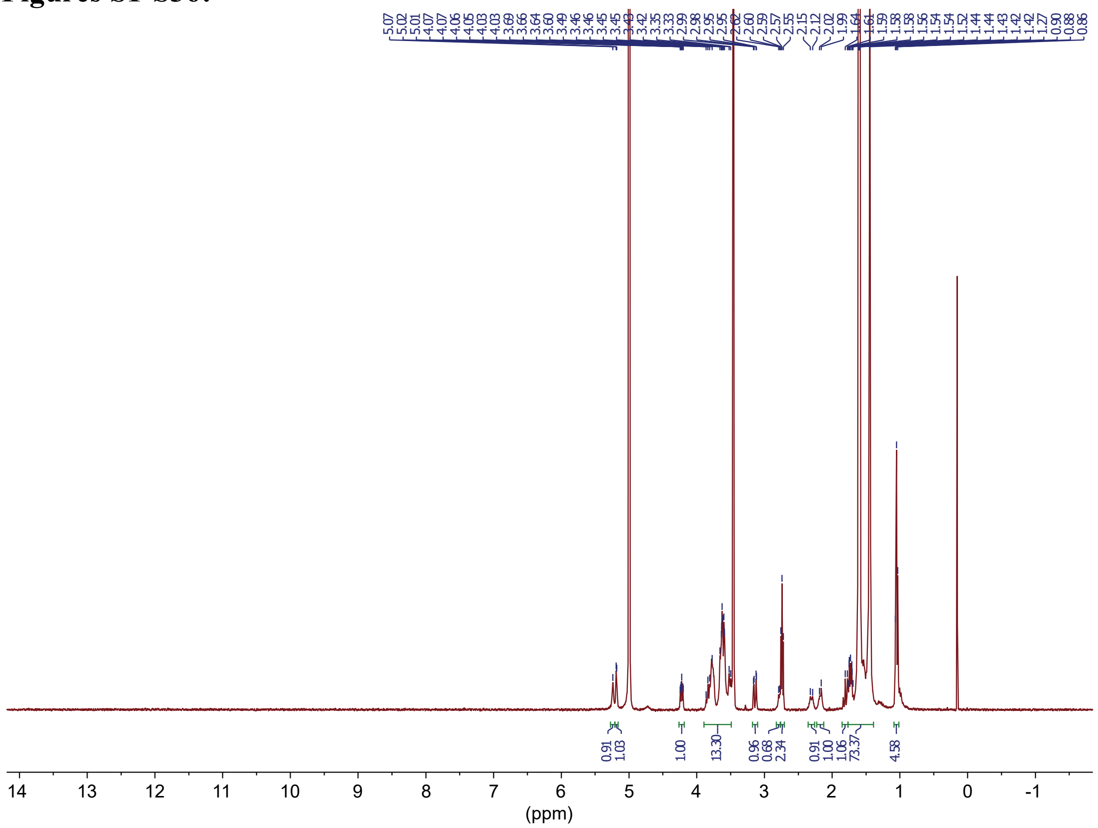

Fig. S1: ^{1}H NMR spectrum for compound **2** in CD_3OD (400 MHz).

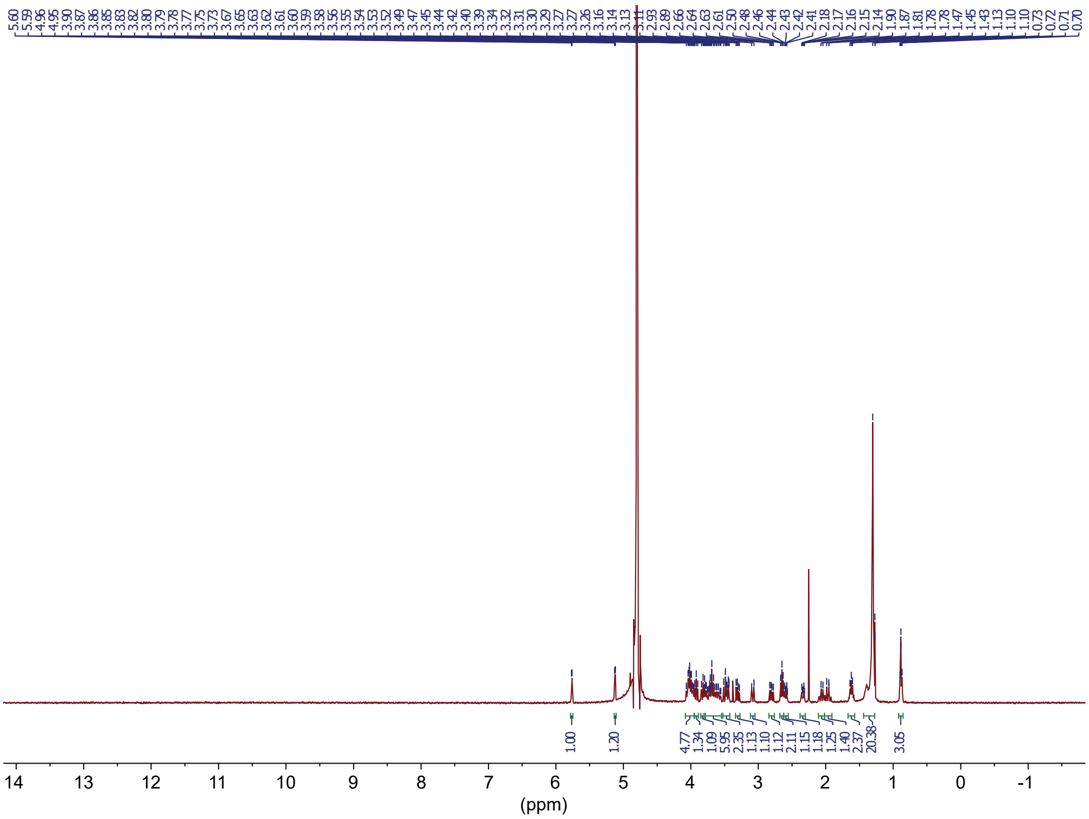

Fig. S2: ^{1}H NMR spectrum for compound **3** in D_2O (400 MHz).

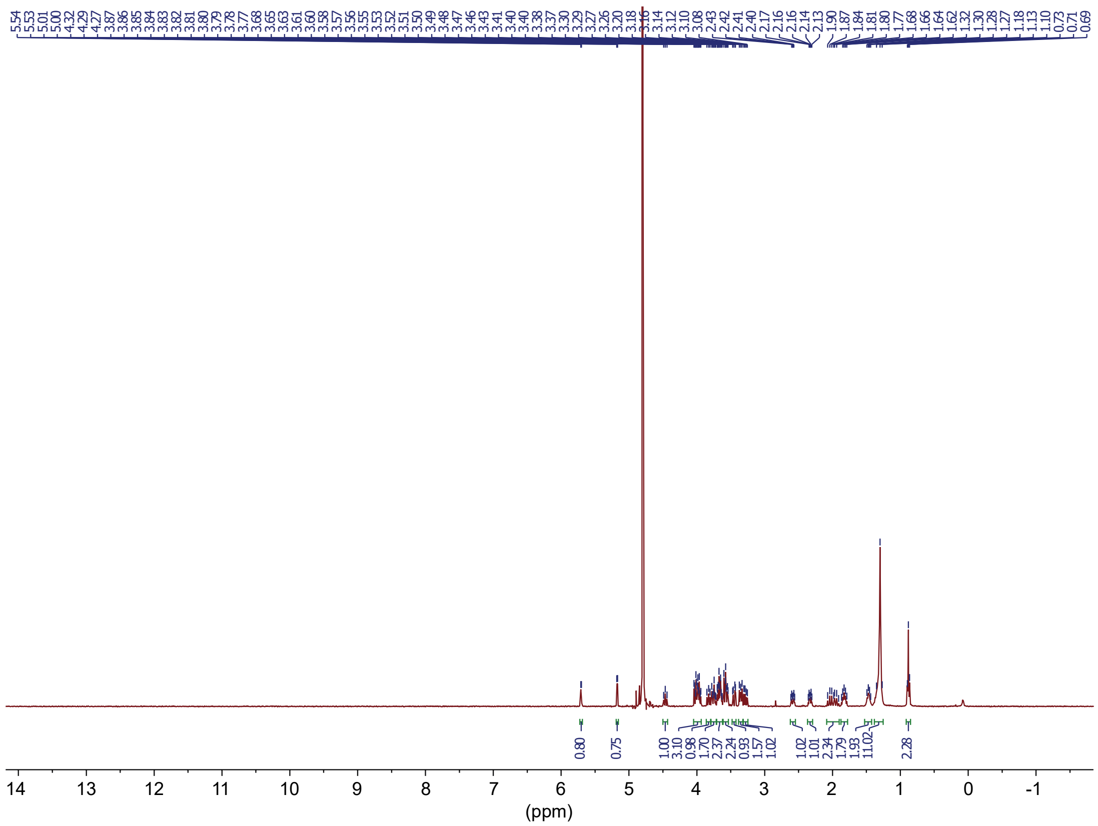

Fig. S3: ^{1}H NMR spectrum for compound **4** in D_2O (400 MHz).

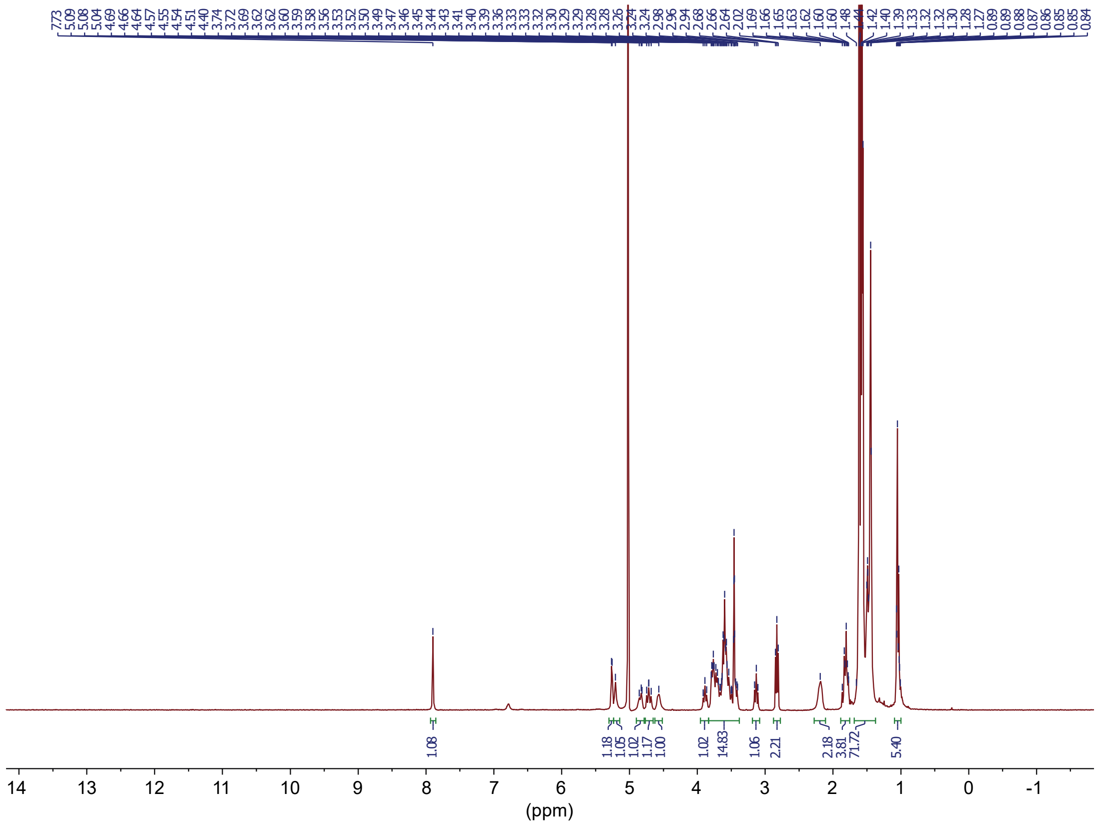

Fig. S4: ^{1}H NMR spectrum for compound **6a** in CD_3OD (400 MHz).

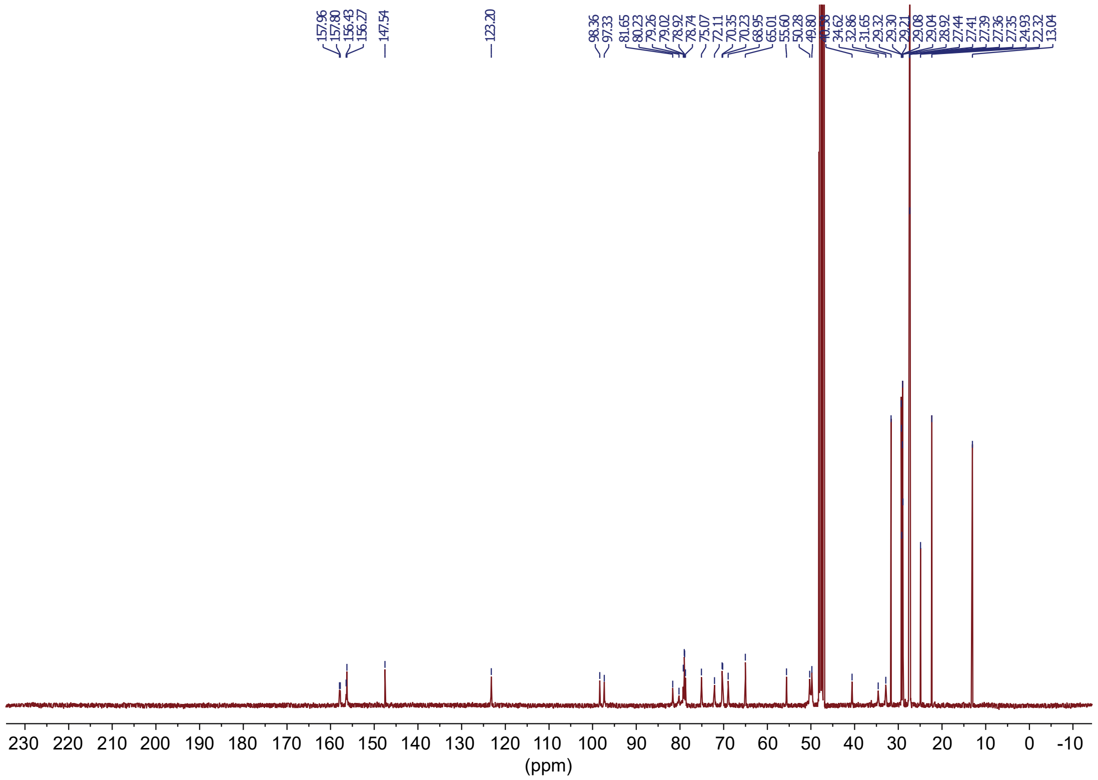

Fig. S5: ^{13}C NMR spectrum for compound **6a** in CD_3OD (100 MHz).

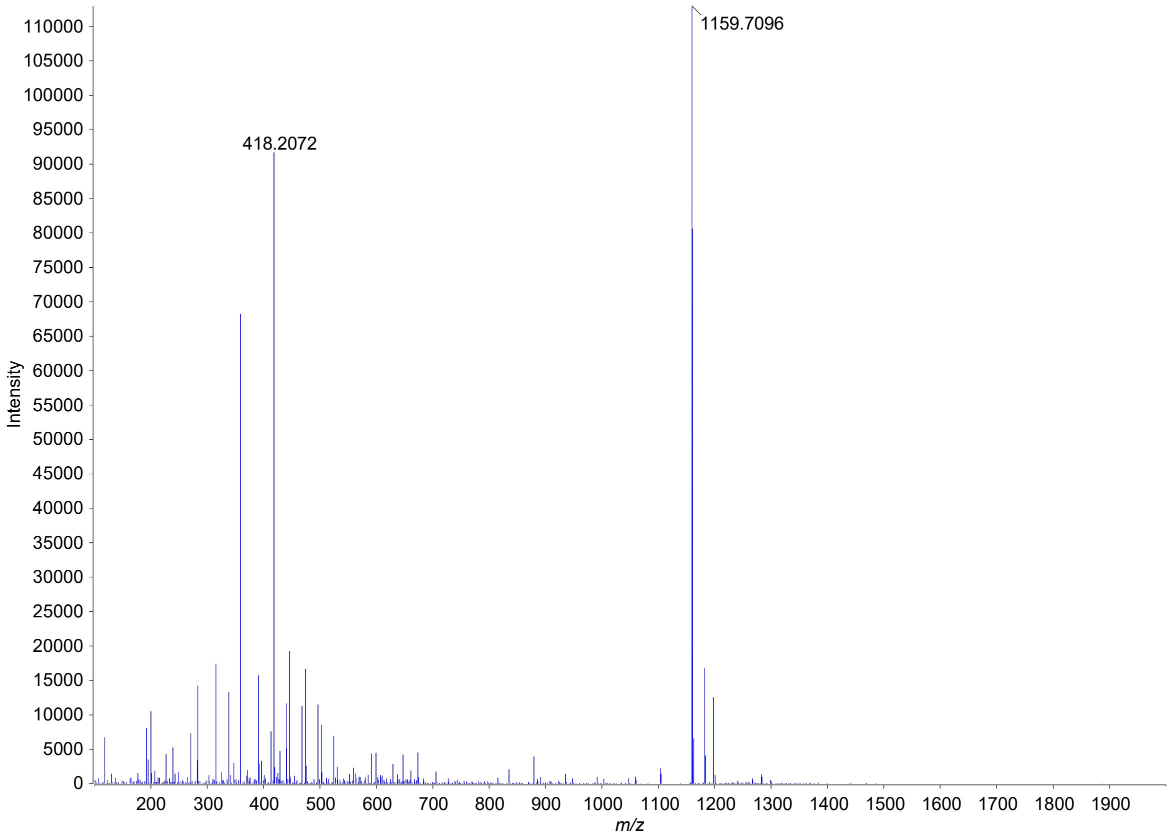

Fig. S6: Mass spectrum for compound **6a**.

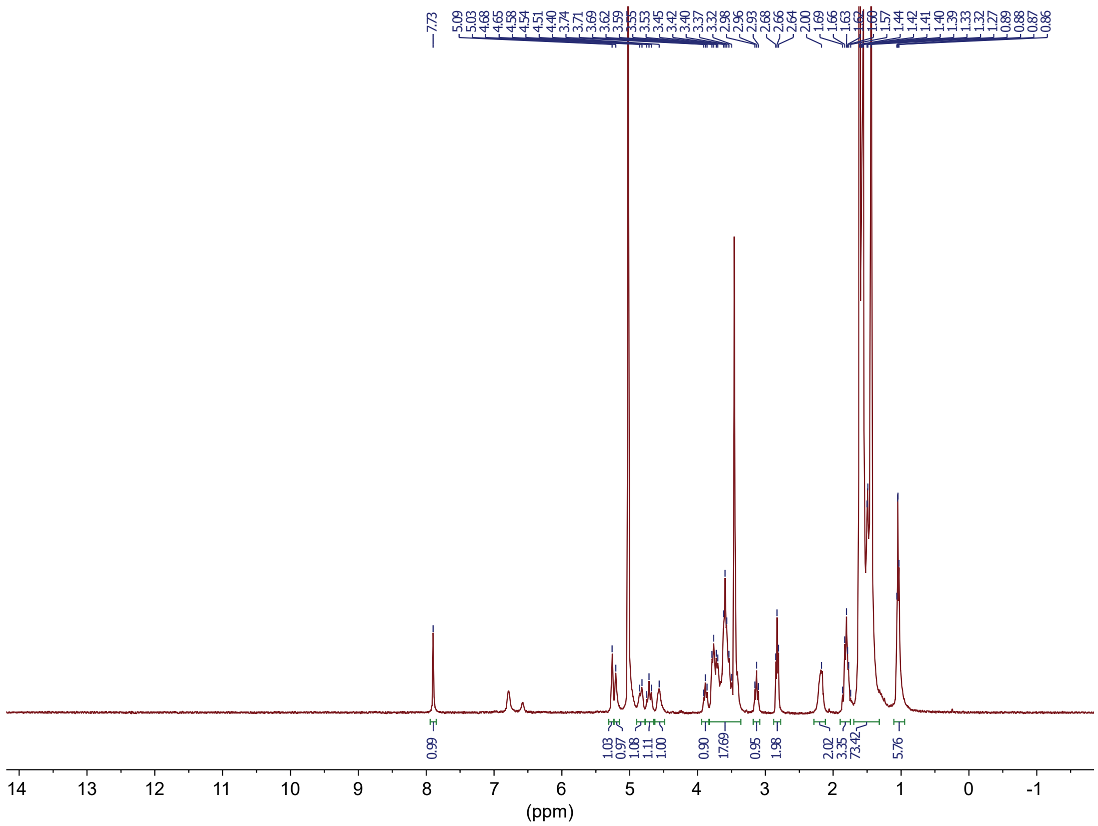

Fig. S7: ^{1}H NMR spectrum for compound **6b** in CD_3OD (400 MHz).

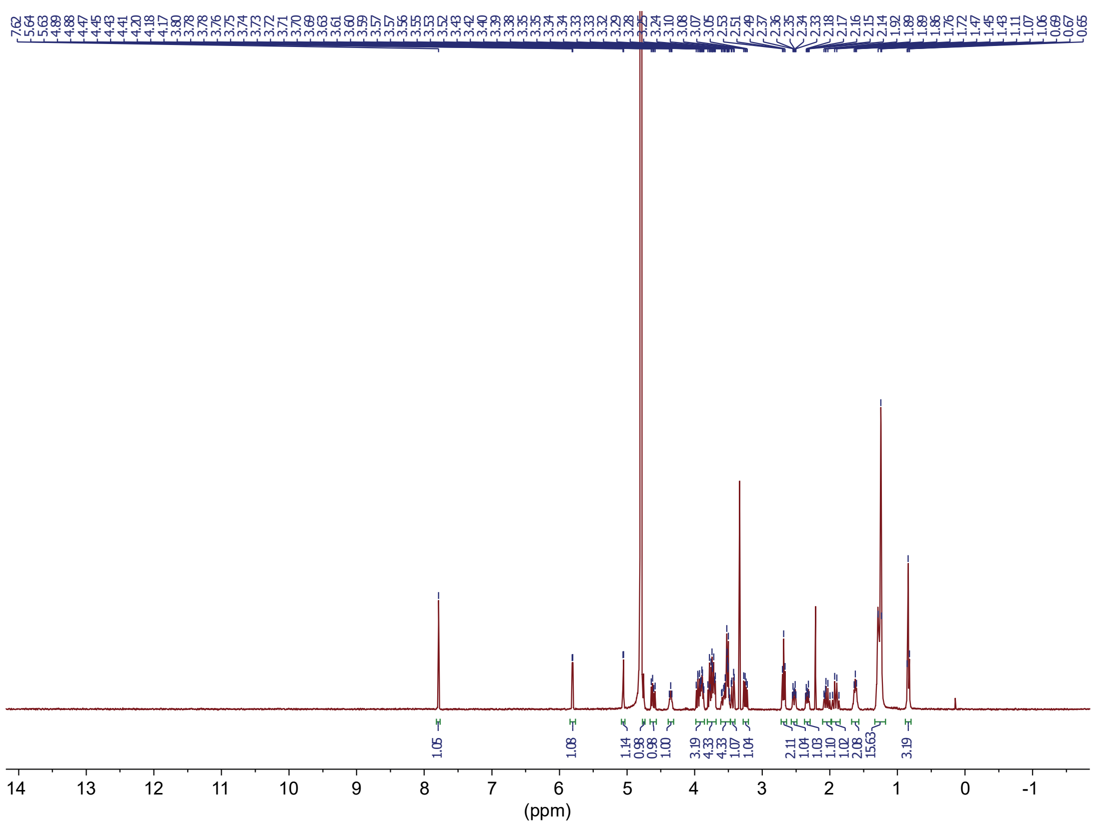

Fig. S8: ^{1}H NMR spectrum for compound **7a** in D_2O (400 MHz).

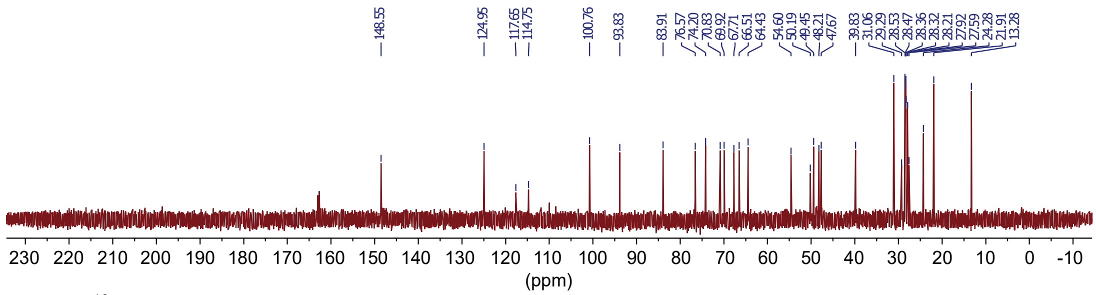

Fig. S9: ^{13}C NMR spectrum for compound **7a** in D_2O (100 MHz).

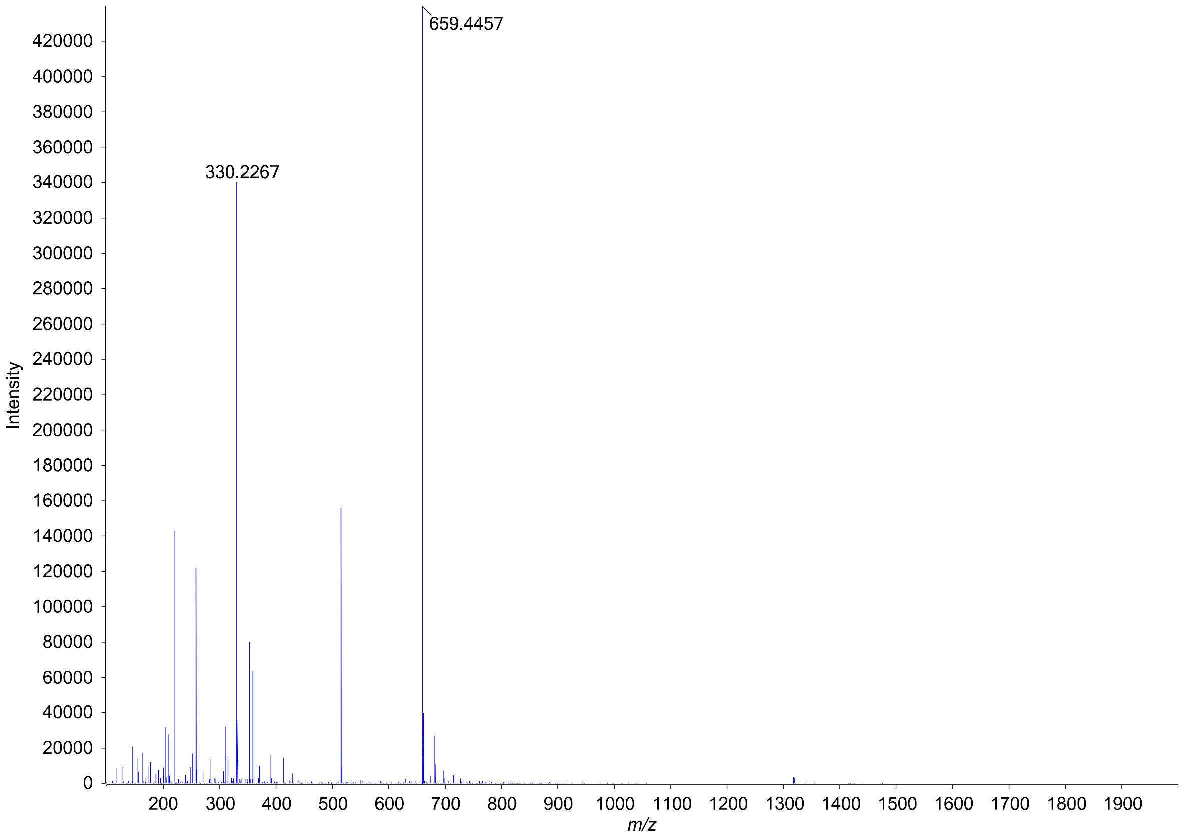

Fig. S10: Mass spectrum for compound **7a**.

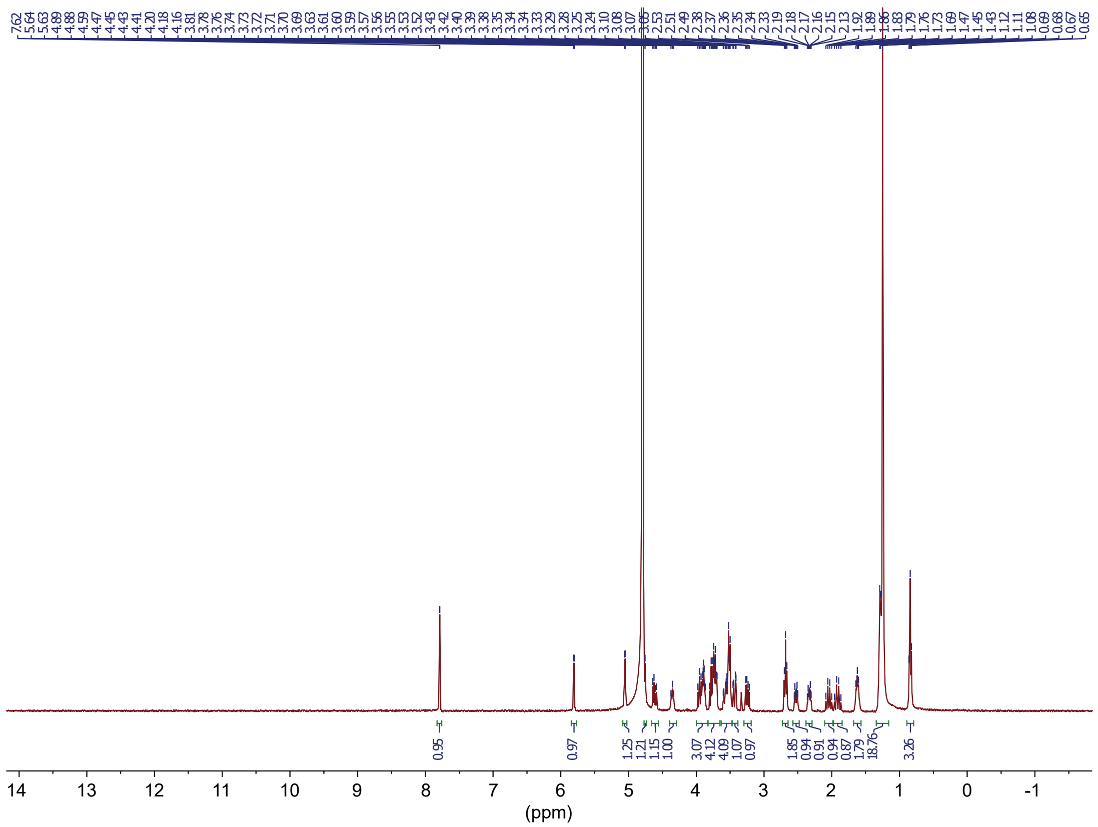

Fig. S11: ^{1}H NMR spectrum for compound **7b** in D_2O (400 MHz).

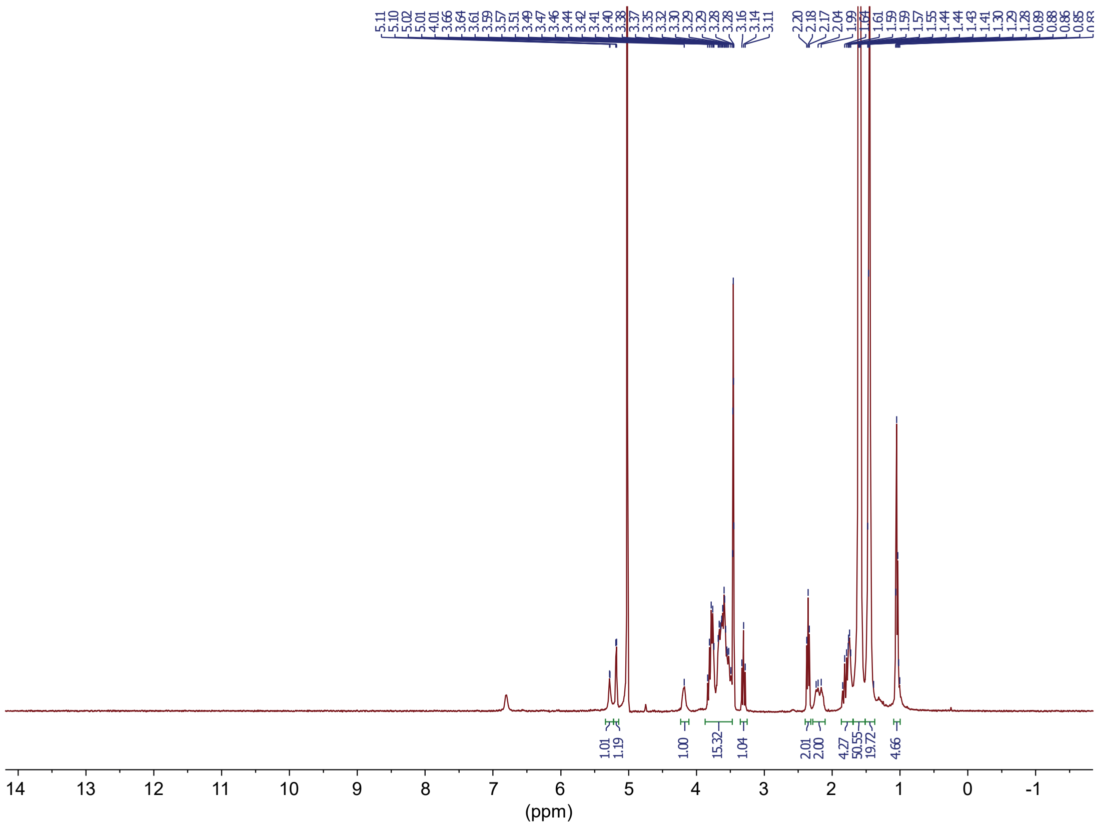

Fig. S12: ^{1}H NMR spectrum for compound **8** in CD_3OD (400 MHz).

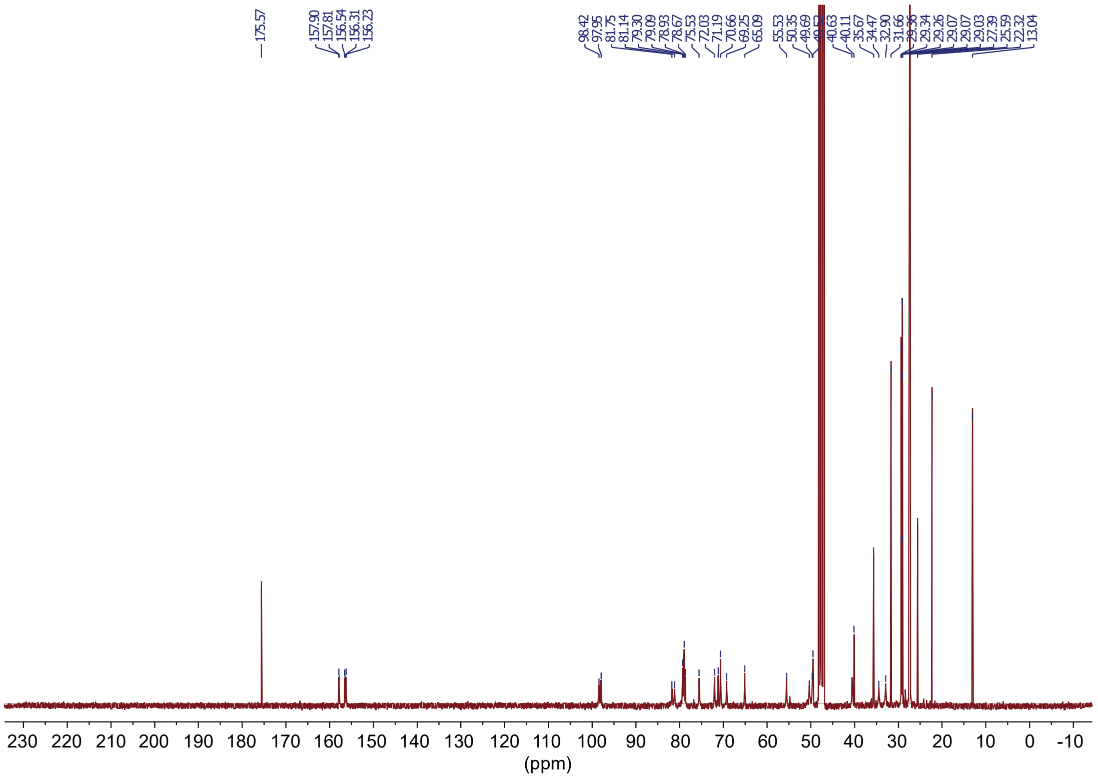

Fig. S13: ^{13}C NMR spectrum for compound **8** in CD_3OD (100 MHz).

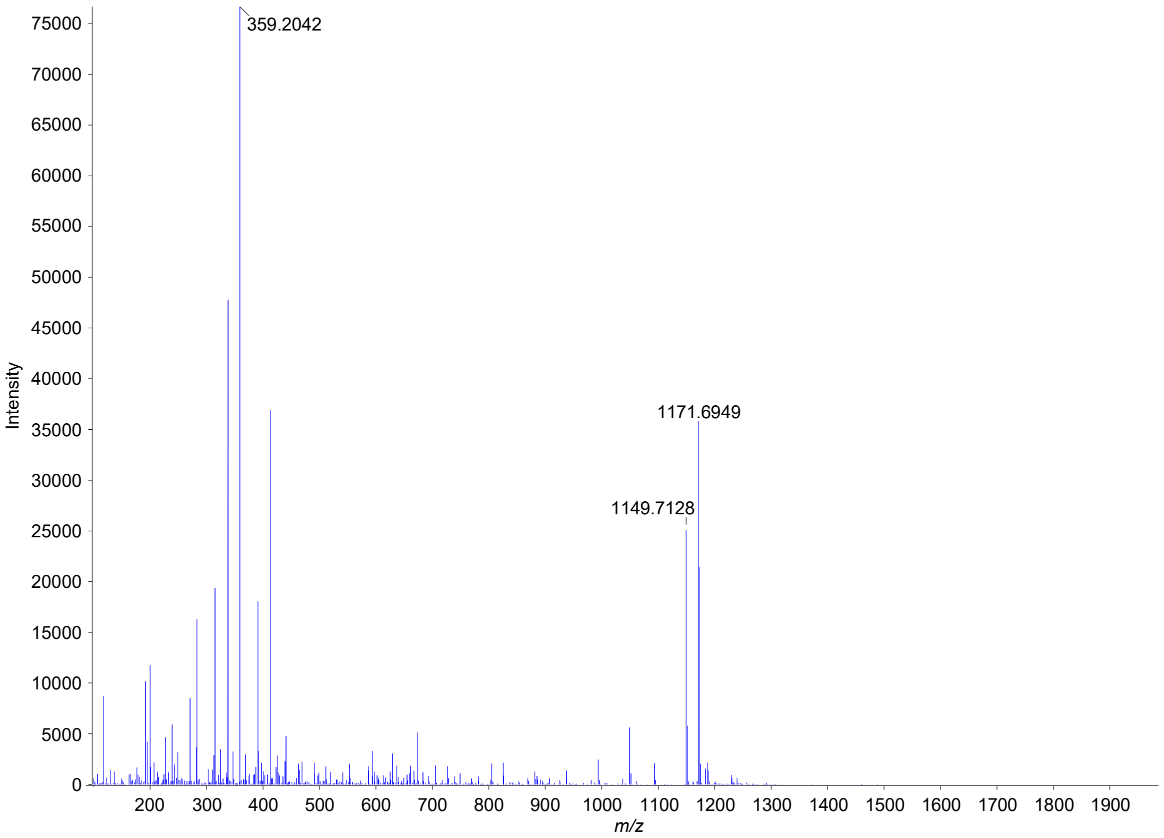

Fig. S14: Mass spectrum for compound **8**.

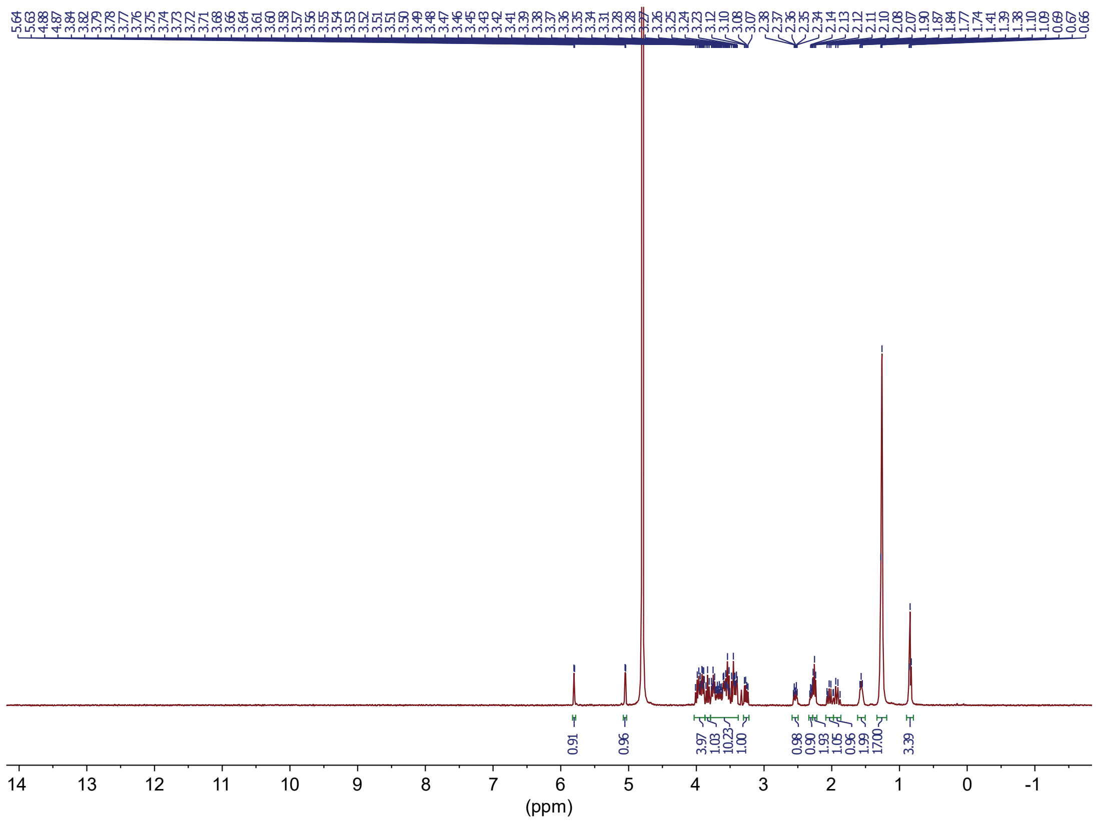

Fig. S15: ^{1}H NMR spectrum for compound **9** in D_2O (400 MHz).

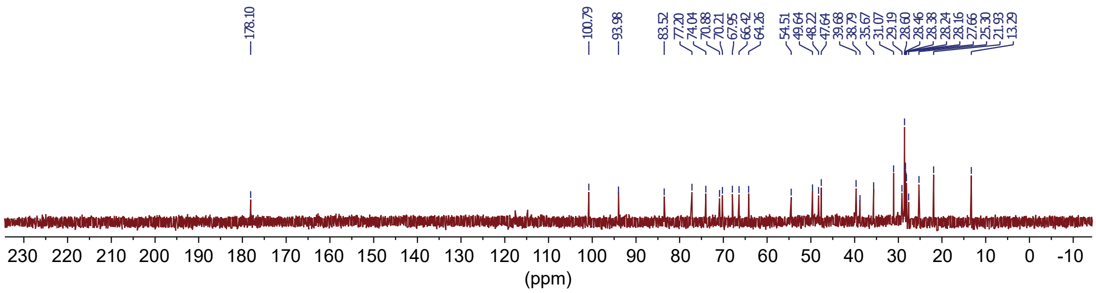

Fig. S16: ^{13}C NMR spectrum for compound **9** in D_2O (100 MHz).

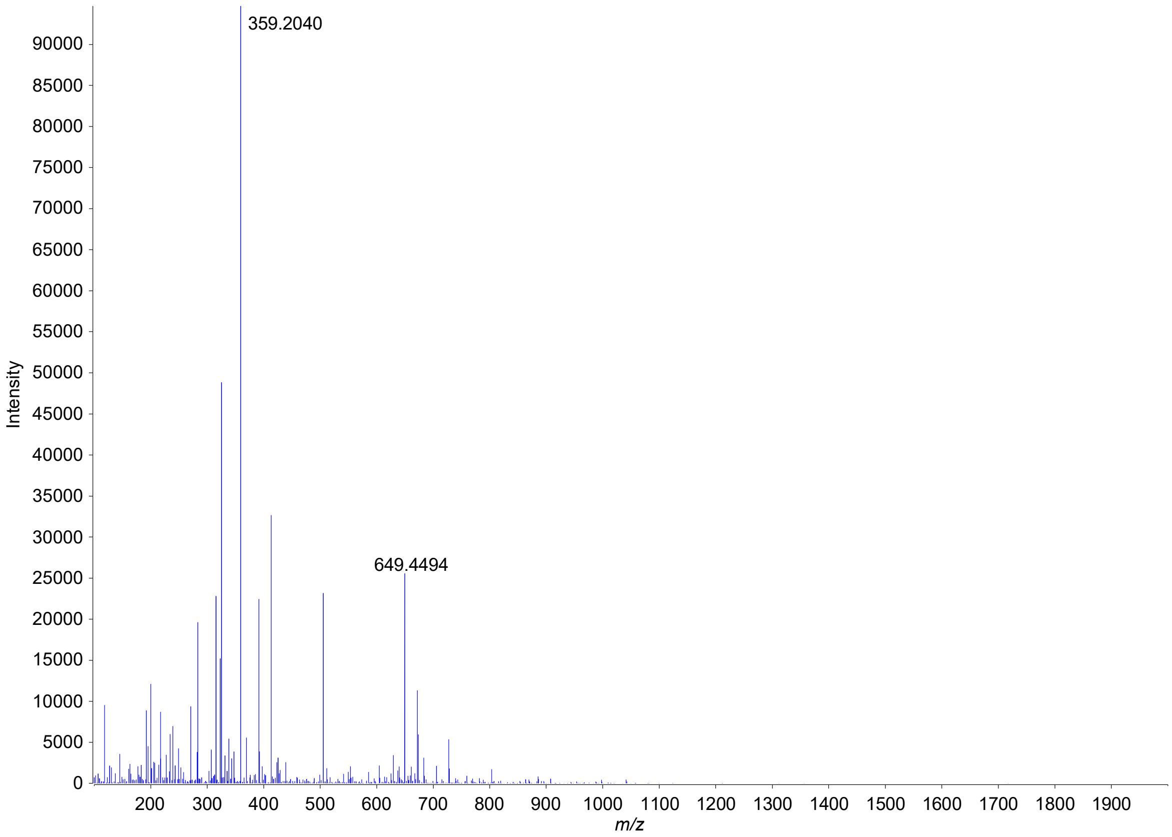

Fig. S17: Mass spectrum for compound **9**.

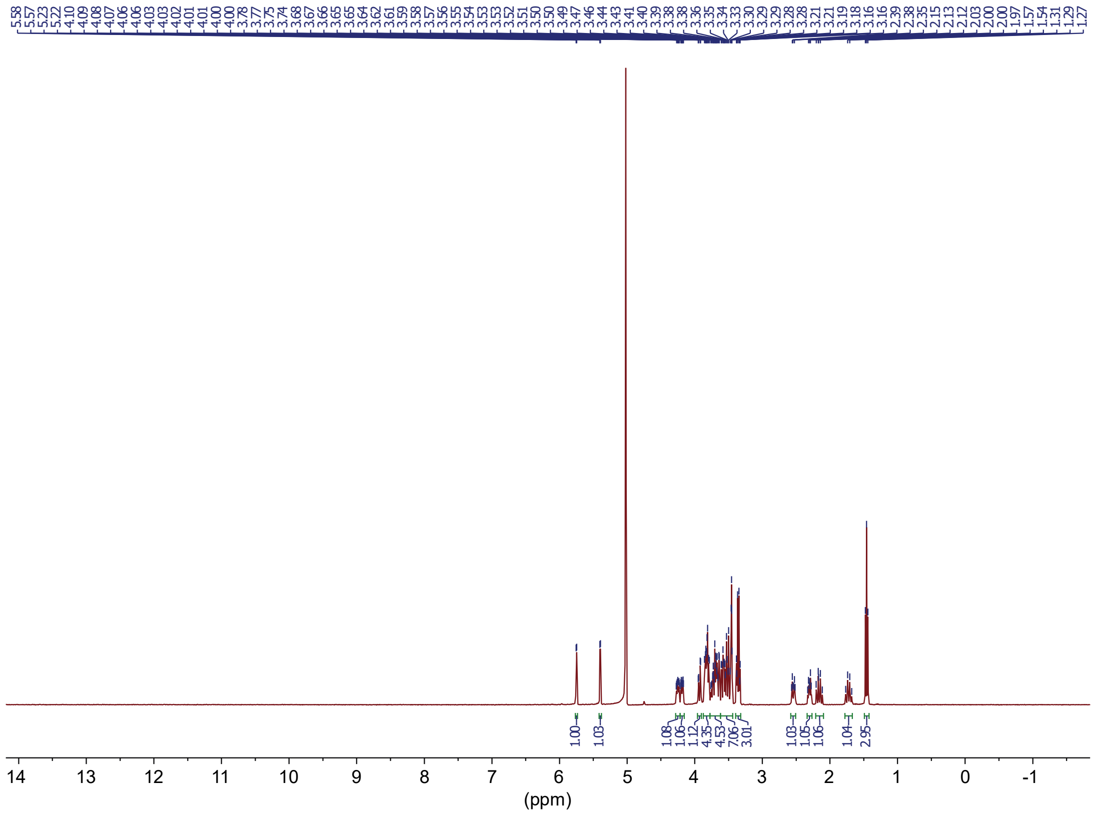

Fig. S18: ^{1}H NMR spectrum for compound **10** in CD_3OD (400 MHz).

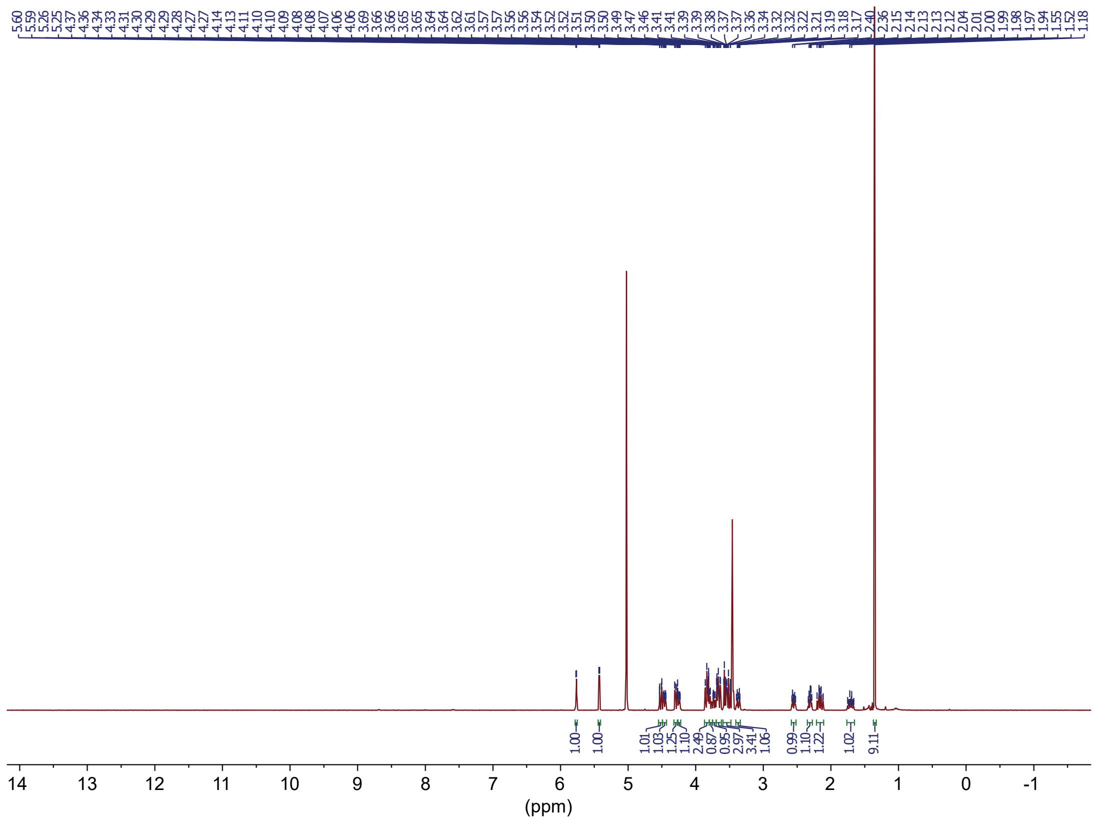

Fig. S19: ^{1}H NMR spectrum for compound **11** in CD_3OD (400 MHz).

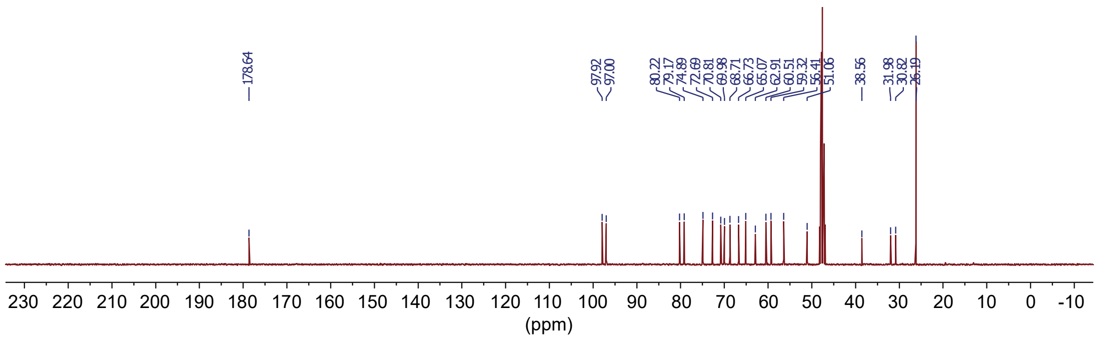

Fig. S20: ^{13}C NMR spectrum for compound **11** in CD_3OD (100 MHz).

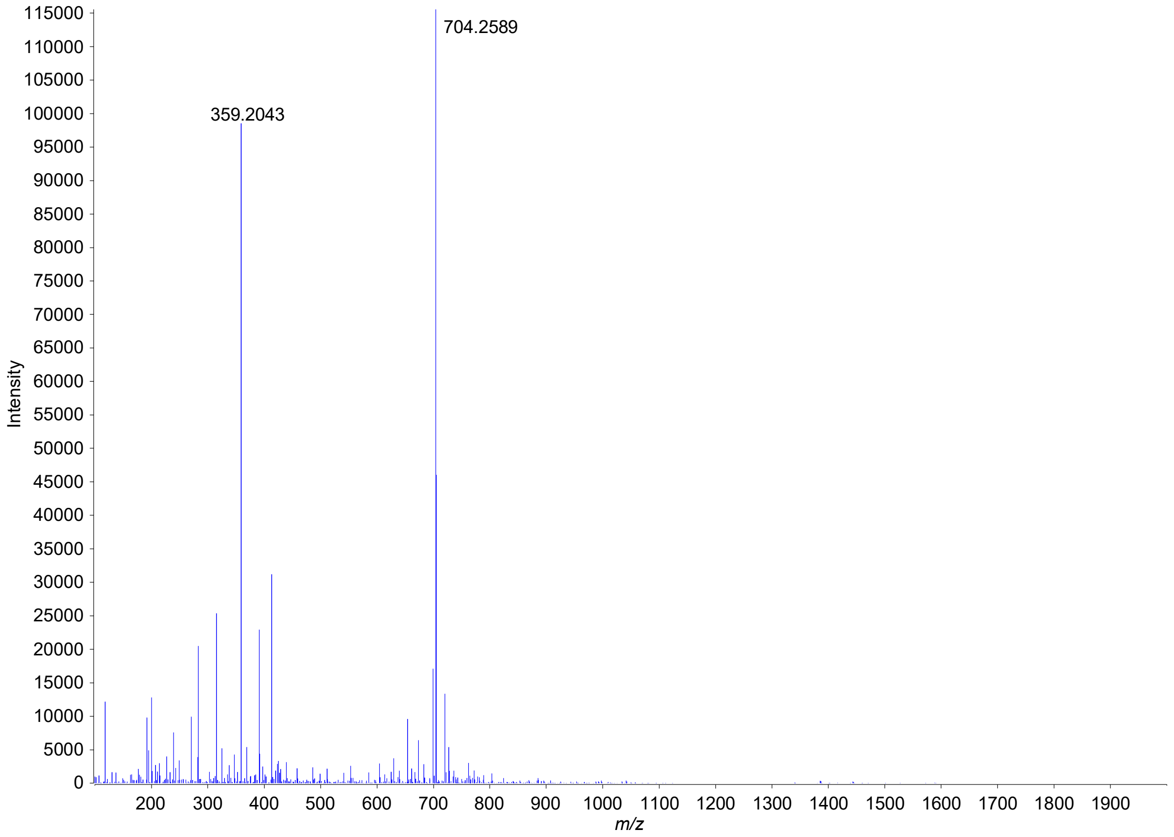

Fig. S21: Mass spectrum for compound **11**.

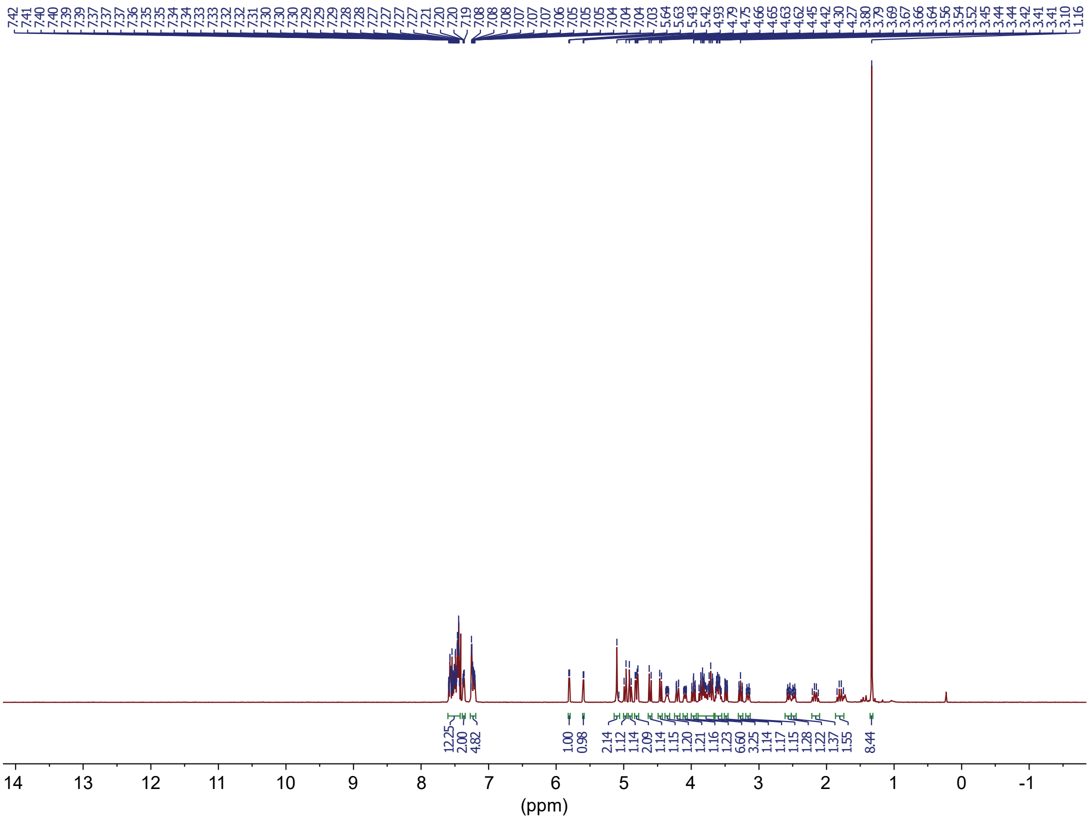

Fig. S22: ^{1}H NMR spectrum for compound **12** in $CDCl_3$ (400 MHz).

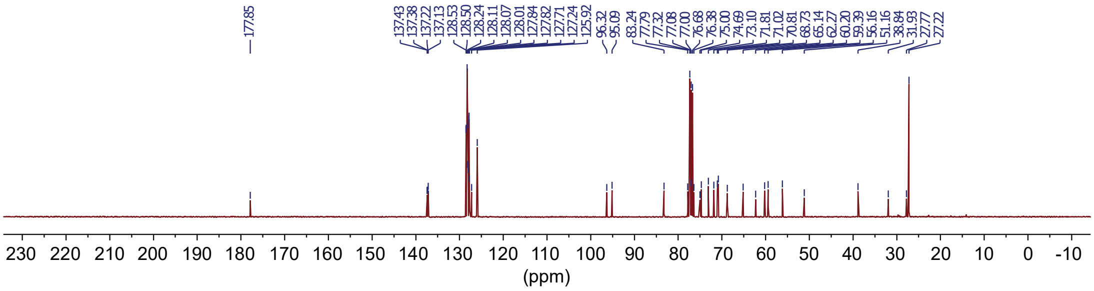

Fig. S23: ^{13}C NMR spectrum for compound **12** in $CDCl_3$ (100 MHz).

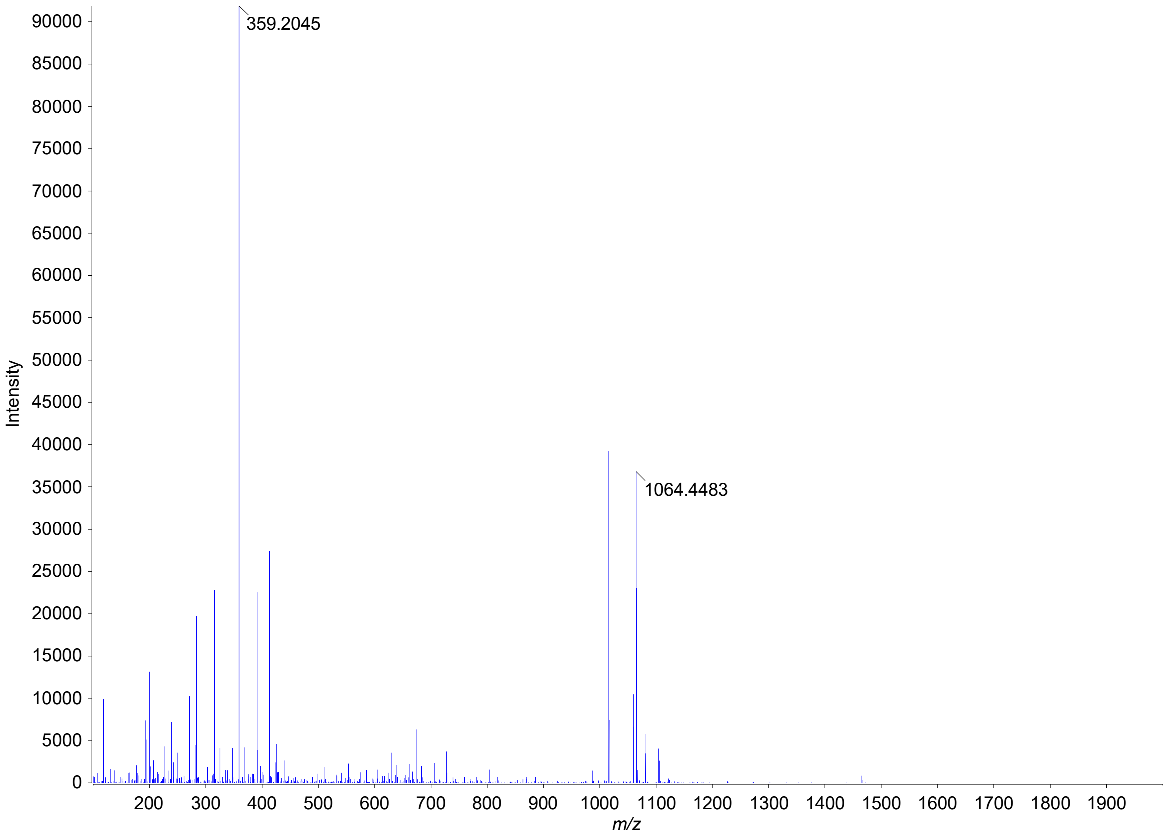

Fig. S24: Mass spectrum for compound **12**.

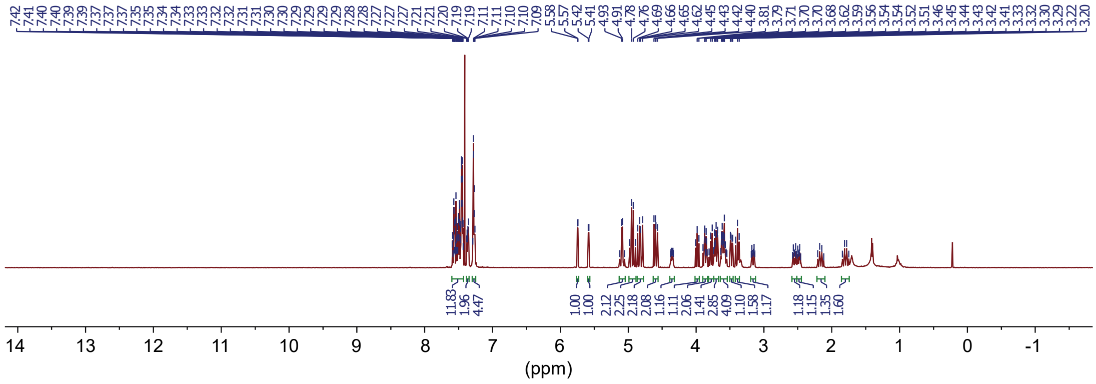

Fig. S25: ^{1}H NMR spectrum for compound **13** in $CDCl_3$ (400 MHz).

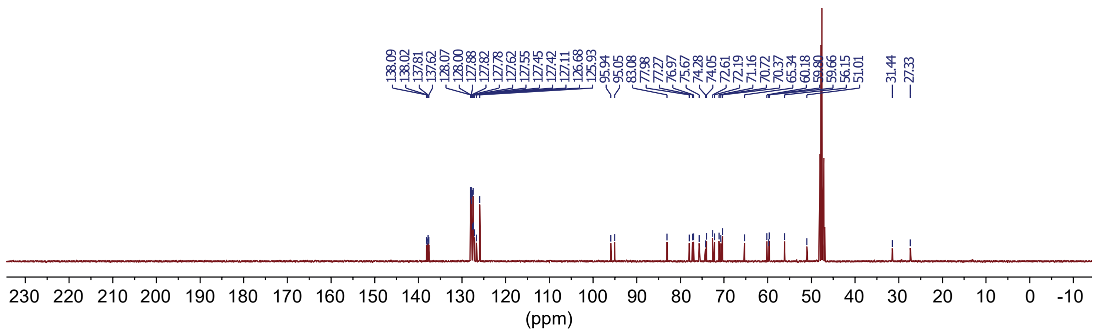

Fig. S26: ^{13}C NMR spectrum for compound **13** in CD_3OD (100 MHz).

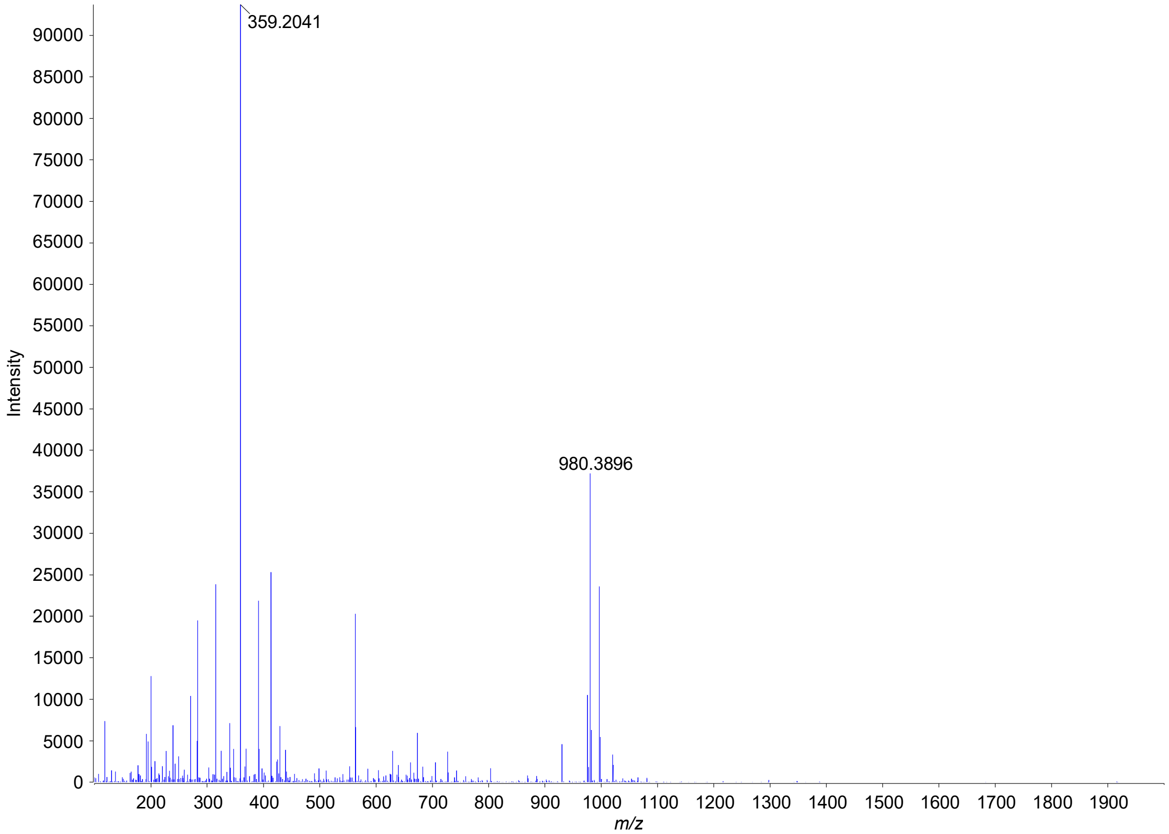

Fig. S27: Mass spectrum for compound **13**.

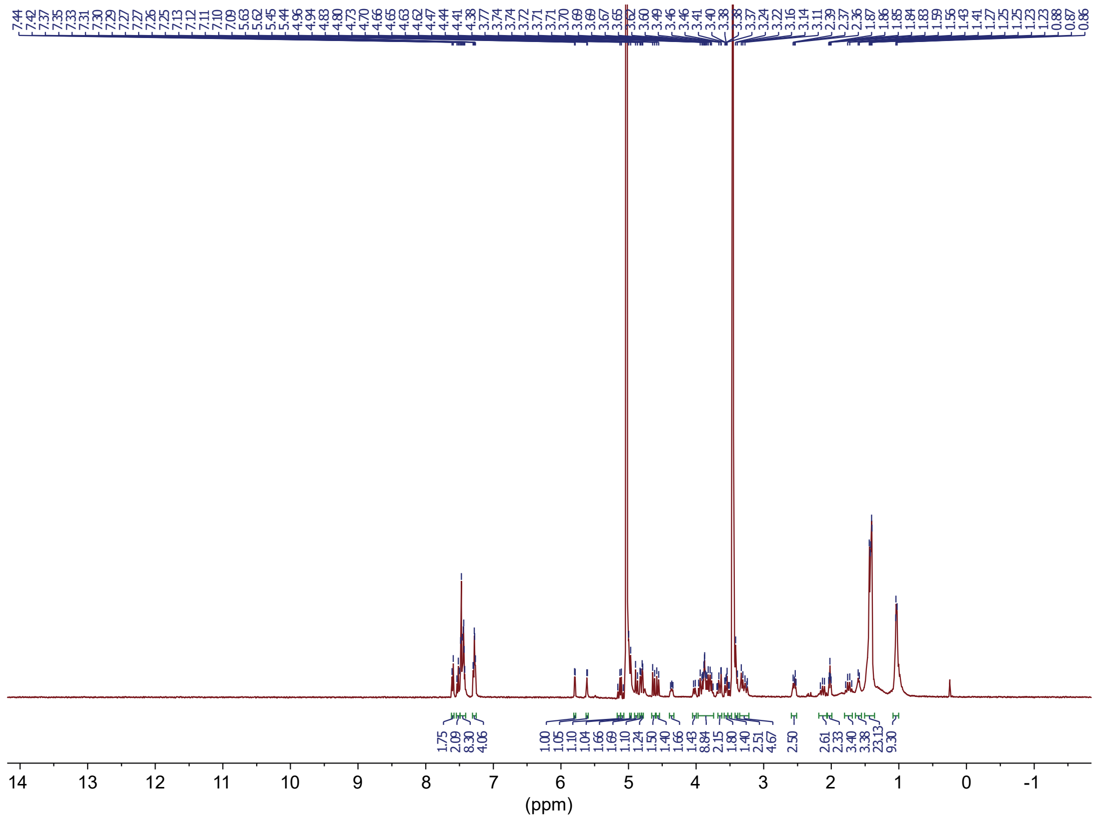

Fig. S28: ^{1}H NMR spectrum for compound **14** in CD_3OD (400 MHz).

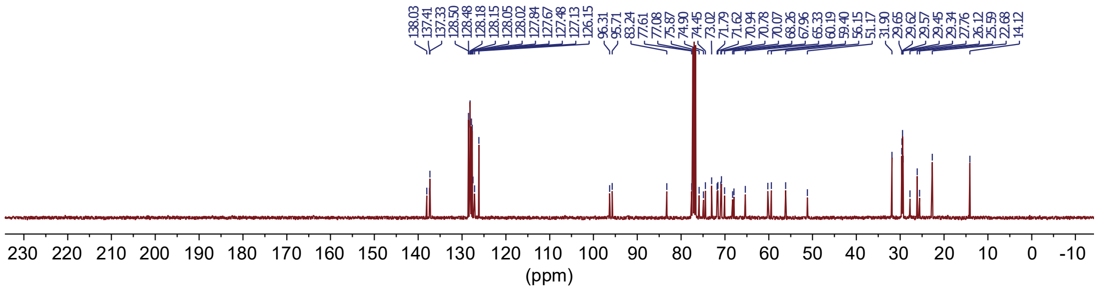

Fig. S29: ^{13}C NMR spectrum for compound **14** in $CDCl_3$ (100 MHz).

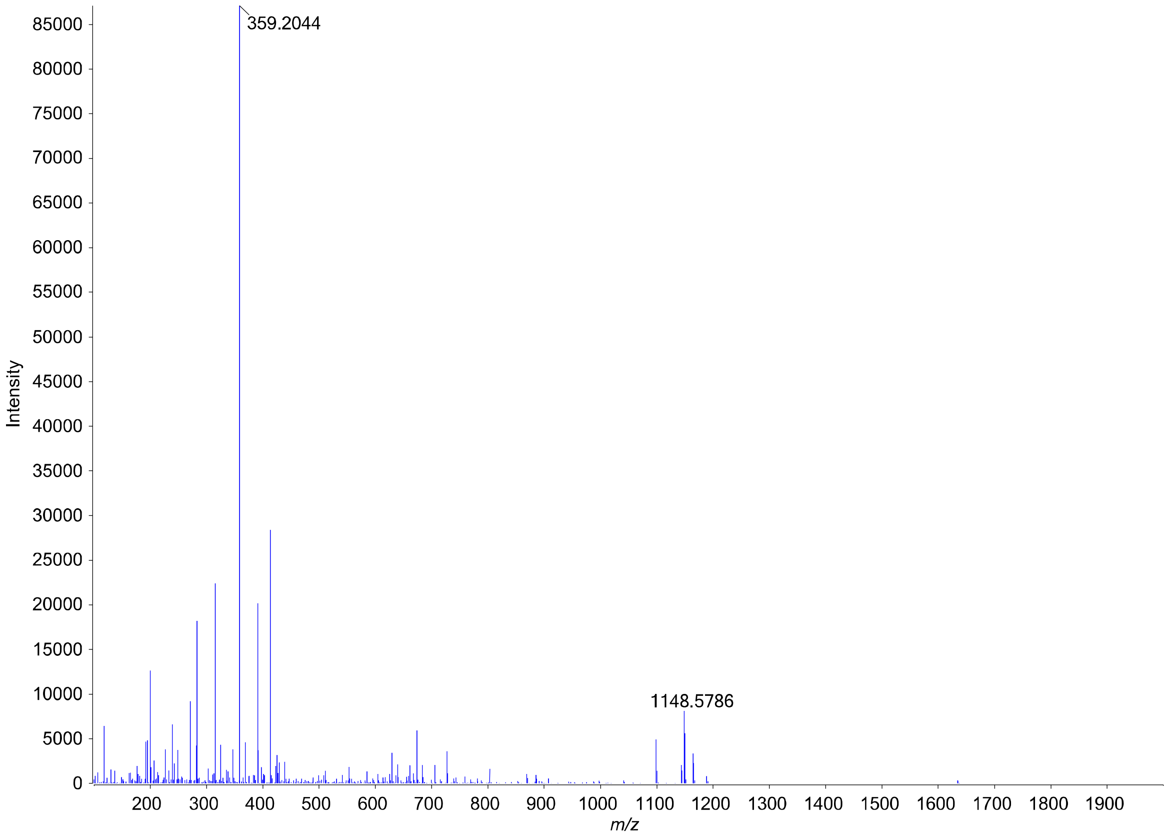

Fig. S30: Mass spectrum for compound **14**.

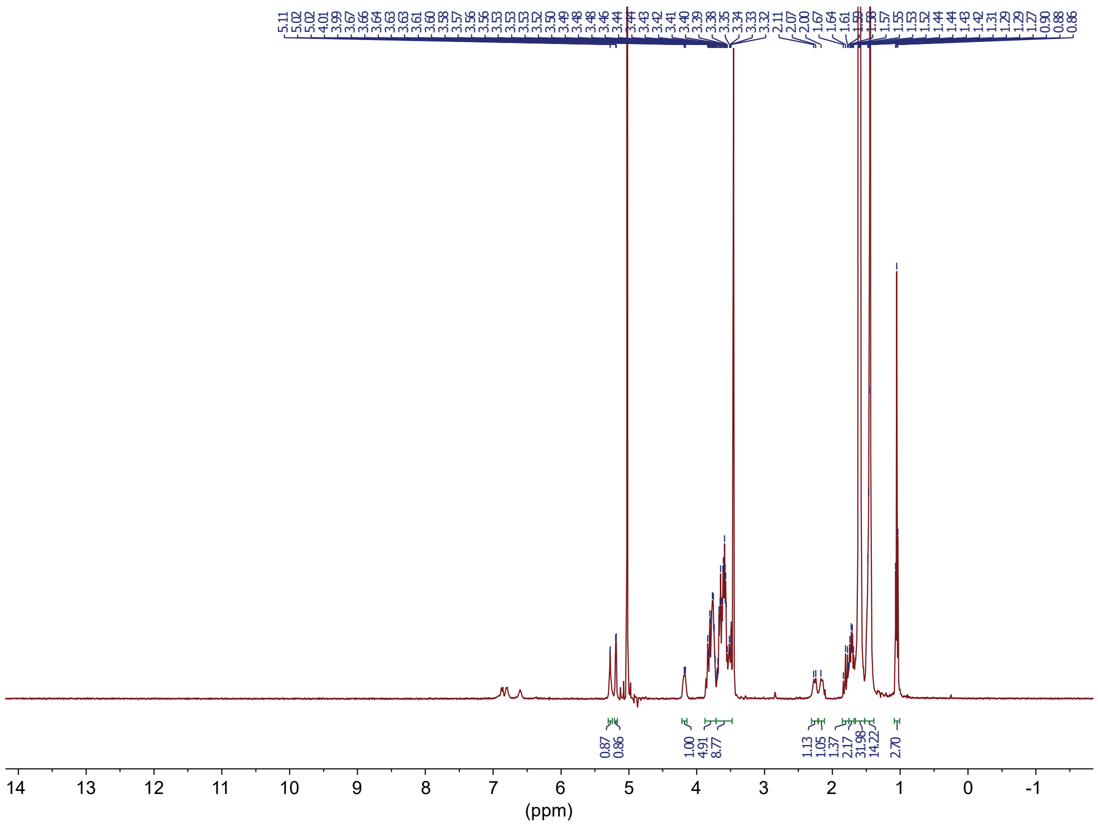

Fig. S31: ^{1}H NMR spectrum for compound **15** in CD_3OD (400 MHz).

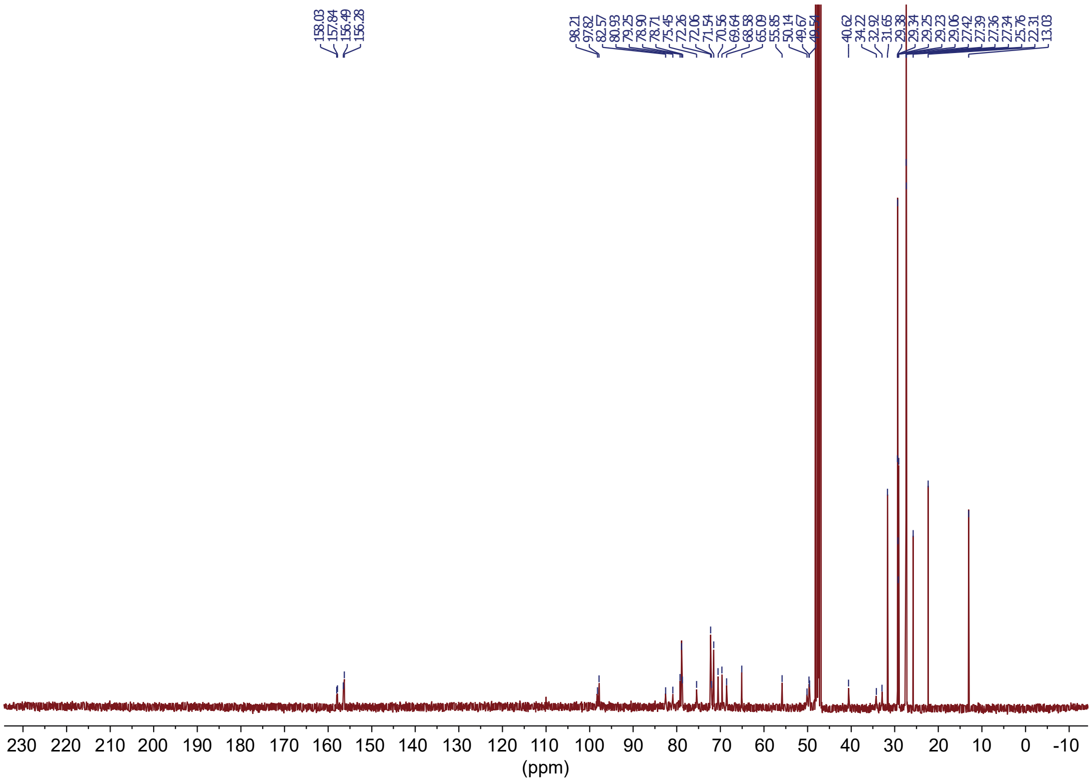

Fig. S32: ^{13}C NMR spectrum for compound **15** in CD_3OD (100 MHz).

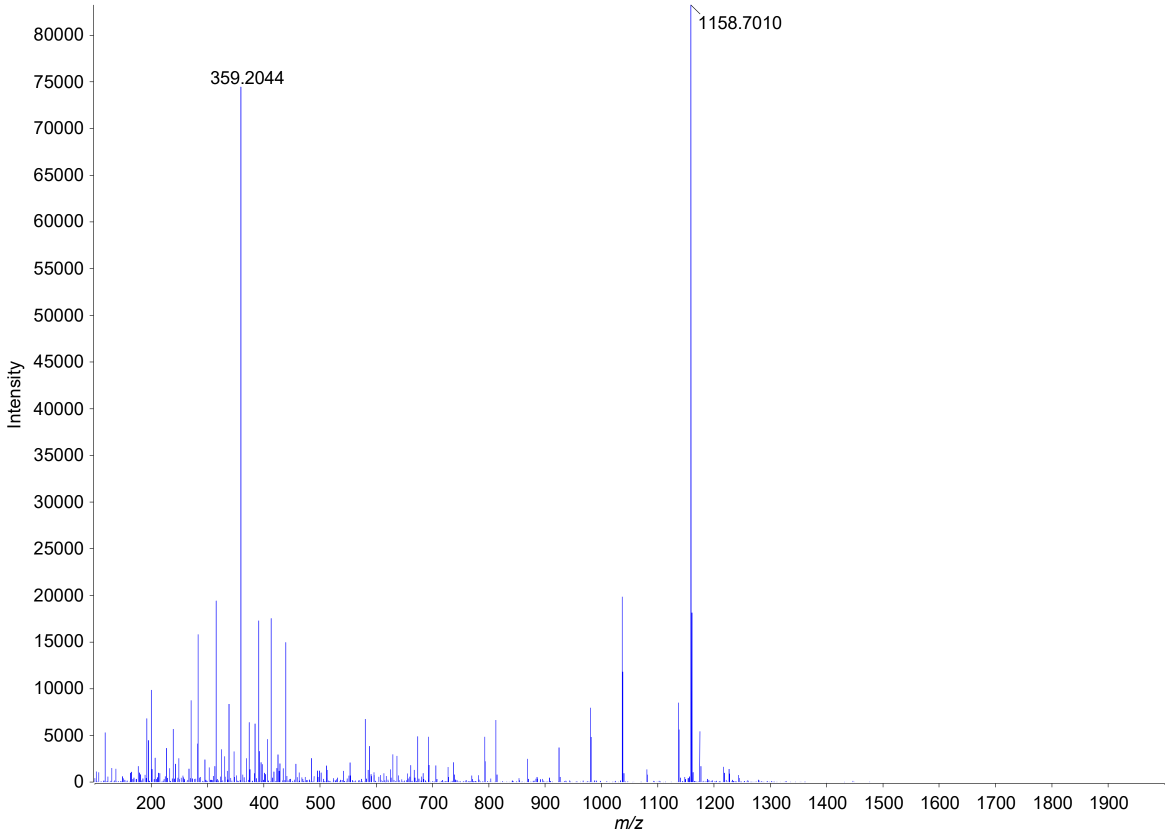

Fig. S33: Mass spectrum for compound **15**.

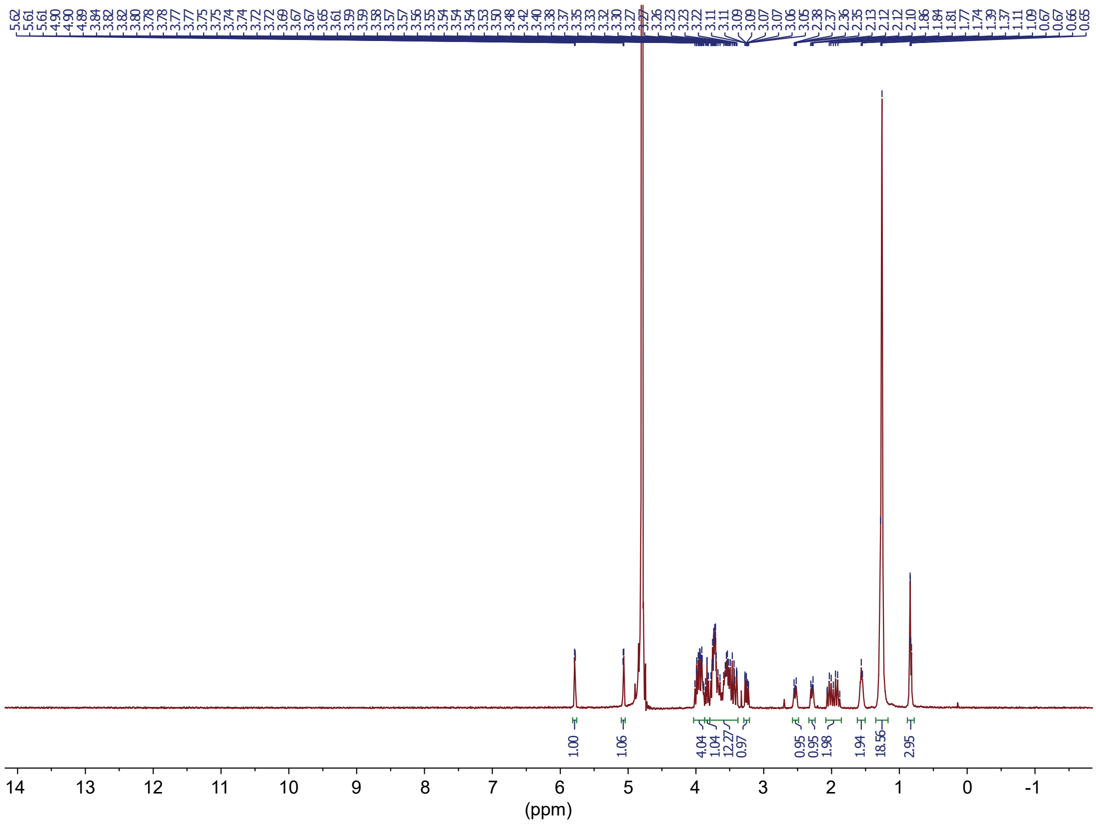

Fig. S34: ^{1}H NMR spectrum for compound **16** in D_2O (400 MHz).

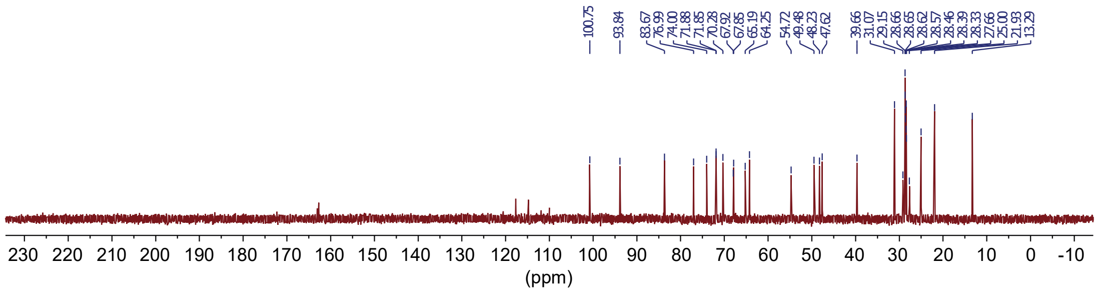

Fig. S35: ^{13}C NMR spectrum for compound **16** in D_2O (100 MHz).

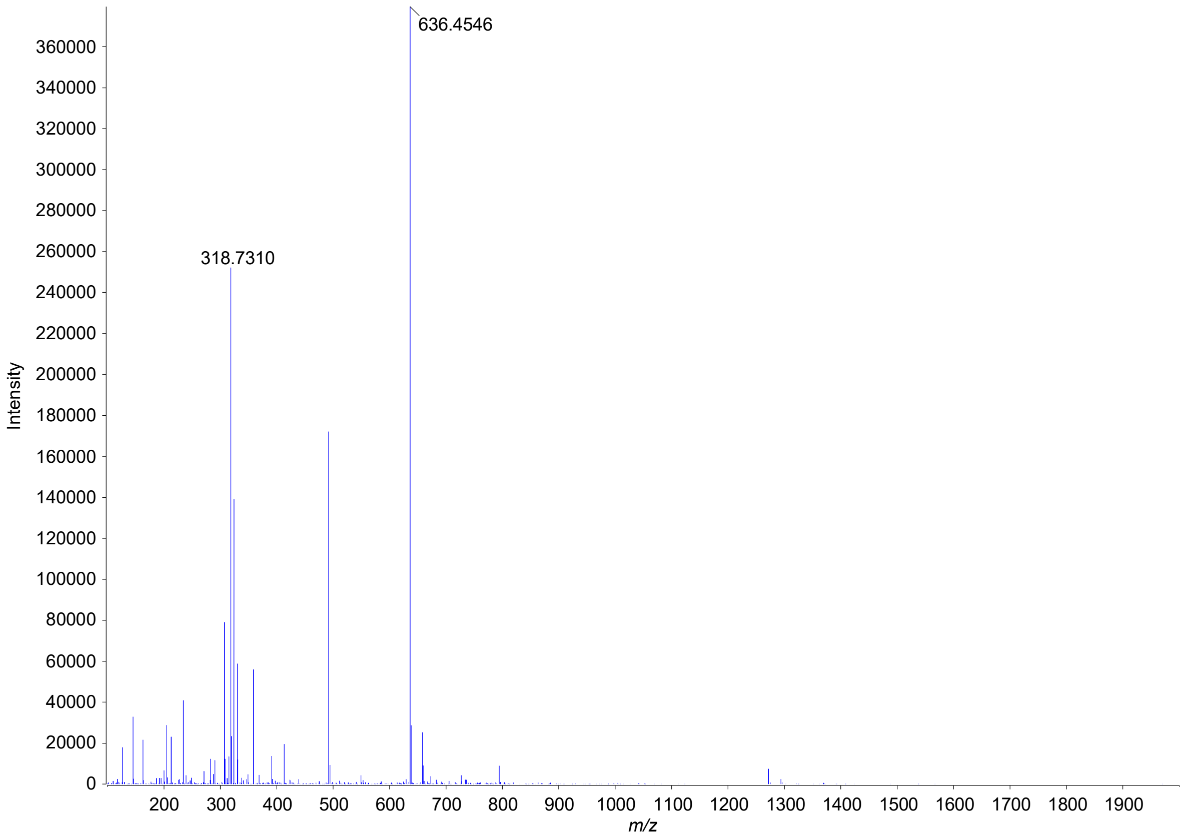

Fig. S36: Mass spectrum for compound **16**.
